# Direct ionic stress sensing and mitigation by the transcription factor NFAT5

**DOI:** 10.1126/sciadv.adu3194

**Published:** 2025-02-19

**Authors:** Chandni B. Khandwala, Parijat Sarkar, H. Broder Schmidt, Mengxiao Ma, Ganesh V. Pusapati, Frederic Lamoliatte, Maia Kinnebrew, Bhaven B. Patel, Desiree Tillo, Andres M. Lebensohn, Rajat Rohatgi

**Affiliations:** ^1^ Departments of Biochemistry and Medicine, Stanford University School of Medicine, Stanford, CA 94305, USA.; ^2^ Medical Research Council Protein Phosphorylation and Ubiquitylation Unit (MRC-PPU), School of Life Sciences, University of Dundee, Dundee, UK.; ^3^ Center for Cancer Research Genomics Core, Office of Science & Technology Resources, Office National Cancer Institute, National Institutes of Health, Building 41, RM 701D, Bethesda, MD 20892, USA.; ^4^ Laboratory of Cellular and Molecular Biology, Center for Cancer Research, National Cancer Institute, National Institutes of Health, Building 37, RM 2056C, Bethesda, MD 20892, USA.

## Abstract

Rising temperatures and water scarcity caused by climate change are increasingly exposing our cells and tissues to ionic stress, a consequence of elevated cytoplasmic ionic strength that can disrupt protein, organelle, and genome function. Here, we unveil a single-protein mechanism for ionic strength sensing and mitigation in animal cells, one that is notably different from the analogous high osmolarity glycerol kinase cascade in yeast. The Rel family transcription factor NFAT5 directly senses intracellular ionic strength using a C-terminal prion-like domain (PLD). In response to elevated intracellular ionic strength, this PLD is necessary and sufficient to coordinate an adaptive gene expression program by recruiting the transcriptional coactivator BRD4. The purified NFAT5 PLD forms condensates in response to elevated solution ionic strength in vitro, and human NFAT5 alone is sufficient to reconstitute a mammalian transcriptional response to ionic stress in yeast. We propose that ion-sensitive conformational changes in a PLD directly regulate transcription to maintain ionic strength homeostasis in animal cells.

## Teaser

Intracellular ionic strength is sensed and regulated by a prion-like domain in a transcription factor.

## INTRODUCTION

Cells are subjected to hypertonic stress when extracellular osmolarity rises above intracellular osmolarity, generating an osmotic pressure gradient across the plasma membrane that drives water efflux (*1*, *2*). Such conditions can be produced by environmental water loss (e.g., dehydration) or by an increase in the extracellular concentration of nonpermeable solutes (e.g., rising salinity). Understanding cell and tissue responses to hypertonic stress is an imperative in this era of accelerating climate change. The synergistic combination of rising temperatures (which increase evaporative water losses) and scarcity of potable water is contributing to the emergence and expansion of climate-related diseases that affect tissues vulnerable to hypertonic stress (*3*). We intentionally use the term “hypertonic stress” rather than “osmotic stress” although both terms are sometimes used interchangeably in the literature. However, these processes are distinct: Cell-permeable solutes like urea can increase osmolarity without imposing an osmotic pressure gradient or driving water movement across the membrane. Only solutes that are poorly permeable across the plasma membrane, like charged ions or sugar alcohols, can impose hypertonic stress.

The kidney serves as a sentinel organ for the damaging effects of unresolved hypertonic stress on tissues. Interstitial fluid osmolarity in the renal medulla can rise to 1200 mOsm/liter in humans and 4000 mOsm/liter in rodents, many times greater than plasma osmolarity (~300 mOsm/liter) and even greater than the osmolarity of seawater (1000 mOsm/liter) (*4*). The high interstitial osmolarity of the medulla allows the kidney to reabsorb free water and excrete urine that is more concentrated than plasma. The emerging epidemic of chronic kidney disease of unknown origin may be the consequence of failing adaptive mechanisms in the face of extreme or prolonged hypertonic stress (*3*, *5*).

**Fig. 1.**
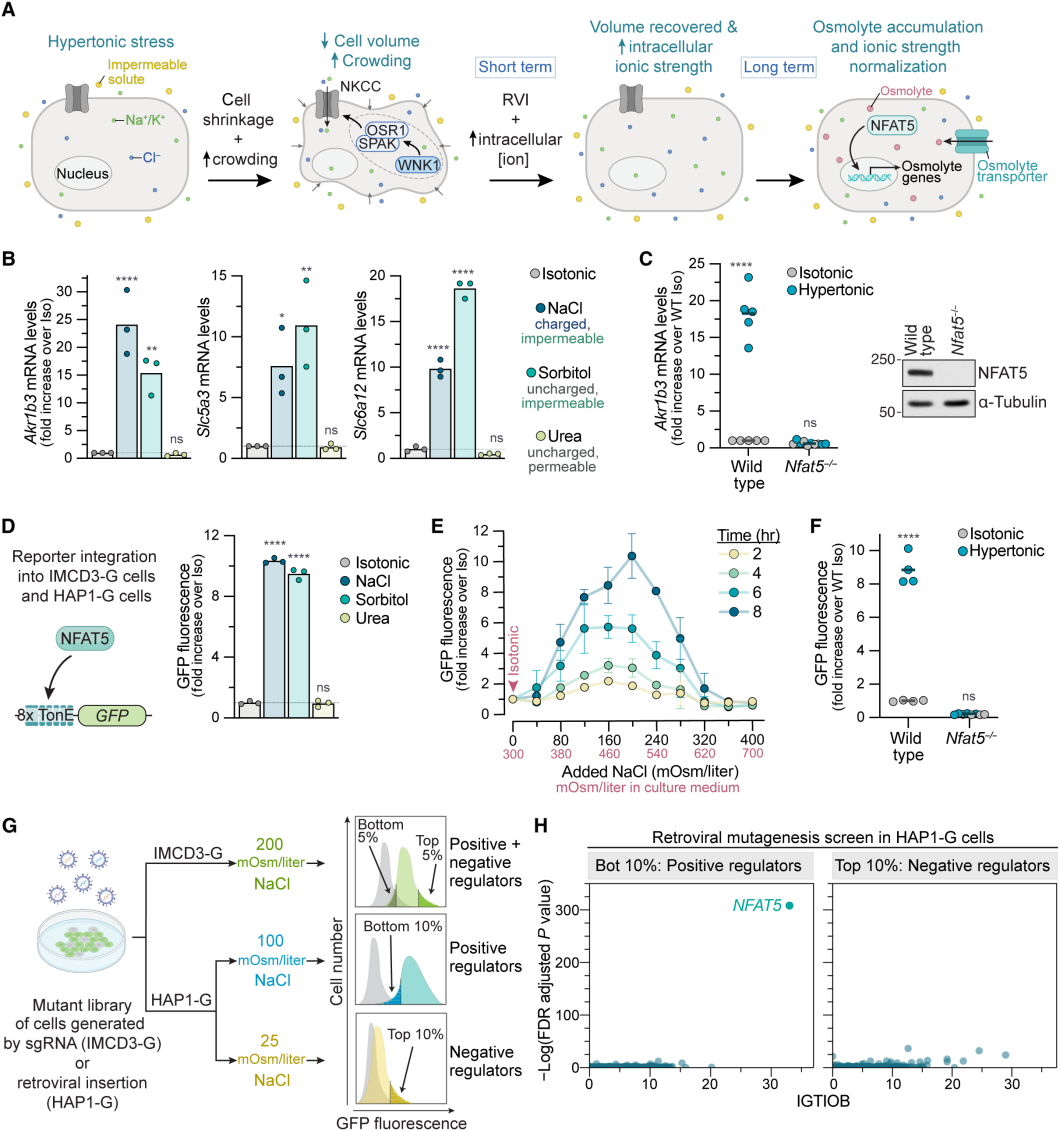
Genetic screens to identify positive and negative regulators of the transcriptional response to hypertonic stress. (**A**) Temporal sequence of cellular changes triggered by hypertonic stress. (**B**) Expression of NFAT5 target genes after 8 hours in isotonic media (300 mOsm/liter) or hypertonic media [NaCl (+200 mOsm/liter), sorbitol, or urea]. (**C**) Expression of the NFAT5 target gene *Akr1b3* in wild-type (WT) IMCD3 cells or a clonal *Nfat5*^−/−^ cell line after 8 hours in isotonic or hypertonic media [NaCl (+200 mOsm/liter)]. See fig. S1A. (**D**) GFP fluorescence in IMCD3-G reporter cells stably carrying the 8xTonE-GFP transcriptional reporter (left) to measure NFAT5 activity after 8 hours in isotonic or hypertonic media (+200 mOsm/liter). Each point depicts the median GFP fluorescence from >2000 cells. (**E**) 8xTonE-GFP activity in IMCD3-G cells in response to increasing amounts of NaCl added to isotonic media. Each point shows the mean ± SD of three independent median measurements from >2000 cells. (**F**) 8xTonE-GFP activity after exposure to hypertonic media [NaCl (+200 mOsm/liter), 8 hours] in WT or *Nfat5*^−/−^ IMCD3 cells. (**G**) Strategy for genome-wide loss-of-function screens in mouse IMCD3 and human HAP1 cells using a stably integrated 8xTonE-GFP reporter. See fig. S2A. (**H**) Results from the HAP1 screen outlined in (G). The *x* axis shows the Intronic Gene-trap Insertion Orientation Bias (IGTIOB) score (*28*), which scores the bias toward inactivating insertions in each gene, and the *y* axis shows the false discovery rate (FDR)–adjusted *P* value, reflecting the enrichment of gene trap (GT) insertions in sorted over unsorted cells. Statistics: Bars [(B) and (D)] or black horizontal lines [(C) and (F)] denote mean values calculated from independent measurements shown as points. Statistical significance was determined by a two-way analysis of variance (ANOVA) test with Sidak’s multiple comparisons posttest (*n* > 3). ****P < 0.0001, **P < 0.01, and *P < 0.05. See also figs. S1 and S2. ns, nonsignificant.

Hypertonic stress presents two distinct chemical challenges to cells at short and long timescales (Fig. 1A) (*6*, *7*). Within seconds, cells shrink as water moves down its chemical potential gradient from inside to outside the cell, causing an increase in macromolecular crowding (hereafter called “crowding stress”). Crowding stress triggers an adaptive regulatory volume increase (RVI) response that restores cell volume within minutes by promoting a rise in the cytoplasmic concentrations of charged ions (Fig. 1A) (*2*, *8*, *9*). However, RVI comes at the cost of an increase in the intracellular content of ions that is incompatible with long-term cell survival (Fig. 1A). Elevated cytoplasmic ionic strength (hereafter called “ionic stress”) disrupts the structure and function of proteins, organelles, and even the genome (*1*, *2*, *10*, *11*). Thus, cell survival in the face of persistent hypertonic stress depends on osmoregulatory systems that restore physiological intracellular ionic strength.

Osmoregulatory systems show a remarkable degree of convergent evolution across the tree of life. In organisms as diverse as plants, bacteria, fungi, fishes, invertebrates, and mammals, the adaptation to hypertonic stress depends on the intracellular accumulation of small organic osmolytes (*1*, *12*, *13*). Osmolytes are nonmembrane-permeable molecules that are uncharged or zwitterionic and include polyhydric alcohols, free amino acids, and methylamines. Osmolytes have stabilizing or neutral effects on protein structure and activity across a wide range of concentrations. The exchange of ions for osmolytes balances the osmotic pressure inside and outside the cell (preventing water depletion) while restoring intracellular ionic strength to the narrow range required for protein and organelle function (Fig. 1A). An inability to accumulate osmolytes leads to massive atrophy and ultimately failure of the renal medulla, a tissue chronically exposed to hypertonic stress (*4*, *14*, *15*).

An exemplar eukaryotic osmoregulatory system is the high-osmolarity glycerol (HOG) pathway, intensively studied in the yeast *Saccharomyces cerevisiae* (*16*, *17*). Membrane proteins presumed to be sensors of hypertonic stress activate a cytoplasmic kinase cascade centered on the p38/mitogen-activated kinase (MAPK) Hog1, ultimately leading to transcriptional activation of genes that promote the synthesis and accumulation of glycerol, the major osmolyte in *S. cerevisiae*. However, many components of the HOG pathway are not conserved in multicellular animals. Nearly three decades ago, the Rel family transcription factor NFAT5 (also known as tonicity enhancer binding protein or TonEBP) was identified as the master regulator of the response to hypertonic stress in mammals (Fig. 1A) (*18*, *19*). NFAT5 is conserved across Bilateria, found in insects, vertebrates, molluscs, and echinoderms, but is absent in fungi (*20*). NFAT5 activates the transcription of many osmoadaptive genes, including those that promote osmolyte accumulation and those that encode heat shock proteins to manage protein folding stress (*21*–*23*). We set out to answer two enduring mysteries in the mammalian osmoregulatory response: How is hypertonic stress sensed and how is this signal transmitted to NFAT5?

## RESULTS

### Genetic screens fail to identify other genes that regulate NFAT5 activity

Mouse inner medullary collecting duct 3 (IMCD3) cells, derived from the renal medulla, have been extensively used to study cellular responses to hypertonic stress (*24*). Increasing the osmolarity of IMCD3 culture media by adding charged (NaCl) or uncharged (sorbitol) molecules that are membrane impermeable increased the expression of three established NFAT5 target genes that promote intracellular osmolyte accumulation: *Akr1b3* (aldol reductase, required for sorbitol synthesis), *Slc5a3* (a myo-inositol importer), and *Slc6a12* (a betaine importer) (Fig. 1, B and C, and fig. S1A) (*22*). Urea, a cell-permeable molecule, was unable to activate transcription of these genes (Fig. 1B) (*21*). Thus, NFAT5 is not activated by increasing osmolarity alone but rather by a difference in osmolarity across the plasma membrane.

We took a genetic approach to identifying components of the NFAT5 pathway in IMCD3 cells and human haploid cells (HAP1) using a fluorescent transcriptional reporter of NFAT5 activity [8xTonE–green fluorescent protein (GFP); Fig. 1D] (*25*). GFP fluorescence in IMCD3 or HAP1 cells stably expressing 8xTonE-GFP (hereafter IMCD3-G and HAP1-G cells) increased in response to hypertonic stress in a dose-, time-, and NFAT5-dependent manner (Fig. 1, D to F, and fig. S1B). The kinetics of GFP mRNA accumulation resembled those of endogenous NFAT5 target genes (fig. S1C). As noted previously (*26*) but not further investigated in our current study, increasing hypertonic stress resulted in a bell-shaped change in NFAT5 activity (Fig. 1E and fig. S1D). Most of our experiments were conducted using conditions in the ascending phase of this dose-response curve.

Using the stably expressed 8xTonE-GFP reporter for phenotypic enrichment, we conducted a loss-of-function CRISPR screen in IMCD3-G cells designed to find positive and negative regulators of NFAT5 activity (Fig. 1G) (*27*). In parallel, we took advantage of the haploid genome of HAP1-G cells to conduct two retroviral insertional mutagenesis screens (Fig. 1G and fig. S2A) (*28*). *NFAT5* itself was the most statistically significant hit and the only common positive regulator identified in both the CRISPR and haploid screens (Fig. 1H and fig. S2, B and C). Targeted disruption of 50 of the top CRISPR screen hits using independent single guide RNAs (sgRNAs) had much smaller effects on 8xTonE-GFP activation compared to the disruption of *Nfat5* itself (fig. S2, D to F).

Unexpectedly, genetic screens in cell lines from two different species using two different mutagenesis methods identified NFAT5 as the only component required for the activation of a transcriptional reporter of hypertonic stress. We acknowledge that redundant and essential genes would not be expected to score as hits.

### NFAT5 alone is sufficient to reconstitute a mammalian transcriptional response to hypertonic stress in *S. cerevisiae*

Our genetic screens led us to consider the hypothesis that NFAT5 alone may be sufficient to mediate the transcriptional response to hypertonic stress in mammals; no elaborate upstream signaling pathway analogous to the yeast HOG pathway is involved. If this is the case, then NFAT5 should be the only component required to endow an organism lacking *Nfat* genes with a mammalian-like hypertonic stress response. We chose *S. cerevisiae* for several reasons. First, fungi and animals diverged nearly 1.5 billion years ago, and their osmoregulatory pathways are quite different: *S. cerevisiae* lacks an *Nfat5*-related gene (*20*). *S. cerevisiae* has a cell wall and is thought to sense hypertonic stress through changes in turgor pressure using membrane protein sensors that are not conserved in mammals (*16*). Last, the human *NFAT5* cDNA was originally cloned using a one-hybrid screen, showing that NFAT5 can activate the yeast transcriptional machinery (*18*).

**Fig. 2.**
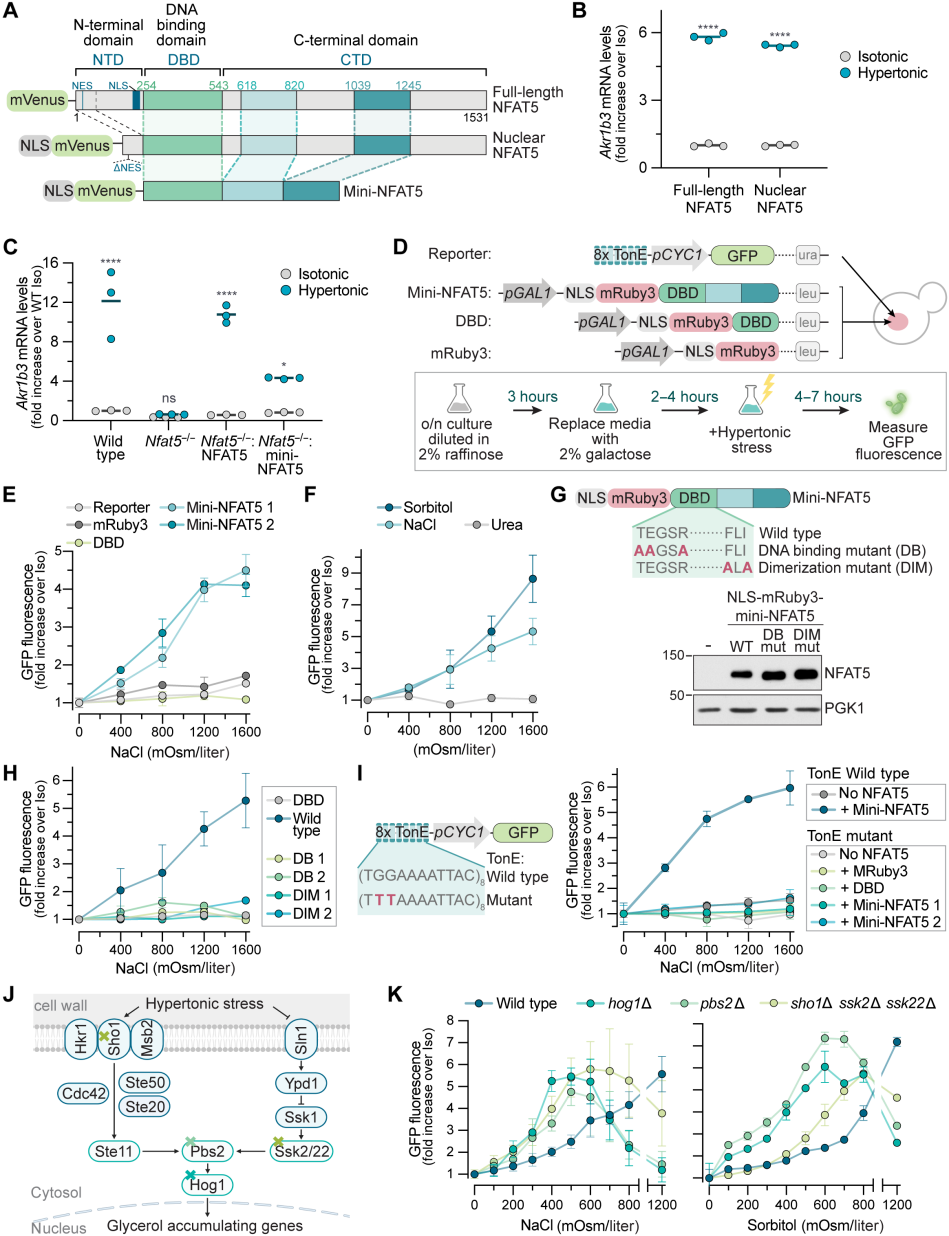
NFAT5 can be activated by hypertonic stress in *S. cerevisiae*. (**A**) Domain structures of mVenus-tagged human full-length, nuclear, and mini variants of NFAT5. Nuclear NFAT5 was constitutively targeted to the nucleus by removal of its endogenous nuclear localization signal (NLS) and nuclear export signal (NES) sequences and addition of a strong foreign NLS. (**B** and **C**) Expression of an NFAT5 target gene in *Nfat5^−/−^* IMCD3 cells stably expressing NFAT5 variants [see (A)] in isotonic or hypertonic [NaCl (+200 mOsm/liter), 8 hours] media. See fig. S3A for protein abundances. (**D** to **F**) Structure of the 8xTonE-*pCYC1*-GFP reporter and galactose-inducible (*pGAL1*) mini-NFAT5 variant genes integrated into WT W303a yeast cells. Box (D) shows the workflow used to measure reporter activity after expression of mRuby3, mRuby3-DBD, or mRuby3-mini-NFAT5 (E) or exposure of cells expressing mRuby3-mini-NFAT5 to various solutes (F). All solutes were added at the indicated concentrations to complete synthetic media (CSM). (**G** and **H**) 8xTonE-*pCYC1*-GFP reporter activity (H) in yeast cells expressing DNA binding (DB) or dimerization (DIM) mutants of NLS-mRuby3-mini-NFAT5 (G). (**I**) Response of a mutant 8xTonE-*pCYC1*-GFP reporter (left) known be impaired in binding to NFAT5 to increasing hypertonic stress. (**J**) The HOG (high-osmolarity glycerol) pathway in *S. cerevisiae*. Colored X’s denote three different genes or gene sets that were deleted to disrupt the pathway at various levels: *HOG1*, *PBS2*, or the combined deletion of *SSK2*, *SSK22*, and *SHO1*. (**K**) 8xTonE-*pCYC1*-GFP reporter activity in WT, *hog1*Δ, *pbs2*Δ, or *ssk*2Δ *ssk22*Δ *sho1*Δ cells (also expressing mini-NFAT5). Statistics: Each point [(E), (F), (H), (I), and (K)] shows the mean ± SD of >3 median measurements, each from >5000 cells. Solid horizontal lines [(B) and (C)] denote mean values (*n* = 3). [(B) and (C)] Two-way ANOVA test with Sidak’s multiple comparisons posttest (*n* > 3). ****P < 0.0001 and *P < 0.05. See also fig. S3.

NFAT5 is localized in both the nucleus and cytoplasm of IMCD3 cells under isotonic conditions; in response to hypertonic stress, it accumulates in the nucleus and activates target genes (fig. S3, A and B) (*29*). To determine whether nuclear accumulation or transactivation was the regulated step, we constructed a variant (nuclear NFAT5, Fig. 2A) constitutively localized in the nucleus by fusion to a foreign nuclear localization sequence and removal of nuclear export sequences (fig. S3, A and C). Forced nuclear localization was not sufficient to activate nuclear NFAT5, which still required hypertonic stress to activate target genes (Fig. 2B). We conclude that nuclear localization is necessary but not sufficient to activate NFAT5. This property distinguishes NFAT5 from NFATc1-4 proteins, which are controlled at the nuclear accumulation step. We thus focused our attention on the capacity of NFAT5 to activate transcription in the nucleus.

Expression of full-length NFAT5, 1531 amino acids in length, was challenging in *S. cerevisiae.* Therefore, we engineered a minimal NFAT5 (hereafter “mini-NFAT5”) that recapitulated the key features of NFAT5 in IMCD3 cells but was compact enough to express in yeast (Fig. 2A). Mini-NFAT5 contained a fluorescent protein for detection, the native Rel-homology DNA binding domain (DBD), and a yeast NLS to drive nuclear localization (fig. S3, A and D), enabling us to study the transactivation step in the nucleus in isolation from effects on nuclear/cytoplasmic trafficking. On the basis of a prior truncation analysis, mini-NFAT5 included two segments from the C-terminal domain (CTD) (Fig. 2A) that have been shown to be sufficient to confer hypertonicity-activated reporter gene transcription (*30*). We confirmed that mini-NFAT5 was sufficient to drive activation of endogenous NFAT5 target genes in response to hypertonic stress (Fig. 2C).

To test the activity of mini-NFAT5 in *S. cerevisiae*, we stably expressed (i) a variant of the 8xTonE-GFP reporter constructed from a minimal yeast promoter (8xTonE-*pCYC1*-GFP) and (ii) mRuby3 tagged mini-NFAT5, codon optimized for yeast expression, driven by a galactose-inducible promoter (Fig. 2D). *S. cerevisiae* strains expressing both transgenes were treated with galactose to induce expression of mini-NFAT5 (fig. S3E) and then subjected to increasing levels of hypertonic stress by the addition of NaCl to culture media. In multiple independent clonal strains, NaCl induced the dose-dependent activation of the 8xTonE-*pCYC1-*GFP reporter as measured by GFP fluorescence (Fig. 2E), mRNA abundance (fig. S3F), or protein abundance (fig. S3G). Reporter activation in yeast was induced only by nonmembrane-permeable solutes (NaCl and sorbitol) that exert an osmotic pressure gradient across the plasma membrane but not by membrane-permeable molecules that only increase osmolarity (Fig. 2F).

We confirmed the specificity of the *S. cerevisiae* mini-NFAT5 response in several ways. First, the NFAT5 CTD was required for activity: mRuby3 alone or mRuby3 fused to the isolated DBD of NFAT5 were both inactive (Fig. 2E). Point mutations in the NFAT5 DBD that abolish activity by either preventing DNA binding or dimerization (Fig. 2G) (*31*) abolished the ability of mini-NFAT5 to activate the 8xTonE-*pCYC1*-GFP reporter (Fig. 2H) without changing protein abundance or nuclear localization (Fig. 2G and fig. S3H). Last, mutations in the TonE binding sites of the 8xTonE-*pCYC1*-GFP reporter known to abolish NFAT5 binding in mammalian systems also abrogated the mini-NFAT5 transcriptional response to hypertonic stress (Fig. 2I) (*32*). Together, these results show that the sequence requirements for NFAT5 activity are similar in both *S. cerevisiae* and mammalian systems, supporting a shared mechanism of transcriptional activation in response to hypertonic stress.

Hypertonic stress applied to *S. cerevisiae* activates the HOG pathway. Thus, it was important to test whether HOG signaling mediates activation of mammalian mini-NFAT5 expressed in yeast. We disrupted both branches of the HOG pathway at three different levels using previously established strategies: disruption of *HOG1* itself, disruption of *PBS2*, encoding a MAPKK and scaffold protein, and the combined disruption of *SHO1*, *SSK2*, and *SSK22* (Fig. 2J) (*17*, *33*). As expected, each of these mutations abrogated activation of HOG pathway target genes in response to hypertonic stress (fig. S3I). However, in each of these three mutant backgrounds, mini-NFAT5 was still able to activate transcription of the 8xTonE-*pCYC1*-GFP reporter in response to hypertonic stress, showing that the HOG pathway activity is dispensable for NFAT5 signaling in *S. cerevisiae* (Fig. 2K).

The sensitivity of mini-NFAT5 to hypertonic stress was significantly enhanced in strains with disabled HOG signaling—equivalent levels of 8xTonE-*pCYC1*-GFP reporter activation were achieved at lower concentrations of added NaCl or sorbitol. Reporter activity in *hog1*Δ cells declined at higher concentrations of NaCl, resulting in a bell-shaped dose-response curve that qualitatively resembled the curve observed in mammalian cells (Fig. 1E). The increased sensitivity of NFAT5 to hypertonic stress in *hog1*Δ cells may be caused by the impaired ability of these cells to regulate intracellular ion homeostasis. Consistent with this notion, the intracellular concentration of Na^+^ ions was elevated in *hog1*Δ cells, measured using either a fluorescent Na^+^ sensor or inductively coupled plasma optical emission spectroscopy (fig. S3, J and K). Therefore, the absence of a parallel osmoregulatory mechanism in *hog1*Δ cells results in a stronger stimulus for mini-NFAT5 activation.

### Intracellular ionic strength is the signal for NFAT5 activation

**Fig. 3.**
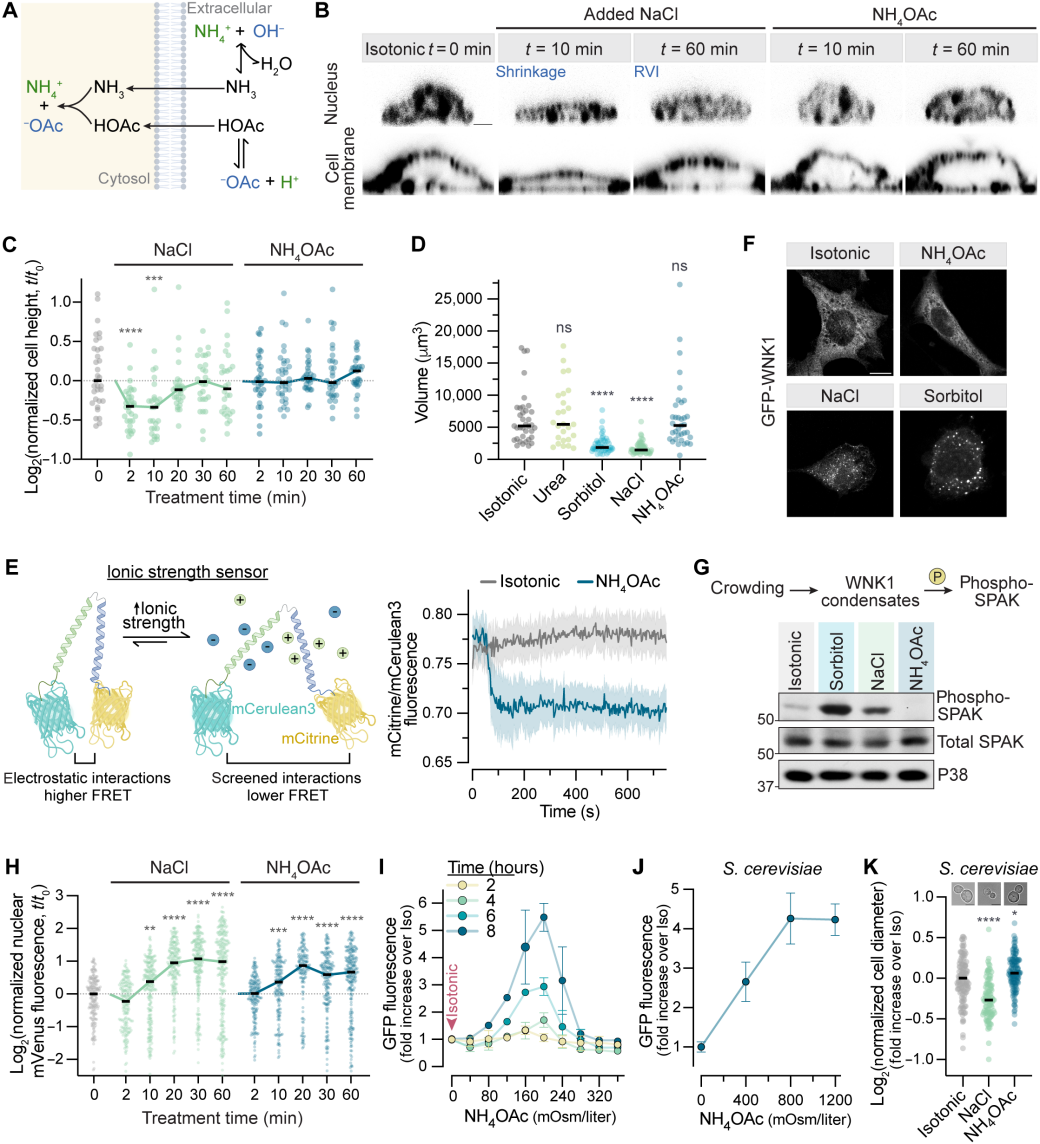
NFAT5 is activated by ionic stress. (**A**) Mechanism of ammonium acetate (NH_4_OAc) permeation into cells. (**B** and **C**) Confocal images (*xz* plane) of IMCD3 cells exposed to NaCl or NH_4_OAc (+200 mOsm/liter) showing nuclei [4′,6-diamidino-2-phenylindole (DAPI), top] or the plasma membrane (CellMask, bottom). Cell heights (*n* > 28 per condition) calculated from such images are shown at various time points after solute addition (C). (**D**) Volume of single IMCD3 cells (*n* > 26 per condition) 10 min after the addition of various solutes (+200 mOsm/liter). (**E**) Change in the mean (±SEM, *n* = 20) fluorescence ratio from a genetically encoded ionic strength sensor (left) expressed in IMCD3 cells exposed to NH_4_OAc (200 mOsm/liter). (**F** and **G**) Distribution of GFP-WNK1 stably expressed in *Wnk1*^−/−^ IMCD3 cells 30 min after the addition of various solutes (+400 mOsm/liter, 30 min). (G) Abundances of phosphorylated and total SPAK in IMCD3 cells 30 min after the addition of various solutes (+400 mOsm/liter). (**H**) Nuclear mVenus-NFAT5 fluorescence (*n* > 145 per condition) in *Nfat5*^−/−^:mVenus-NFAT5 IMCD3 cells after the addition of NaCl or NH_4_OAc (+200 mOsm/liter). See fig. S4A. (**I**) Dose-response relationship between NH_4_OAc concentration and 8xTonE-GFP reporter activity. Each point shows the mean ± SD of three median measurements from >2000 cells. (**J**) 8xTonE-*pCYC1*-GFP reporter activity in yeast cells expressing mini-NFAT5 (Fig. 2D) in response to NH_4_OAc. Each point shows the mean ± SD of six median measurements from >5000 cells. (**K**) The diameter of yeast cells (*n* > 98 per condition) 5 min after the addition of NaCl (1200 mOsm/liter) or NH_4_OAc. Scale bars, 2 μm (B) and 10 μm (F). Statistics: (C), (D), (H), and (K) show single-cell measurements and the population median. Kruskal-Wallis test with Dunn’s multiple comparisons test. ****P < 0.0001, ***P < 0.001, and **P < 0.01. See also fig. S4.

Hypertonic stress causes two distinct changes (Fig. 1A) in cell physiology that have been implicated in the activation of NFAT5 in different studies (*2*, *34*–*38*): a decrease in cell volume (which reduces tension on the plasma membrane and increases macromolecular crowding) and an elevation in intracellular ionic strength (*6*, *7*). To identify the specific physicochemical signal that activates NFAT5, we sought to disentangle cell volume changes from changes in intracellular ionic strength. Addition of extracellular NaCl or sorbitol affects both and so cannot be used to cleanly distinguish between these two signals. Nearly a century ago, cell physiologists noted that ammonium salts of weak acids like acetate and benzoate readily penetrate cells without causing a change in cell volume (*39*) or intracellular pH (*40*). When ammonium acetate (NH_4_OAc) is added to the extracellular medium, the uncharged products of its hydrolysis (NH_3_ and HOAc) enter cells and recombine to form the salt (Fig. 3A). Thus, cell-permeant salts can increase intracellular ionic strength without causing cell shrinkage.

Unlike NaCl or sorbitol, NH_4_OAc did not cause shrinkage of IMCD3 cells but did increase intracellular ionic strength as measured by a genetically encoded, fluorescence resonance energy transfer–based ionic strength sensor (Fig. 3, B to E) (*41*). NH_4_OAc also failed to induce either the phase separation or activation of with-no-lysine kinase 1 (WNK1), a sensor of macromolecular crowding that initiates the RVI response (Figs. 1A and 3, F and G) (*9*). Thus, both morphometric and biochemical analyses confirmed that NH_4_OAc does not cause cell shrinkage or increase macromolecular crowding. However, the addition of NH_4_OAc led to NFAT5 activation, as measured by nuclear accumulation, 8xTonE-GFP induction, and target gene transcription (Fig. 3, H and I, and fig. S4, A and B). NH_4_OAc also triggered the activation of mini-NFAT5 in *S. cerevisiae*, without causing any changes in yeast cell size (Fig. 3, J and K).

To confirm our results using NH_4_OAc, we deconvolved the effects of intracellular ionic strength and cell volume changes using two established but completely orthogonal methods. First, we used a protocol designed to isotonically shrink cells by cycling them from hypotonic to isotonic media in the presence of RVI inhibitors (fig. S4, C to E) (*9*, *34*). Isotonic shrinkage triggered WNK1 phase separation (fig. S4H), showing that it increases macromolecular crowding, but failed to result in NFAT5 activation (fig. S4, F and G). Second, we used nystatin, a reversible ionophore, to increase the intracellular ion concentration without reducing cell volume (fig. S4, I to K) (*42*). Under these conditions, we observed NFAT5 activation (fig. S4, L and M) but not WNK1 condensation (fig. S4N).

Cell-permeant salts like NH_4_OAc provide a simple way to increase intracellular ionic strength and impose ionic stress without altering cell volume and macromolecular crowding. Using multiple independent methods, our results established that NFAT5 activation is triggered by ionic stress. We next sought to understand how increased ionic strength regulates transactivation by NFAT5 in the nucleus.

### NFAT5 forms biomolecular condensates in response to hypertonic and ionic stress

**Fig. 4.**
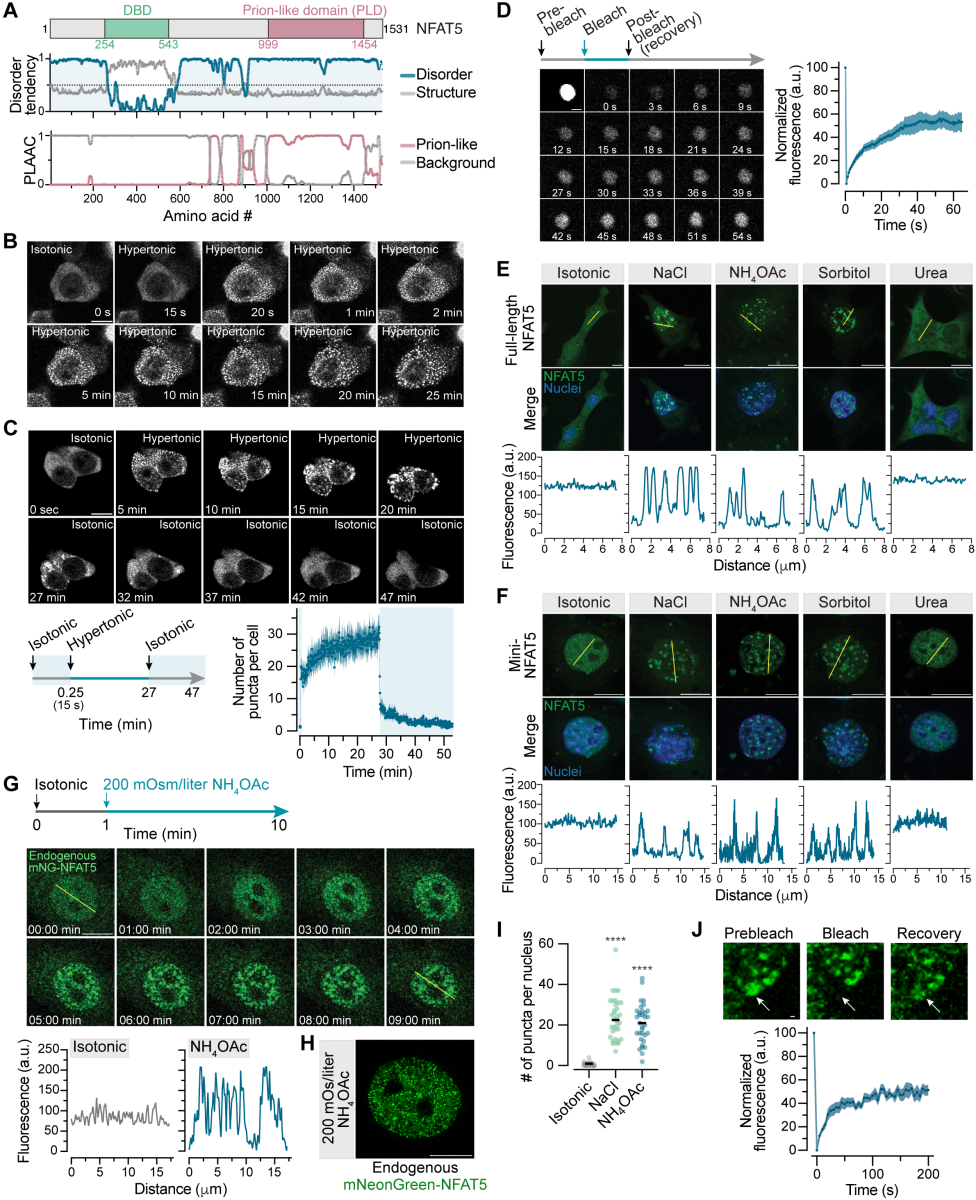
NFAT5 forms biomolecular condensates in cells exposed to hypertonic or ionic stress. (**A**) NFAT5 has a predicted prion-like domain (PLD) and a structured DBD embedded within IDRs (gray). (**B** and **C**) Snapshots from live cell imaging of HEK293T cells transiently transfected with GFP-NFAT5 and subjected to hypertonic stress [NaCl (+100 mOsm/liter)]. Mean (±SEM, *n* = 15) number of droplets per cell is shown on the graph (C, right) during a isotonic-hypertonic-isotonic stress cycle [(C), left]. (**D**) Snapshots (left) and recovery curve (right, mean ± SEM, *n* = 9) from a fluorescence recovery after photobleaching (FRAP) experiment on GFP-NFAT5 condensates in HEK293T subjected to hypertonic stress [NaCl (+100 mOsm/liter), 30 min]. (**E** and **F**) Subcellular distributions of full-length GFP-NFAT5 (E) or mVenus-mini-NFAT5 (F) stably expressed from a single locus in *Nfat5*^−/−^ IMCD3 cells after the addition of various solutes (+200 mOsm/liter, 30 min). (**G**) Live cell time course of NH_4_OAc treated IMCD3 cells carrying NFAT5 tagged at its endogenous genomic locus with mNeonGreen (mNG) [see fig. S6 (C to E)]. In (E) to (G), line scans show fluorescence intensity traces along the trajectories of the yellow line in the images. (**H** and **I**) Endogenously tagged mNG-NFAT5 nuclear condensates in IMCD3 cells [see fig. S6 (C) to (E)] were imaged by structured illumination microscopy (H) and enumerated (I, *n* ~ 34 cells with median indicated) after the addition of NaCl or NH_4_OAc (+200 mOsm/liter, 30 min). Kruskal-Wallis test with Dunn’s multiple comparisons posttest. ****P < 0.0001. (**J**) FRAP images and recovery curve (*n* = 13; mean ± SEM) of endogenous mNG-NFAT5 puncta in IMCD3 cells subjected to ionic stress [NH_4_OAc (+200 mOsm/liter), 30 min]. Scale bars, 10 μm [(B), (C), (E), (F), (G), and (H)] and 1 μm [(D) and (J)]. See also figs. S5 and S6. a.u., arbitrary units.

Nearly the entire 1531–amino acid NFAT5 protein (with the exception of the 291–amino acid DBD) is predicted to be disordered (Fig. 4A and fig. S5A). Intrinsically disordered regions (IDRs), often characterized by low sequence complexity, are a common feature of the transactivation domains (ADs) of sequence specific transcription factors (TFs). These ADs are thought to promote the initiation or elongation activity of RNA polymerase II (Pol II) by recruiting coactivators like the multisubunit Mediator complex (*43*). Multivalent homotypic and heterotypic IDR-IDR interactions have been proposed to organize these transactivation reactions, forming dynamic clusters, hubs, or condensates that recruit coactivator complexes and Pol II (*44*–*52*). While the physical nature of these transcriptional assemblies and the chemical interactions that drive their formation are debated, the propensity of a TF IDR to form condensates is often correlated with its transactivation capacity (*53*).

Given the abundance of predicted disorder in the NFAT5 sequence (Fig. 4A), we evaluated its propensity to form condensates in cells (*54*, *55*). As controls, we used the four other NFAT proteins (NFATc1-4), which are shorter than NFAT5 but also have predicted IDRs (fig. S5B). Under isotonic conditions, all five GFP-tagged NFAT proteins were homogeneously distributed in the cytoplasm when transiently expressed in human embryonic kidney (HEK) 293T cells (fig. S5C). However, only NFAT5 formed droplet-like condensates when cells were subjected to hypertonic or ionic stress (fig. S5C). Condensates were seen in both live and fixed cells (Fig. 4B and fig. S5, D and E) and were independent of the tag attached to NFAT5 (fig. S5F). Condensate formation was rapid (Fig. 4B) and reversible (Fig. 4C and movie S1) when cells were returned to isotonic media. Fluorescence recovery after photobleaching (FRAP) revealed that these NFAT5 condensates were partially dynamic (Fig. 4D).

Isotonic shrinkage of cells (fig. S4C), which increases macromolecular crowding without changing ionic strength, failed to induce the formation of NFAT5 condensates (fig. S5G), just as it failed to induce NFAT5 activation (fig. S4G). Conversely, increasing intracellular ionic strength without changing cell volume by using nystatin (fig. S5H) or adding NH_4_OAc (fig. S5D) induced the formation of NFAT5 condensates. Thus, NFAT5 condensation, like NFAT5 transactivation (Fig. 3), was driven by increased cytoplasmic ionic strength, rather than by an increase in macromolecular crowding. While many TFs have been shown to form biomolecular condensates, NFAT5 is unique in that it forms condensates only in response to ionic stress: IDRs from nine other transcriptional regulators that form condensates in various contexts were unaffected by ionic or hypertonic stress (fig. S6A) (*47*, *56*). This distinctive ionic stress sensitivity is an evolutionarily conserved property of the NFAT5 proteins, since it was retained when the CTD of human NFAT5 was replaced with that from bird, fish, amphibian, and even invertebrate NFAT5 homologs (fig. S6B).

Condensate formation was not a consequence of overexpressed NFAT5 or the cytoplasmic localization of NFAT5 observed in HEK293T cells (Fig. 4B). Full-length NFAT5 or mini-NFAT5, the latter constitutively localized in the nucleus, both formed nuclear condensates when stably expressed at low levels from a single genomic locus in *Nfat5*^−/−^ IMCD3 cells (Fig. 4, E and F, and movie S2). To examine the behavior of endogenous NFAT5 expressed from its native promoter, we introduced an in-frame mNeonGreen (mNG) tag at the N-terminus of both *Nfat5* alleles in IMCD3 cells (fig. S6, C and D). Live cell imaging revealed that endogenous mNG-NFAT5 accumulated in the nucleus, formed dynamic droplets, and activated target genes in response to ionic and hypertonic stress (Fig. 4, G to J; fig. S6E; and movie S3).

### A prion-like domain mediates ionic strength sensing by NFAT5

**Fig. 5.**
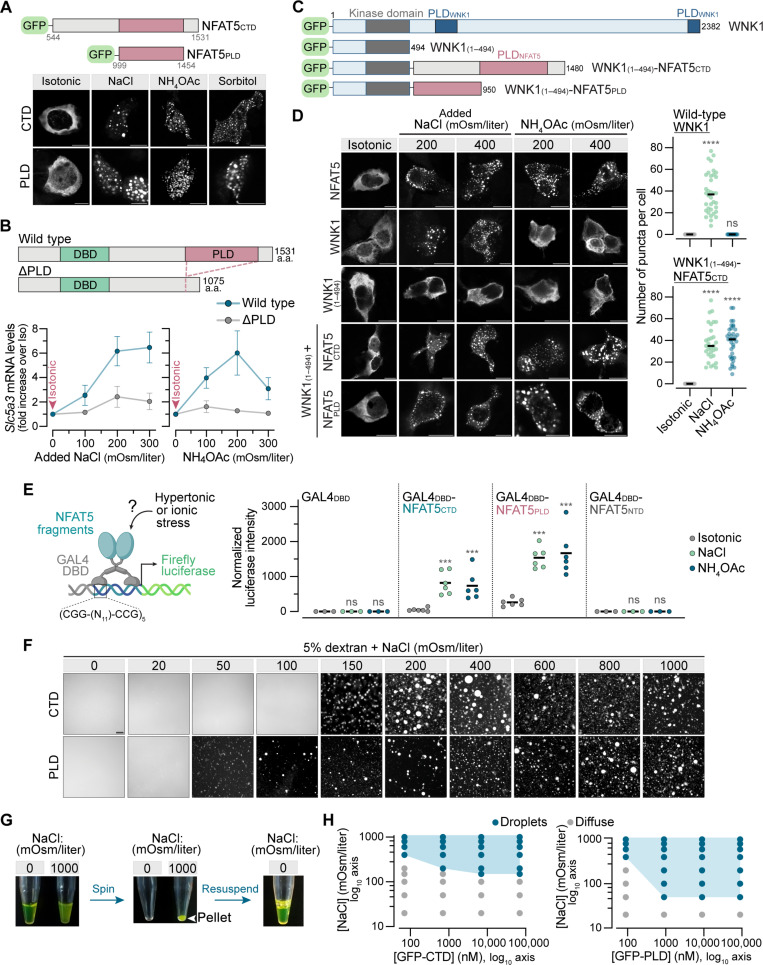
The PLD of NFAT5 is a sensor of solution ionic strength. (**A**) Distribution of GFP-tagged NFAT CTD (Fig. 2A) or PLD (Fig. 4A) in HEK293T cells exposed to various solutes (+100 mOsm/liter, 30 min). See fig. S7A. (**B**) Expression of an NFAT5 target gene (mean ± SD, *n = 3*) in *Nfat5*^−/−^ IMCD3 cells stably expressing NFAT5 variants. See fig. S7B. (**C** and **D**) Domain structure and cellular distribution of GFP-tagged WNK1 and WNK1-NFAT5 chimera proteins. The WNK1 IDR [amino acids (a.a.) 495 to 2382], a sensor of macromolecular crowding, was replaced with the CTD or the PLD of NFAT5. Graphs (D) show the number of puncta per cell (*n* > 20 cells with median indicated). Kruskal-Wallis with Dunn’s multiple comparisons test, ****P < 0.0001. (**E**) Synthetic TFs (left) constructed from the DBD of GAL4 fused to the NFAT5 CTD, PLD or NTD (Figs. 2A and 4A) were tested for their abilities to activate a firefly luciferase reporter (*n* > 3, mean indicated) driven by GAL4 binding sites. Two-way ANOVA test and Sidak’s multiple comparisons test, ***P <0.001. (**F**) Fluorescence microscopy was used to assess condensate formation in vitro by purified (fig. S7C) GFP-CTD (70 μM, top row) and GFP-PLD (90 μM, bottom row). (**G**) Reversibility of GFP-CTD condensates assessed by a centrifugation and resuspension assay. All solutions contain 5% dextran. (**H**) Phase diagrams for purified GFP-CTD (left) and GFP-PLD (right). The boundary between the shaded and unshaded areas of the graph is taken as the phase boundary; crossing this boundary leads to the abrupt drop of diffuse fluorescence and emergence of droplets. Images were obtained across three replicates per condition. Scale bars, 10 μm [(A) and (D)] and 5 μm (F). See also fig. S7.

Among the five NFAT proteins, NFAT5 is distinguished by the presence of an unusually long predicted prion-like domain (PLD) embedded within its intrinsically disordered CTD (Fig. 4A and fig. S5B). This ~450–amino acid PLD is the fifth longest of the ~240 PLDs found in the human genome (*57*). In other proteins, PLDs can function as sensors of the intracellular physicochemical environment, often switching between soluble and condensed or aggregated states in response to changes in pH, temperature, or CO_2_ (*58*–*61*). Like full-length NFAT5, the isolated CTD or PLD of NFAT5 both formed condensates in response to hypertonic or ionic stress (Fig. 5A and fig. S7A).

Deletion analysis demonstrated that the PLD was required for NFAT5 to activate target genes in response to ionic stress (Fig. 5B and fig. S7B). To test sufficiency, we asked whether the PLD of NFAT5 could endow a heterologous protein with sensitivity to ionic stress. The NFAT5 CTD or PLD conferred ionic strength sensitivity when transplanted into WNK1, a kinase that normally forms condensates only in response to crowding stress but not ionic stress (Fig. 5, C and D) (*9*). Second, inspired by previous studies, we constructed synthetic TFs by fusing the DBD of the *S. cerevisiae* galactose-responsive transcription factor (GAL4) protein to the NFAT5 CTD or PLD (Fig. 5E) (*30*, *62*). Both the GAL4_DBD_^_^NFAT5_CTD_ and GAL4_DBD_^_^NFAT5_PLD_ chimeras activated a luciferase reporter driven by a minimal promoter containing GAL4 binding sites (Fig. 5E). Thus, the NFAT5 PLD is sufficient to form condensates and activate transcription of target genes in response to ionic stress. These results again show critical regulation of the transactivation step since the GAL4 DBD is targeted to the nucleus and should constitutively bind to the reporter construct.

To test whether NFAT5 can directly sense ionic strength without other cellular factors, we expressed GFP fusions of its CTD and PLD domains in *Escherichia coli* and purified each protein in a low ionic strength buffered solution (20 mM Na-Hepes) for in vitro phase separation assays (fig. S7C). In the absence of any added salt, 5% dextran, a polysaccharide commonly used to mimic the crowded milieu of the cytoplasm in such assays, failed to induce phase separation (Fig. 5F). However, gradually elevating ionic strength abruptly triggered phase separation of both GFP-CTD and GFP-PLD across a narrow window (~50 mOsm/liter) of NaCl concentrations: The diffuse, homogeneous GFP fluorescence coalesced into spherical droplets, concomitantly reducing background fluorescence (Fig. 5F). The sharp transition between diffuse and condensed GFP-CTD and GFP-PLD supports the crossing of a phase boundary rather than titration of an ion-binding site in the protein. The GFP-CTD condensates could be isolated by centrifugation and readily dissolved when resuspended in a low ionic strength buffer, indicating that they did not form irreversible aggregates (Fig. 5G).

Several controls were done to ensure the specificity of this phenomenon. First, phase separation required the presence of dextran as a crowding agent: No droplets were observed without dextran at NaCl concentrations as high as 1000 mOsm/liter (fig. S7D). Second, GFP alone (fig. S7E) or a GFP-CTD protein lacking the PLD (fig. S7F) did not form droplets. Third, CTD and PLD proteins lacking the GFP tag also formed droplets as assessed by brightfield microscopy (fig. S7G). Last, phase separation did not specifically require NaCl but could be triggered by increasing the ionic strength of the solution using a variety of different salts (fig. S7, H and I).

Phase diagrams of GFP-CTD and GFP-PLD were constructed by varying the ionic strength at protein concentrations ranging between ~100 nM and ~100 μM (Fig. 5H). At the lowest protein concentrations tested (~70 to 100 nM), phase separation was observed only at NaCl concentrations above ~300 mOsm/liter. Thus, the purified NFAT5 PLD, even at nanomolar concentrations in vitro, phase separates in response to NaCl concentrations that would impose hypertonic stress on cells.

### NFAT5 condensation propensity correlates with its transactivation capacity

The chemical nature of the homotypic and heterotypic interactions that drive the assembly of transcriptional condensates is an active area of investigation. Both interactions involving amino acid side chains and the amide and carbonyl groups on the protein backbone have been implicated (*63*, *64*).

**Fig. 6.**
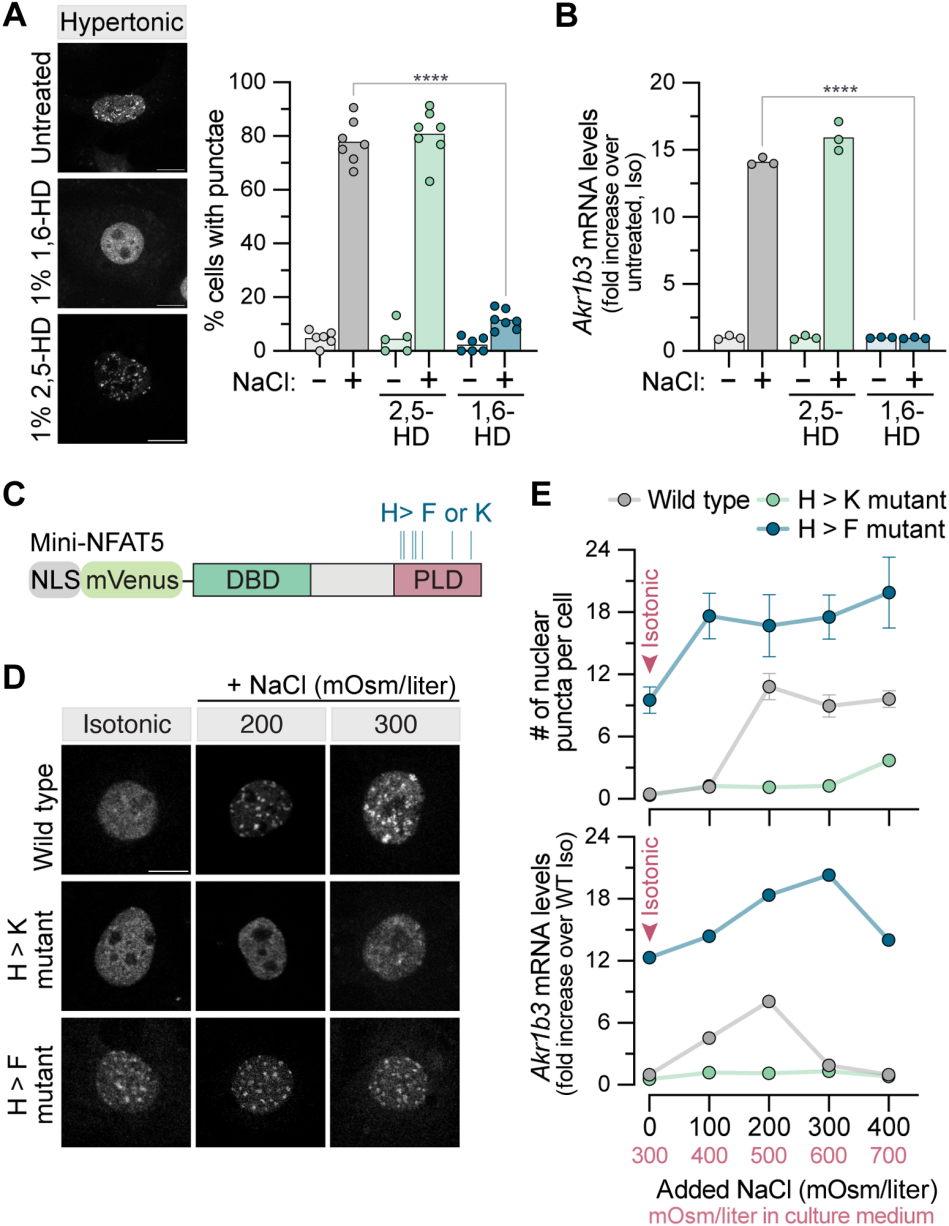
NFAT5 activity correlates with phase separation propensity. (**A**) Distribution of GFP-NFAT5 (left) stably expressed in *Nfat5*^−/−^ IMCD3 cells exposed to hypertonic stress [NaCl (+200 mOsm/liter)] in the presence of 1% 1,6-hexanediol (1,6-HD) or 2,5-hexanediol (2,5-HD). The graph on the right shows the percentage of cells with nuclear puncta (>100 cells per data point), with each bar depicting the mean of six to seven independent measurements. (**B**) Expression of the NFAT5 target gene *Akr1b3* in response to a 10-hour treatment of 1% 1,6-HD or 2,5-HD in isotonic or hypertonic [NaCl (+200 mOsm/liter)] media. Bars denote the mean of four independent experiments shown as points. (**C**) Vertical blue lines mark the position of the seven histidines within the PLD targeted for mutagenesis to phenylalanine (F) or lysine (K) in mini-NFAT5. (**D**) Distribution of mVenus fluorescence in the nucleus of *Nfat5*^−/−^ cells stably expressing the indicated variants of mini-NFAT5 after 30 min in isotonic or hypertonic [NaCl (+200 or +300 mOsm/liter)] media. (**E**) Number of mini-NFAT5 nuclear puncta per cell (top) and target gene expression (bottom) in IMCD3 cells treated for 8 hours with increasing concentrations of NaCl. Each point shows the mean ± SD of three independent experiments (*n* > 30 cells each). Scale bars, 10 μm [(A) and (D)]. Statistics: Statistical significance was determined by a two-way ANOVA test, Sidak’s multiple comparisons. ****P < 0.0001. See also figs. S8 and S9.

We sought to perturb the chemical interactions that drive NFAT5 condensation using three methods. Aliphatic alcohols are commonly used to disrupt IDR-mediated interactions (*65*). Recent solution nuclear magnetic resonance studies discovered that 1,6-hexanediol, but not its structural isomer 2,5-hexanediol, interacts with the carbonyl groups of polypeptide backbone more avidly than amino acid side chains, thereby potentially disrupting labile cross-β interactions (*63*, *66*, *67*). Treatment of cells with 1,6-hexanediol inhibited the formation of NFAT5 condensates (Fig. 6A) and the transcription of NFAT5 target genes (Fig. 6B and fig. S8A); 2,5-hexanediol had no effect.

We next sought to disrupt condensate formation by altering the amino acid sequence of NFAT5. Compared to the human proteome, NFAT5 is enriched in glutamine (Q) and serine (S) but depleted in the charged residues glutamic acid (E), aspartic acid (D), lysine (K), and arginine (R) (fig. S8, B and C). Since glutamine residues are known to be enriched in PLDs and can stabilize cross-β interactions by forming polar zippers (*68*), we mutated all 109 glutamine residues in the NFAT5 PLD to alanine (fig. S8B). This variant (NFAT5_QA) was expressed at levels comparable to wild-type (WT) NFAT5 and was properly distributed in both the nucleus and cytoplasm under isotonic conditions (fig. S8, D and E); however, NFAT5_QA failed to form condensates or activate target genes in response to hypertonic stress (fig. S8, E to G). NFAT5_QA also failed to accumulate in the nucleus in response to hypertonic stress (fig. S8F), suggesting that nuclear condensate formation may be required for nuclear accumulation. Changing all 69 of the serine residues to alanine in the PLD had no effect on NFAT5 activity (fig. S8, D and H to J).

The NFAT5_QA variant is compositionally very different from WT NFAT5, having lost its prion-like character. Therefore, we sought to make more subtle mutations that would tune NFAT5 self-associative behavior, using in vitro phase separation assays as a guide. We focused on histidine residues because the p*K*_a_ (where *K*_a_ is the acid dissociation constant) of its imidazole side chain is known to be sensitive to the ionic strength of the solution (*12*, *69*). We mutated seven histidine residues conserved between mammals and birds within the NFAT5 PLD to lysines (NFAT5_HK) or phenylalanines (NFAT5_HF), mutations designed to weaken or strengthen, respectively, side chain interactions in condensates (fig. S9A) (*64*). In purified phase separation assays, GFP-CTD_HK had a lower propensity to phase separate, requiring higher NaCl concentrations to form droplets at all protein concentrations tested (fig. S9, B and C). In contrast, GFP-CTD_HF formed tangled precipitates of irregular morphology, in contrast to the mostly spherical droplets observed with GFP-CTD_HK or GFP-CTD (fig. S9B). Full-length NFAT5_HF also formed aggregates when stably expressed in cells, based on condensate morphology (fig. S9D) and lack of FRAP (fig. S9E). We presume that enhanced self-associative interactions caused by the H > F mutations resulted in the formation of solid aggregates rather than dynamic condensates. Practically, however, aggregate formation precluded a comparison of transcriptional activity between the variants.

To assess the impact of increasing or decreasing associative interactions on transactivation, we introduced the H > K and H > F mutations into mini-NFAT5 and stably expressed these variants in *Nfat5*^−/−^ cells (Fig. 6C and fig. S9F). We reasoned that mini-NFAT5 would be less prone to aggregation since it has a much shorter IDR compared to full-length NFAT5 (Fig. 2A). Compared to the parent mini-NFAT5 protein, the HK and HF variants had a lower and higher propensity, respectively, to form condensates in response to hypertonic stress (Fig. 6, D and E). This ranking of condensation propensity (HF > WT > HK) was consistent with the predictions of in vitro phase diagrams of the respective GFP-CTD variants (fig. S9C) and mirrored their transactivation capacity (Fig. 6E): Mini-NFAT5_HF formed condensates and activated target genes even under isotonic conditions, while mini-NFAT5_HK was much less active even at high levels of hypertonicity (700 mOsm/liter) (Fig. 6E).

### Ionic stress triggered recruitment of transcriptional coactivators by the NFAT5 PLD

To test whether NFAT5 condensation was sufficient to activate target genes, we used an established optogenetic system based on fusion of IDRs to Cry2 (fig. S10A) (*70*). Cry2 undergoes self-association upon exposure to blue light, increasing the local concentration of the appended NFAT5 IDR and inducing condensate formation (fig. S10, B and C, and movies S4 to S6). Cry2–nuclear NFAT5 activated target genes in response to hypertonic stress, showing that it was properly regulated (fig. S10D). However, light-induced condensation of this fusion was not sufficient to activate transcription under isotonic conditions. On the contrary, illumination inhibited the transcriptional response, as has been reported for some other TFs (*53*), perhaps by sequestering NFAT5 into inactive condensates.

**Fig. 7.**
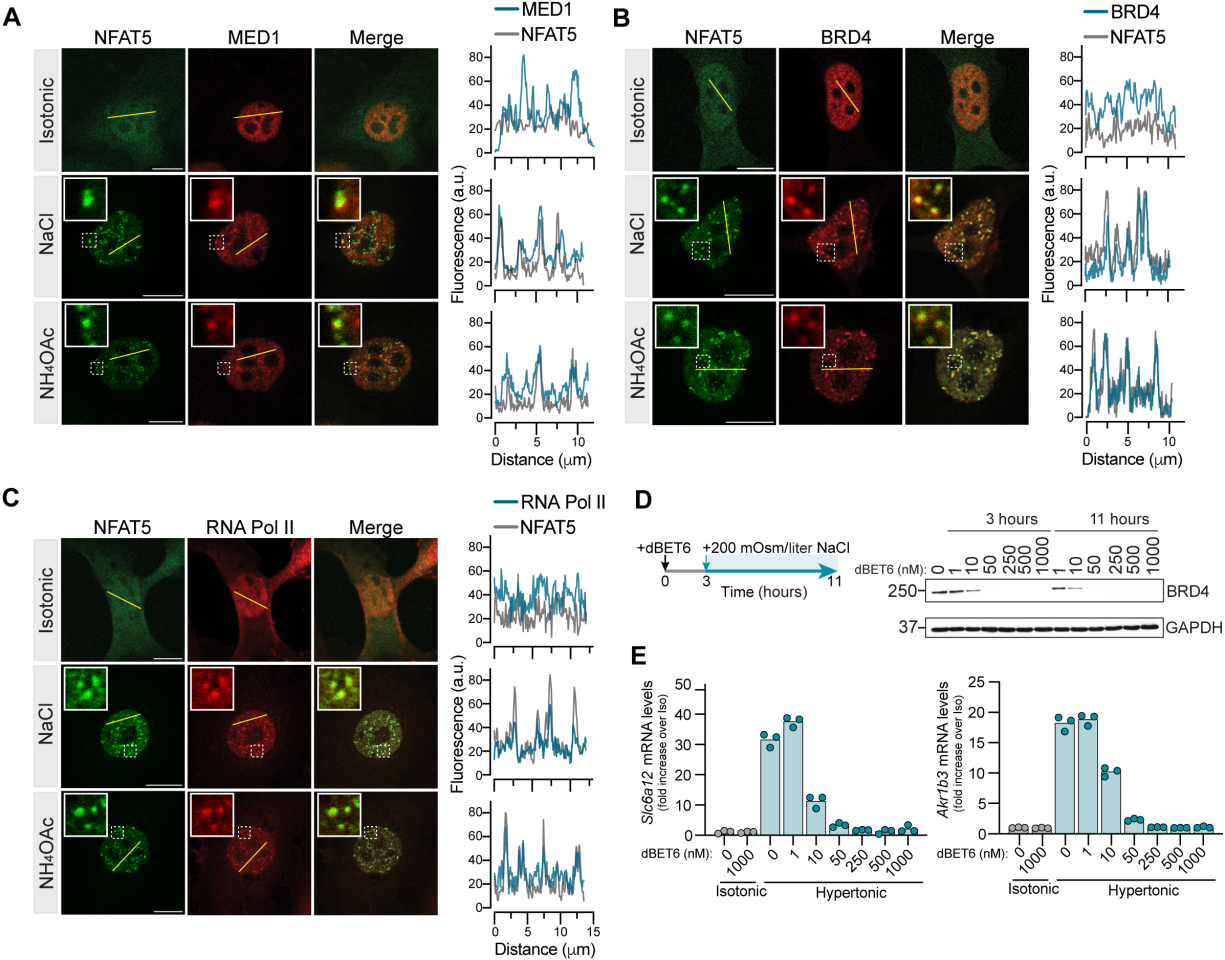
NFAT5 condensates recruit transcriptional coactivators. (**A** to **C**) Recruitment of MED1 (A), BRD4 (B), and Pol II (C) in stress-induced nuclear NFAT5 condensates in *Nfat5*^−/−^ IMCD3 cells stably expressing GFP-NFAT5 after the addition of NaCl or NH_4_OAc (+200 mOsm/liter, 30 min). Line scans show fluorescence intensity traces for NFAT5 (gray) and the second protein (dark teal) along the trajectories of the yellow line in the images. Overlapping peaks in these traces indicate colocalized puncta, which are also highlighted in the zoomed insets. (**D**) Cells were treated (left diagram) with increasing concentrations of dBET6 to acutely induce BRD4 degradation (assessed by the immunoblot on the right). (**E**) Impact of dBET6 degradation [as shown in (D)] on induction of two NFAT5 target genes (*Slc6a12* and *Akr1b3*) in response to hypertonic stress [NaCl (+200 mOsm/liter)] for 11 hours. Bars denote the mean of three measurements, and the experiment was repeated three times. Scale bars, 10 μm [(A) to (C)]. See also figs. S12 and S13.

Since self-association per se was not sufficient to activate NFAT5, we considered the hypothesis that hypertonic and ionic stress promotes the heterotypic interaction between NFAT5 and other nuclear proteins. A quantitative proteomic strategy based on multiplexed tandem mass tags (TMT) was implemented to identify mini-NFAT5 interacting nuclear proteins using proximity biotinylation in two different cell lines (fig. S11, A to C) (*71*). Gene set enrichment analysis (GSEA) of mini-NFAT5 proximal proteins enriched in hypertonic over isotonic conditions revealed a strong signature of transcriptional coactivators. Among all Gene Ontology (GO) categories, the GO molecular function gene set “transcription coactivator activity” had the highest normalized enrichment score (fig. S11, D and E). Other enriched gene sets included proteins that positively regulate transcription, splicing and histone acetylation, as well as multiple subunits of the Pol II holoenzyme (fig. S11, C and D). Given the critical role of condensates in NFAT5 activation by hypertonic stress, we focused on bromodomain-containing protein 4 (BRD4) and the Mediator complex, known to be components of coactivator condensates that activate transcription by Pol II (*46*, *47*). Stress-induced NFAT5 condensates in cells recruited mediator of RNA polymerase II transcription subunit 1 (MED1), BRD4, and Pol II (Fig. 7, A to C), consistent with the proximity biotinylation data (fig. S11C). Acute depletion of BRD4 using the PROTAC dBET6 (Fig. 7, D and E, and fig. S11F) or occlusion of its acetyl-lysine binding site using the small-molecule inhibitor JQ1 (*72*) (fig. 11G) impaired the transcriptional response to hypertonic stress.

To test whether the CTD of NFAT5 was sufficient to recruit coactivators to an ectopic genomic locus, we used a Tet Operator (TetO) array assay (*53*, *73*, *74*). The NFAT5 CTD and (as a control) the AD of the constitutive transcriptional activator VP16 were tethered to a synthetic array of ~20,000 Tet repressor (TetR) binding sites integrated into a single genomic locus in U2OS cells (fig. S12A) and visualized as a single focus by fluorescence microscopy (fig. S12B). Both fusions recruited endogenous MED1 and BRD4 to the TetO array under isotonic conditions (fig. S12, B to E). However, hypertonic stress significantly increased MED1 and BRD4 recruitment by the NFAT5 CTD but had no effect on the VP16 AD. Within the CTD, the PLD of NFAT5 was both necessary and sufficient to recruit MED1 and BRD4. The PLD alone tethered to the TetO array (fig. S13A) recruited MED1 and BRD4 (fig. S13, B to E); deletion of the PLD from the CTD abolished recruitment (fig. S13, F and G). We also assessed transcription from the TetO array using the in-built cyan fluorescent protein reporter (*73*). Hypertonic stress increased reporter transcription by NFAT5 CTD and PLD but had no effect on transactivation by the VP16 AD (fig. S13H).

**Fig. 8.**
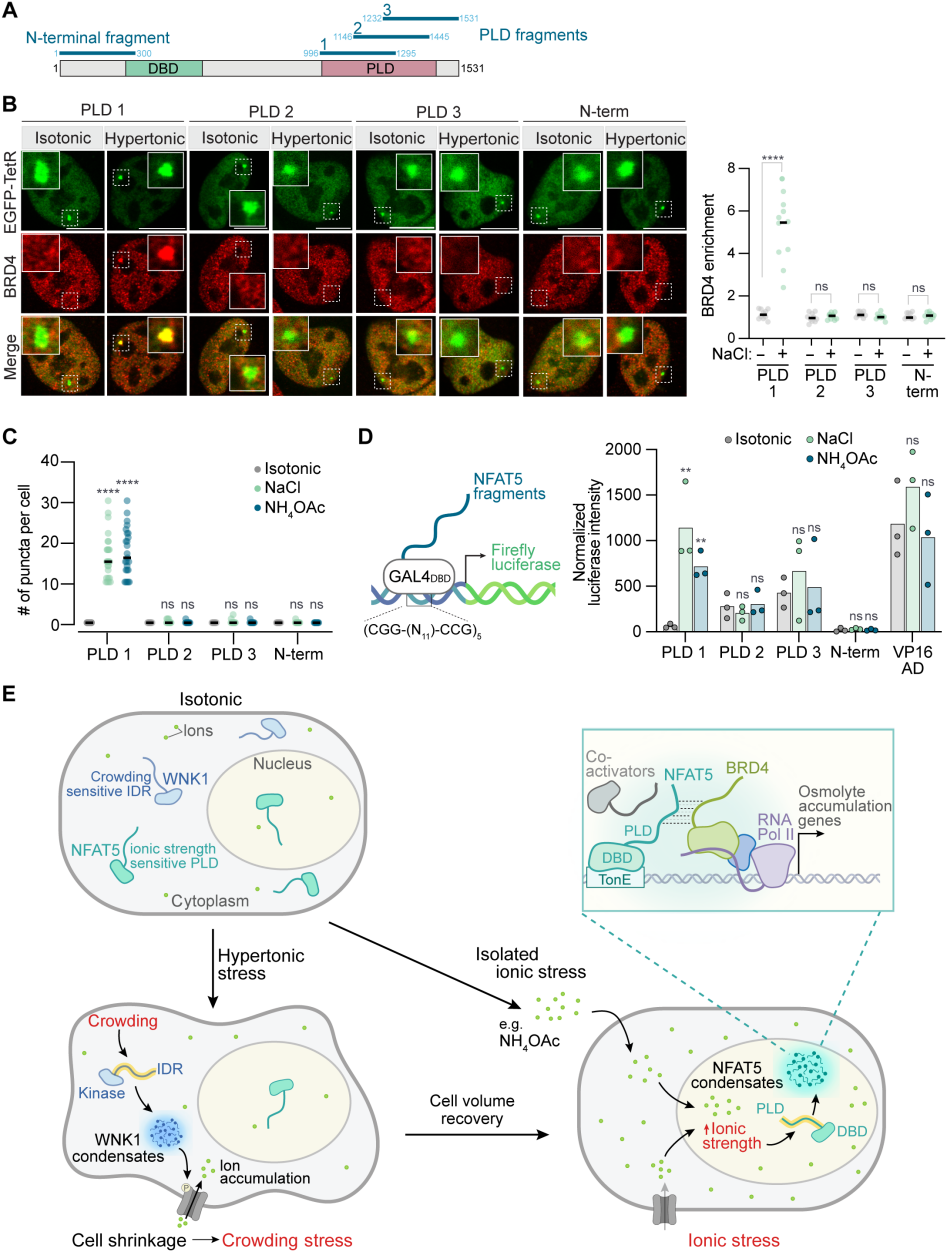
Ionic stress response regulation by the NFAT5 PLD. (**A**) Position of the four 300–amino acid fragments of NFAT5 tested in (B) to (D). (**B**) Recruitment of endogenous BRD4 (red) to a TetO array in U2OS cells by EGFP-TetR DBD (green) fused to the four fragments of NFAT5 (see fig. S12A). Insets show a magnified view of the TetO array, visualized as a single dot of EGFP fluorescence. Enrichment of BRD4 in the EGFP-marked TetO array is plotted on the right for individual cells, with the mean indicated. Scale bars, 10 μm. (**C**) Condensate formation by hemagglutinin-tagged NFAT5 fragments in HEK293T cells (*n* > 25, median indicated). (**D**) Transactivation capacity of NFAT5 fragments (*n* = 3, bars show mean) or the VP16 AD (as a control) using the reporter assay shown in Fig. 5E. (**E**) A model for hypertonic and ionic stress adaptation. The IDR in WNK1 and PLD in NFAT5 each sense specific chemical properties of the intracellular environment. In response to hypertonic stress, the rapid loss of cell volume leads to an increase in macromolecular crowding, which activates the crowding sensor kinase WNK1 (but not NFAT5) (*9*). Through a kinase cascade, WNK1 activates transporters that increase intracellular ion concentrations, allowing cytoplasmic rehydration and volume recovery at the expense of elevated ionic strength. If persistent, this increase in ionic strength is the trigger for NFAT5 activation, leading to a transcriptional response that exchanges these ions for osmolytes. We speculate that NFAT5 has evolved to sense and facilitate adaptation to diverse ionic stressors (even those, like NH_4_OAc, that do not cause hypertonic stress). Statistics: Statistical significance was determined by a Kruskal-Wallis test, Dunn’s multiple comparisons [(B) and (C)], or a two-way ANOVA with Sidak’s multiple comparisons test (D). ****P < 0.0001 and **P < 0.01. See also figs. S12 and S13.

Further analysis identified a 300–amino acid segment within the PLD that was sufficient to recruit BRD4, form condensates, and activate transcription of a reporter in response to hypertonic stress (Fig. 8, A to D). Recruitment of BRD4 to the TetO array by this fragment of the PLD was observed only under hypertonic stress and not under isotonic conditions (Fig. 8B). Together, our data are consistent with the model that the NFAT5 PLD directly responds to increasing intracellular ionic strength, driving the formation of transcriptional assemblies containing NFAT5 and coactivator proteins to activate Pol II transcription at target genes.

## DISCUSSION

Comparative physiologists have made the unexpected observation that cytoplasmic ionic strength is conserved between organisms from all branches of life that live in markedly diverse habitats (*1*, *12*). A revealing exception is provided by certain halophilic bacteria that have high molar concentrations of cytoplasmic ions. The proteomes of these halobacteria have undergone extensive sequence changes during evolution to function in this extreme environment, but the consequence of this adaptive strategy is that they can only live in highly hyperosmotic habitats (*75*). For most other organisms, the preferred strategy has been the evolution of osmoregulatory systems that allow the maintenance of a stable intracellular ionic milieu despite variable extracellular osmolarity (*1*). This stable cytoplasmic ionic content allows protein function to be maintained in environments with fluctuating osmolarities without adaptive changes in protein sequence.

Our work resolves a longstanding mystery around the signaling mechanisms that detect and mitigate elevated ionic strength in animal cells. NFAT5 was originally cloned as a sequence specific TF that activates the transcription of genes induced by hypertonic stress; these genes have an upstream binding site (TonE) recognized by NFAT5 (*18*, *19*). Because hypertonic stress leads to two distinct changes, decreased cell volume and increased intracellular ionic strength, both the chemical nature of the signal and how this signal is communicated to NFAT5 have remained unclear. We find that NFAT5 is selectively activated by elevated ionic strength and not by cell volume changes. These results, when integrated with recent work on WNK1 (*9*), allow us to propose a comprehensive model for cellular adaptation to hypertonic stress (Fig. 8E).

In our view, the most unexpected conclusion of our work is the identification of a single protein, NFAT5, as both a sensor and effector for ionic stress responses in animal cells. NFAT5 contains a PLD that directly senses the increase in intracellular ionic strength and recruits coactivators like the Mediator complex and BRD4 to activate an adaptive transcriptional response. This system is starkly different from the analogous HOG pathway in fungi, a traditional signaling system with membrane sensors, kinase transducers, and transcriptional effectors (*16*). Reduced cytoplasmic ionic strength in animals, often caused by hypotonic stress and cell swelling, is sensed and ameliorated by a nontranscriptional mechanism—changes in ion fluxes through the volume regulated anion channel (*6*). We speculate that the function of NFAT5 is not restricted to hypertonic stress responses, but rather NFAT5 is tasked with maintaining intracellular (and intranuclear) ionic strength in the optimal window required for the function of cellular processes.

Our work raises several questions for future research. There are likely to be other mechanisms and pathways that regulate the activity of NFAT5, including posttranslational modifications. One mechanism not explored in our work is the role of condensate formation in trafficking of NFAT5 between the cytoplasm and the nucleus, known to be sensitive to ambient tonicity (*29*, *76*). How ionic strength increases productive homo- and heterotypic interactions with the NFAT5 PLD to activate transcription is also unknown. Both backbone and side-chain interactions may contribute, consistent with the propensity of PLDs to form cross-β interactions. Our mutagenesis experiments point to the importance of histidine residues. The unique p*K*_a_ of histidine, positioned close to the intracellular pH, may allow it to regulate the conformational ensemble of the PLD and hence its propensity to form cross-β interactions (*12*, *63*). We hope that ionic stress–activated transcription emerges as a useful system to investigate these important questions in the context of NFAT5 and other environmentally responsive transcriptional programs.

## MATERIALS AND METHODS

### Mammalian cell culture and stress treatments

IMCD3 Flp-In cells were cultured in Dulbecco’s modified Eagle’s medium (DMEM)/F12 (HyClone DMEM with l-glutamine and Hepes; Cytiva) supplemented with 10% fetal bovine serum (FBS; Atlanta Biologicals), and penicillin (40 U/ml), and streptomycin (40 μg/ml) (Gemini Biosciences). HEK293T, HEK293FT, and U2OS cells were cultured in DMEM (Cytiva) containing high glucose (Thermo Fisher Scientific) and supplemented with 10% FBS, 1 mM sodium pyruvate (Gibco), 2 mM l-glutamine (Gemino Biosciences), 1× MEM nonessential amino acid solution (Gibco), penicillin (40 U/ml), and streptomycin (40 μg/ml). HAP1 cells were cultured in IMDM [Iscove’s modified Dulbecco’s medium supplemented with 10% FBS, 2 mM l-glutamine, penicillin (40 U/ml), and streptomycin (40 μg/ml)]. HAP1 cells were maintained in media containing deacetyl-baccatin-III (2.5 μM; Selleckchem) to prevent conversion to diploidy (*77*). All cell lines were kept in a humidified atmosphere containing 5% CO_2_ at 37°C. To impose hypertonic or ionic stress, the indicated amounts of NaCl, sorbitol, urea, and NH_4_OAc were added to growth media (which has a baseline osmolarity of ~300 mOsm/liter). Thus, the total media osmolarity was ~300 mOsm/liter + added osmolarity of NaCl, sorbitol, urea, or NH_4_OAc.

### Mammalian cell lines

Human near haploid cells (HAP1) have been described in our prior publications (*28*) and were validated before our screens using propidium iodide staining to ensure they have a haploid DNA content. Human HEK293T and HEK293FT cells were obtained from commercial vendors with certificates of authentication and used at low-medium passage (<20) without additional short tandem repeat (STR) profiling. Mouse IMCD3 Flp-In (*78*) cells were engineered to contain a flippase recombination target (FRT) site at a unique site in the genome using the Flp-In system from Thermo Fisher Scientific, allowing for Flp-mediated stable gene expression from a single, defined genomic locus to readily create sets of isogenic cell lines. IMCD3 Flp-In cells were validated by ensuring they contain a *lacZeo* cassette at the integration locus as recommended by the manufacturer. Cell lines were confirmed to be mycoplasma-negative when cultures were started in the lab and when there was a change in the growth rate or morphology of the cell lines.

#### IMCD3 Flp-In cells and derivatives

The *Nfat5^−/−^* clonal IMCD3 Flp-In cell line (used for the stable, single-locus expression of all NFAT5 variants described in this paper) was generated using CRISPR-Cas9 with a sgRNA targeting the DBD (Rel-homology domain) of *Nfat5* (data S2). The sgRNA was cloned into pSpCas9(BB)-2A-mCherry (*27*) and transiently transfected into WT IMCD3 Flp-In cells using X-tremeGene 9 DNA transfection reagent (Roche). Two days posttransfection, mCherry-positive single cells were sorted and assessed by immunoblotting (to measure NFAT5 protein abundance, transcriptional response to hypertonic stress, and sequencing) (Fig. 1C and fig. S1A). Clonal *Akap13*^−/−^ (fig. S2E) and *Wnk1^−/−^* IMCD3 Flp-In cell lines (Fig. 3F and fig. S4, H and N) were generated using a two-guide system, cotransfecting pSpCas9(BB)-2A-GFP (Addgene plasmid #48138) and pSpCas9(BB)-2A-mCherry expressing gene-specific sgRNAs along with Cas9 and either GFP or mCherry. Cells expressing both fluorescent markers were single-cell–sorted using a Sony SH800S Cell Sorter, and edited clones were assessed for genetic knockout via genomic polymerase chain reaction (PCR; to ensure excision of the DNA fragment between the two Cas9 cut sites). For the *Wnk1^−/−^* clonal line, genetic knockout was also confirmed by immunoblotting to measure WNK1 protein abundance (data S2).

All NFAT5 variants described in this study were assessed after stable expression at moderate levels from the same single genomic locus in an otherwise isogenic background lacking WT NFAT5 (to prevent the potentially confounding effects of the presence of both WT and mutant NFAT5 in the same cell). To accomplish this goal, all variants were stably expressed in a clonal *Nfat5^−/−^* IMCD3 Flp-In cell line using site-specific recombination into a single site in the genome using the Flp-In system. Briefly, cells at 40 to 60% confluency in six-well plates were transfected with a mixture of 2.7 μg of pOG44 Flp-Recombinase Expression Vector (Invitrogen), 0.3 μg of expression pEF5-FRT-V5_DEST plasmid (Invitrogen) carrying the gene of interest, and XtremeGene 9 (Roche) in 200 μl of Opti-MEM Reduced Serum Medium (Gibco). Positive clones were selected for with the addition of hygromycin B (200 μg/ml; VWR Life Science) to the growth medium. Expression of NFAT5 variants was confirmed by immunoblotting to measure the abundance of the protein encoded by the transgene (fig. S3A).

To generate a transcriptional reporter for NFAT5 activity (8xTonE-GFP), we replaced the seven TCF/LEF binding sites in a previously described reporter (*28*) for WNT signaling with eight concatenated copies of the NFAT5-binding TonE site (5′-TGGAAAATTAC-3′). The final 8xTonE-GFP reporter contains eight TonE sites, a minimal promoter, a gene encoding enhanced green fluorescent protein (EGFP), and the 5′ untranslated region of the SuperTOPflash WNT reporter. The full 8xTonE-GFP cassette was introduced into the unique Flp-In locus of IMCD3 Flp-In cells using site-specific recombination. These reporter cells were subsequently modified for stable expression of an inducible Cas9 (iCas9) for the genome-wide CRISPR screen shown (Fig. 1G and fig. S2B). A clonal cell line for the screen was established on the basis of the abundance of nuclear Cas9 protein.

### HAP1 cells stably expressing the 8xTonE-GFP hypertonic stress reporter

HEK293FT cells growing in a 10-cm plate were transfected with pCF567:pLenti-PuroR carrying the 8xTonE-GFP reporter cassette mixed with polyethylenimine (PEI) in Opti-MEM. Virus was harvested and then concentrated by ultracentrifugation in sterile 25 mm–by–89 mm Beckman centrifuge tubes for 1.5 hours at 65,000*g* at 4°C using a Beckman SW 32 Ti rotor. Pelleted virus was resuspended in 10 ml of IMDM (see below for media compositions). Five milliliters of resuspended virus was mixed with 5 ml of IMDM and polybrene (MilliporeSigma) at a final concentration of 4 μg/ml and added to HAP1 cells. Forty-eight hours after transduction, HAP1 cells were treated with puromycin (1 μg/ml; MilliporeSigma) to select for cells with a stably integrated 8xTonE-GFP cassette. The resulting puromycin-resistant polyclonal population was used to create the gene trap (GT) mutant library for genetic screening.

### Yeast culture and treatments for reporter assays

All yeast cells were cultured with shaking at 30°C. For RNA extraction for HOG pathway analysis, W303a (*MATa leu2*-*3*,*112 trp1*-*1 can1*-*100*, *ura3*-*1*, *ade2*-*1*, and *his3*-*11*,*15*) cells were cultured in yeast extract, peptone, and dextrose medium supplemented with 0.004% adenine or complete synthetic medium (CSM; Sunrise Science Products) supplemented with 2% glucose and 0.004% adenine. For reporter assays and Western blot analysis requiring galactose induction (Fig. 2D), saturated overnight cultures of cells in CSM + 2% raffinose, 0.1% glucose, and 0.004% adenine were diluted to an OD_600_ (optical density at 600 nm) of 0.3 (3.0 × 10^6^ cells per milliliter of media) the next morning in CSM + 2% raffinose and 0.004% adenine lacking glucose. Early log-phase cells were then cultured in CSM supplemented with 2% galactose and 0.004% adenine for 2 to 4 hours before the application of hypertonic stress as described in the figures. 8xTonE-*pCYC1*-GFP fluorescence was measured after 4 hours by a BD Accuri C6 Flow Cytometer using a 473-nm laser for excitation and a 520/30-nm bandpass filter to collect emitted light.

### Western blotting and immunoprecipitation

IMCD3 cells were washed and scraped with ice-cold 1× phosphate-buffered saline (PBS). Cell pellets for Western blotting were lysed in radioimmunoprecipitation assay (RIPA) buffer [50 mM tris-HCl (pH 7.4), 100 mM sodium chloride, 1% NP-40, 0.5% sodium deoxycholate, 0.1% SDS, 0.5 mM dithiothreitol (DTT), 1× SigmaFast protease inhibitor cocktail from Milliore-Sigma, and 1× PhosSTOP phosphatase inhibitor cocktail from Roche]. Lysates were cleared by centrifugation at 20,000*g* for 30 min, and protein concentrations were determined using the BCA protein Assay (Thermo Fisher Scientific). Lysate volumes containing equal total amounts of protein (by mass) were dissolved in 1× NuPAGE lithium dodecyl sulfate (LDS) sample loading buffer and analyzed by SDS–polyacrylamide gel electrophoresis (SDS-PAGE). The resolved proteins were transferred onto a nitrocellulose membrane using a wet electroblotting system (Bio-Rad) followed by immunoblotting [more details are provided in (*28*)].

To measure protein abundance (e.g., GFP reporter protein; fig. S3G) in W303a yeast cells expressing mini-NFAT5, 3.0 × 10^7^ of log-phase cells growing in CSM supplemented with 2% galactose were collected by centrifugation, washed with 1 ml of ice-cold water, and resuspended in Y-PER Yeast Protein Extraction Reagent (Thermo Fisher Scientific) mixed with 1× SigmaFast protease inhibitor cocktail from MilliporeSigma. Resuspended cells were mixed with acid-washed glass beads (Sigma-Aldrich) and lysed by vortexing. Lysates were then cleared at 20,000*g* for 20 min at 4°C, and equal amounts of protein (by mass) from each sample were analyzed by SDS-PAGE and immunoblotting.

For immunoprecipitation (IP) from W303a cells expressing mini-NFAT5 or its variants (dimerization mutant or DNA binding mutant; Fig. 2G), 5.0 × 10^8^ of log-phase cells growing in CSM supplemented with 2% galactose were collected by centrifugation and washed with 10 ml of ice-cold water. Washed cell pellets were then resuspended in ~1 ml of lysis buffer [20 mM Hepes (pH 7.4), 150 mM potassium acetate, 5% glycerol, 1% NP-40, 1× SigmaFast protease inhibitor cocktail, and 0.5 mM DTT], mixed with acid-washed glass beads, and lysed through a 3-min bead beater cycle at 4°C (MiniBeadBeater-16, Model 607, 3450 RPM, 115 V, BioSpec Products). Samples were spun at 1000*g* for 1 min at 4°C to separate the protein sample from the beads and coarse cell debris and then at 20,000*g* for 20 min at 4°C to obtain a clear lysate for IP. NLS-mRuby3-mini-NFAT5-1D4 variants were immuno-purified via the 1D4 epitope tag by mixing a lysate sample containing 2.5 mg of total protein with anti-1D4 antibody (The University of British Columbia) covalently conjugated to Protein A Dynabeads (Thermo Fisher Scientific, Invitrogen). Following a 4-hour incubation at 4°C, beads were washed three times with a wash buffer [50 mM tris-HCl (pH 7.4), 100 mM sodium chloride, and 1% glycerol]. Proteins captured on the anti-1D4 beads were eluted using 2× NuPage LDS sample buffer (95°C, 10 min). Equal volumes of eluates were analyzed by immunoblotting to measure the abundance of 1D4-tagged NFAT5 variants. The abundance of PGK1 (Invitrogen) was measured in the lysates to ensure that sample inputs for the IPs contained the same amount of total protein.

### Measurement of mRNA abundance by RT-qPCR

RNA was extracted from IMCD3 cells using TRIzol reagent (Life Technologies), and cDNA was synthesized using iScript Reverse Transcription Supermix (Bio-Rad). Real-time reverse transcription quantitative PCR (RT-qPCR) for mouse *Akr1b3* (aldose reductase, NFAT5 target gene), mouse *Slc5a3* (sodium/myo-inositol cotransporter), mouse *Slc6a12* (sodium- and chloride-dependent betaine transporter), and mouse *Gapdh* (housekeeping gene) was performed on a QuantStudio 5 Real-Time PCR System (Thermo Fisher Scientific) with the following primers: *Akr1b3* (forward: 5′-CCTCA-GGGAACGTGATACCT-3′ and reverse: 5′-CAATCAGCTTCTCCT-GAGTT-3′), *Slc5a3* (forward: 5′-GGCAGCAGACATTGCCGTA-3′ and reverse: 5′-AATCGCCACCCAGGTCATAGA-3′), *Slc6a12* (forward: 5′-TCTTGGGCTTCATGTCTCAG-3′ and reverse: 5′-GACCTGA-CTCAGCCACTTCA-3′), and *Gapdh* (forward: 5′-AGTGGCAA-AGTGGAGATT-3′ and reverse: 5′-GTGGAGTCATACTGGAACA-3′). Reporter GFP transcript levels were assessed using the following primers: forward: 5′-GACGTAAACGGCCACAAGTT-3′ and reverse: 5′-GAACTTCAGGGTCAGCTTGC-3′. Transcript levels relative to *Gapdh* were calculated using the ΔΔCt method.

For yeast mRNA measurements, 4.0 × 10^7^ of cells were pelleted, resuspended in TRIzol, and lysed using agitation with acid-washed glass beads (5 min, room temperature). After RNA samples were isolated, samples were treated with deoxyribonuclease (DNase) at 37°C for 1 hour, followed by addition of EDTA and a 10-min incubation at 65°C to inactivate the DNase. cDNA was synthesized using the iScript Reverse Transcription Supermix. RT-qPCR for *STL1* (Sugar Transporter–like Protein, HOG pathway target gene) and *ACT1* (housekeeping gene) was performed with the following primers: *STL1* (forward: 5′-GTTGCGGTATTTCATCAC-3′ and reverse: 5′-CATAGTTGAACTGTTTACC-3′), *ACT1* (forward: 5′-GTGTG-GGGAAGCGGGTAAGC-3′ and reverse: 5′-GTGGCGGGTAAAG-AAGAAAATGGA-3′). Transcript levels relative to *Act1* were calculated using the ΔΔCt method.

### Pooled genome-wide CRISPR-Cas9 screens in IMCD3 cells

Genome-wide CRISPR and haploid screens to identify positive and negative regulators of NFAT5 activity using a fluorescent transcriptional reporter were performed exactly like our previous screens interrogating the Hedgehog (*27*) and WNT (*28*) signaling pathways. CRISPR guide RNA library amplification, lentiviral production, functional titer determination and transduction were performed as previously described (*27*, *79*). To generate a genome-wide mutant cell population for screening, IMCD3 8xTonE-GFP iCas9 cells were transduced with the Brie genome-wide library of sgRNAs (*80*), which targets 19,674 mouse genes with ∼4 sgRNAs each and includes 1000 nontargeting controls. Each of 45 T-225 flasks was seeded with 8.0 × 10^6^ IMCD3 8xTonE-GFP iCas9 cells. After cells recovered for 8 hours, ~2.4 × 10^6^ lentiviral particles mixed with polybrene (2.5 μg/ml) were added to each flask. After the infection was allowed to proceed for 24 hours, successfully transduced cells were selected by supplementing the media with puromycin (2 μg/ml). Screens were scaled so that each guide was represented in at least 1000 cells in the starting mutant library. For each screen, iCas9 was induced for 5 days using doxycycline (1 μg/ml) to initiate genome editing, and then the entire mutant cell population was subjected to hypertonic stress [NaCl (200 mOsm/liter) or sorbitol was added to isotonic media in two separate replicates] for 12 hours. A BD FACS (fluorescence-activated cell sorting) Aria Verdi cell sorter was used to isolate cells with the lowest 5% and highest 5% of 8xTonE-GFP reporter fluorescence to identify positive and negative regulators, respectively. Guide RNA frequencies in the unsorted and sorted populations were determined by deep sequencing followed by analysis using the MAGeCK computational pipeline (*81*), as described in our previous publications (*27*). Screen results can be found in data S1, and all Fastq files from next-generation sequencing have been deposited into the National Institutes of Health (NIH) Short Read Archive (SRA) with the BioProject ID PRJNA1015695.

### Insertional mutagenesis screens in human haploid (HAP1) cells

HAP1 8xTonE-GFP reporter cells were subjected to pooled insertional mutagenesis using a GT bearing retrovirus (fig. S2A) as described in our previous publications (*28*).

#### Retroviral production and concentration

Each of six T-175 flasks was seeded with 1.5 × 10^7^ HEK293FT cells in antibiotic-free media. When cells reached 80% confluency (~24 hours), they were transfected with a mixture of retroviral transfer plasmids with the GT in two reading frames (3.3 μg each of pGT-mCherry and pGT + 1mCherry), a plasmid carrying packaging genes (4 μg of pCMV-Gag-Pol), a plasmid carrying envelope genes (2.6 μg of pCMV-VSV-G), and a plasmid to enhance translation (1.7 μg of pAdVAntage), all mixed in 450-μl serum-free DMEM containing 45 μl of X-tremeGENE HP DNA transfection reagent. After ~16 hours, virus-containing media was harvested twice, 8 hours apart. Harvested virus was filtered through a 0.45-μm low-protein binding syringe filter and concentrated by ultracentrifugation in sterile Thinwall, Ultra-Clear, 25 mm–by–89 mm Beckman centrifuge tubes with a Beckman SW 32 Ti rotor (65,000*g*, 1.5 hours, 4°C).

#### Insertional mutagenesis of HAP1 cells with GT retrovirus

Each of three T-175 flasks were seeded with 2.0 × 10^7^ HAP1 8xTonE-GFP reporter cells and, after ~16 hours, infected twice, 8 hours apart, with the GT retrovirus. Concentrated virus was resuspended in 76 ml of IMDM containing 10% FBS and polybrene (4 μg/ml); 25 ml of this suspension was added to each of the three flasks of HAP1 8xTonE-GFP reporter cells. Flow cytometry demonstrated that 76.5% of the 8xTonE-GFP HAP1 cells had been successfully transduced by a GT retrovirus based on mCherry fluorescence.

#### Selection scheme for HAP1 cells carrying mutations in genes encoding positive and negative regulators

Seven T-175 flasks were each seeded with 2.0 × 10^7^ cells. The next day, cells (3.0 × 10^7^) from one plate were collected to map insertions in the control, unsorted parent cell population without the application of any stress or selection. Cells in the remaining six flasks were subjected to hypertonic stress [NaCl (100 mOsm/liter) was added to isotonic IMDM media] for 6 hours, and then FACS was used to isolate cells with the 10% lowest GFP reporter fluorescence to identify positive regulators of NFAT5 (Fig. 1G). To identify negative regulators, cells (in a second screen) were exposed to low-level hypertonic stress [NaCl (25 mOsm/liter) was added to isotonic media], followed by sorting to isolate cells with the 10% highest GFP fluorescence. For each sorted population, genomic DNA was extracted from 3.0 × 10^7^ cells to map retroviral insertions in the selected population. The experimental, computational, and statistical methods used to map and compare retroviral insertions in the unsorted and sorted populations have been described in detail previously (*28*). The only difference was that the Intronic Gene-trap Insertion Orientation Bias (IGTIOB) score used to assess the insertion bias (Fig. 1H) scored all GT insertions in the selected population, regardless of orientation. Screen results can be found in data S1, and all Fastq files have been deposited into the NIH SRA with the BioProject ID PRJNA1015695.

### Validation pipeline for gene hits from CRISPR screen

For each candidate gene hit, two separate guides that targeted early exons of all possible transcripts were chosen and individually cloned into px459 (Addgene plasmid #62988). Pooled knockout cell lines were generated by transfection of IMCD3 8xTonE-GFP reporter cells with px459 containing a gene-specific guide, and cells positive for sgRNA and Cas9 expression were selected with puromycin treatment (2 μg/μl) for 3 to 5 days. Knockout efficiency was determined by sequencing PCR products that encompass the sgRNA cleavage site, and cell populations with >90% knockout efficiency (confirmed using the Synthego ICE CRISPR Analysis Tool, https://ice.editco.bio/#/) were immediately tested for NFAT5 GFP reporter fluorescence following exposure to hypertonic stress [NaCl (200 mOsm/liter) added to isotonic media for 7 to 9 hours] by flow cytometry with a BD Accuri C6 Flow Cytometer using a 473-nm laser for excitation and a 520/30-nm bandpass filter to collect emitted light. Two biological replicates were evaluated for each of the two guides specific for each gene (four total independent replicates). All pooled knockout cell populations were compared with cells transfected with a nontargeting control sgRNA.

### Imaging IMCD3 and HEK293T cells using immunofluorescence

Cells adherent to coverslips were washed with 1× PBS and then fixed with 4% (w/v) paraformaldehyde (PFA) in PBS. Following three washes with PBS for 5 min each, coverslips were either directly mounted on glass slides or processed for further staining. For immunofluorescence staining, cells were permeabilized with blocking buffer [1× PBS, 1% normal donkey serum, bovine serum albumin (10 mg/ml), and 0.1% Triton X-100] for 30 min. Cells were incubated with primary antibodies in blocking buffer for 1 to 4 hours at room temperature, washed (3×, 5 min each with wash buffer containing 1× PBS and 0.1% Triton X-100), and then incubated (1 hour, room temperature) with secondary antibodies in blocking buffer. After three additional incubations (5 min each) in wash buffer, coverslips were mounted and sealed on glass slides. Images were collected using a Leica TCS SP8 confocal imaging system equipped with a 63× oil immersion objective. All images were taken of cells fixed with PFA unless noted otherwise.

### Live cell imaging and measuring transcriptional activity of Cry2 fusions

Time-lapse microscopy of activated Cry2 fusions in HEK293T cells was performed using a Leica TCS SP8 confocal imaging system equipped with a 63× oil immersion objective. HEK293T cells were plated in two-well Labtek glass chamber slides (Thermo Fisher Scientific) at 10^4^ cells per well. One day after plating, cells were transfected with Cry2-mCherry NFAT5 fusions mixed with PEI in Opti-MEM. Just before imaging, the medium was replaced by FluoroBrite DMEM without phenol red, and cells were imaged directly in the Labtek glass chamber slides. For live-cell imaging, cells were imaged typically by use of two laser wavelengths (488 nm for Cry2 activation and 560 nm for mCherry imaging).

To measure changes in blue-light dependent changes in NFAT5 transcriptional activity, IMCD3 *Nfat5*^−/−^ cells stably expressing importin-β binding domain from importin-α (IBB)-Cry2-mCherry-NFAT5 were pretreated with the addition of NaCl (100 or 200 mOsm/liter) to media for 30 min. The media was then exchanged with new media without any additional NaCl, and cells were left to incubate in the presence of blue light for 7 hours. Next, RNA was purified from the cells, and 420 ng RNA was used as input for cDNA synthesis.

### Image analysis

Image processing for nuclear NFAT5 levels (Fig. 3H and figs. S4, G and M, and S8, F and I) was carried out using maximum intensity projection (MIP) images of the acquired *z*-stacks using CellProfiler. For quantification of nuclear NFAT5, first a mask was constructed using the 4′,6-diamidino-2-phenylindole (DAPI) image (nuclear marker), and then the mask was applied to the corresponding NFAT5 image where the total fluorescence intensity per cell was measured.

NFAT5 puncta number analysis (Figs. 4, C and I, 5D, 6E, and 8C, and fig. S7A, fig. S8, F and I) was carried out using MIP images of the acquired *z*-stacks using the Analyze Particles function of ImageJ. An automated thresholding function was used for each image before puncta number analysis. For HEK293T cells, a region of interest was drawn around each cell, and the total number of puncta was counted. In case of IMCD3 cells, NFAT5 nuclear puncta numbers were counted for each cell using the DAPI channel as a reference to identify the nuclear boundary.

### Imaging yeast cells

Saturated overnight cultures of yeast cells in CSM + 2% raffinose, 0.1% glucose, and 0.004% adenine were diluted to an OD_600_ of 0.3 (3.0 × 10^6^ cells per ml of media) the next morning in CSM + 2% raffinose and 0.004% adenine lacking glucose. Early log-phase cells were then cultured in CSM supplemented with 2% galactose and 0.004% adenine for 4 hours before microscopy measurements. Cells were stained with Hoechst 33342 (1 μg/ml for 5 min at room temperature) to visualize the nucleus. Cells were mounted in growth medium, and images were collected using an Olympus IX83 epifluorescence microscope equipped with an Orca Fusion sCMOS camera using a 100× oil objective [numerical aperture (NA) 1.45]. Hoechst 33342 and mRuby3 were imaged using the DAPI and tetramethyl rhodamine isothiocyanate (TRITC) channels, respectively. Image analysis and quantification were done using ImageJ.

### Introduction of NFAT5 variants and the NFAT5 transcriptional reporter into yeast

W303a cells were transformed with pAG306-8xTonE-*pCYC1*-GFP, constructed by replacing the *GPD1* promoter in Addgene plasmid #14140 (pAG306-GPD) with a 8xTonE-p*CYC1*-GFP cassette, which was adapted from a yeast transcriptional reporter for calcineurin activity (pAMS366-4xCDRE-GFP-PEST, Addgene plasmid #138658) by replacing the four calcineurin-dependent response elements (CDREs) with eight copies of the TonE binding sequence (5′-TGGAAAATTAC-3′) (*82*). Genomic integration of pAG306-8xTonE-*pCYC1*-GFP was done by linearization of the plasmid with the restriction enzyme Stu I within the URA3 marker resulting in homology to *ura3-1* and transformed into WT W303a cells. Transformed colonies were selected on CSM-Ura plates supplemented with 0.004% adenine and 2% glucose. One colony with the lowest basal GFP expression was used for further experiments. Reporter cells were transformed with pAG305-*pGAL1* plasmids (Addgene plasmid #14137) containing mini-NFAT5 and its variants or other control genes. Genomic integration of the pAG305-*pGAL1* plasmids was done by linearization of the plasmid with the restriction enzyme Bst EII within the LEU2 marker resulting in homology to *leu2-3* and transformed into W303a 8xTonE-*pCYC1*-GFP reporter cells. Transformed colonies were selected on CSM-Ura-Leu plates supplemented with 0.004% adenine and 2% glucose. Gene integration and protein expression were assessed by PCR and Western blotting, respectively.

Mini-NFAT5 (NLS-mRuby3-DBD-MD1-AD2; Fig. 2A) was constructed by fusing a strong NLS [the Importin-β binding domain from importin-α or IBB (*83*)] to the Rel-homology DBD (amino acids 264 to 543) and two previously described regions (MD1: amino acids 618 to 821; AD2: amino acids 1039 to 1245) from the CTD of human NFAT5 shown to be important for responsiveness to hypertonic stress (*30*).

### Disruption of HOG pathway genes in yeast

Mutant strains were generated in WT W303a cells for the sodium measurements using a fluorescent sensor (fig. S3J) and in W303a cells expressing the 8xTonE-GFP reporter and mini-NFAT5 variants (for Fig. 2K and fig. S3, I and K) by homologous recombination of gene-targeted, PCR products using an established method (*84*). Mutant strains were produced by replacing the complete reading frame of target genes with the *KANMX*, *HPHMX*, or *TRP1* cassette. Gene deletions were confirmed by PCR amplification of the deleted locus by assessing HOG pathway target gene (*Stl1*, sugar transporter–like protein) induction by RT-qPCR following a 2-hour treatment with 600 mM (1200 mOsm/liter) NaCl in CSM containing 0.004% adenine and 2% glucose (fig. S3I).

### Intracellular sodium measurements in yeast using a fluorescent sensor

W303a cells (WT and mutant) cultured overnight in CSM supplemented with 2% glucose and 0.004% adenine were diluted to 2.0 × 10^6^ cells per ml. After 4 hours, cells were pelleted and resuspended in fresh isotonic media containing the ION Natrium Green - 1 (ING-1) AM sodium sensor (ION Biosciences) in 96-well black-bottom plates. The stock solution of the sodium sensor was prepared according to the manufacturer’s protocol. Briefly, ING-1 AM was dissolved in dimethyl sulfoxide and used at a final concentration of 5 μg/ml in isotonic media with 0.02% Pluronic F-127 (Invitrogen). Cells were then incubated at 30°C for 1 hour with gentle shaking and further grown in isotonic media or media containing additional NaCl (400 to 1600 mOsm/liter; to apply hypertonic stress) for 30 min. Fluorescence was measured on a Synergy H1 plate reader (BioTek) using an excitation wavelength of 520 nm and an emission wavelength of 550 nm. All readings were corrected for background fluorescence and measured from cells incubated without the sodium sensor.

### Measurements of intracellular sodium content in yeast using inductively coupled optical emission spectroscopy

Following a previously published protocol (*85*), 5.0 × 10^8^ of log-phase yeast growing in normal media (CSM with 0.004% adenine and 2% galactose) or media containing additional NaCl (400 or 1600 mOsm/liter) for 2 hours were collected by centrifugation (8000*g* spin for 3 min at 4°C) and washed twice in ice-cold wash buffer containing 1.5 M sorbitol and 20 mM MgCl_2_. Cell pellets were then resuspended in 1 ml of 0.2-μm filtered water, mixed with acid-washed glass beads, vortexed for 3 min, and then boiled for 30 min at 95°C with shaking. Following a 1000*g* spin to separate the supernatant from the beads and cell debris, samples were cleared using ultracentrifugation with a 100,000*g* spin for 30 min at 4°C. Cleared samples were then diluted to 5 ml with water, passed through a 0.45-μm filter, and analyzed using an inductively coupled plasma optical emission spectrometer (iCAP 6000 Series, Thermo Fisher Scientific) through the Environmental Measurements Facility at Stanford University with the following settings: flush pump rate at 50 rpm, analysis pump rate at 50 rpm, pump stabilization time at 5 s, radio frequency power at 1150 W, auxiliary gas flow at 0.5 liters/min, and nebulizer gas flow at 0.5 liters/min. Measurements of sodium in samples were calculated on the basis of a standard curve, with NaCl diluted at various known concentrations in filtered water.

### Isotonic shrinkage of IMCD3 cells

The osmolarity of the various solutions used in this protocol was measured using the Fiske Micro-Osmometer (model 210). The protocol to induce shrinkage of cells without changing intracellular ionic strength was adapted from previous studies (*9*, *34*). *Nfat5*^−/−^ IMCD3 cells expressing FLAG-mVenus-NFAT5 or *Wnk1*^−/−^ IMCD3 cells expressing GFP-WNK1 were incubated for 10 min with in a hypotonic medium [osmolarity of 200 mOsm/liter containing 1× MEM essential amino acid (Gibco), 1× MEM nonessential amino acid (Gibco), 1× l-glutamine (Gemini Biosciences), 1× MEM vitamin solution (Gibco), 10 mM glucose (Gibco), 50 mM Hepes (pH 7.4) (Gibco), 1 mM sodium pyruvate (Gibco), 10% FBS, 2 mM CaCl_2_, 1 mM MgSO_4_, 5 mM KCl, and 30 mM NaCl]. During the last 5 min of hypotonic exposure, an RVI inhibitor cocktail (20 μM benzamil, 100 μM bumetanide, and 200 μM gadolinium) was added. Hypotonic media was then exchanged for isotonic media (~300 mOsm/liter) containing the same RVI inhibitor cocktail, and cells were analyzed after 30 min at 37°C. Cells subjected to the isotonic shrinkage protocol were compared to cells treated with hypotonic, isotonic, or hypertonic media all with or without RVI inhibitors.

### Ionic stress imposition on IMCD3 cells using nystatin

We adapted a previous protocol that used nystatin to increase intracellular ionic strength (*42*). Custom isotonic and hypertonic buffers with either high KCl (iso-KCl and hyper-KCl) or high NaCl (iso-NaCl and hyper-NaCl) were prepared from the same stock solutions. Osmolarities of all buffers were measured using a Fiske Micro-Osmometer (Mmdel 210). All buffers contained 1× MEM essential amino acid, 1× MEM nonessential amino acid solution, 1× l-glutamine, 1× vitamin solution, 10 mM glucose, and 10 mM Hepes (pH 7.4). Iso-NaCl (286 mOsm/liter) contained 5 mM KCl, 122 mM NaCl, 1 mM CaCl_2_, and 1 mM MgCl_2_. Iso-KCl (289 mOsm/liter) contained 89 mM KCl, 5 mM NaCl, and 50 mM sucrose. Hyper-NaCl (505 mOsm/liter) contained 5 mM KCl, 240 mM NaCl, 1 mM CaCl_2_, and 1 mM MgCl_2_. Hyper-KCl (503 mOsm/liter) contained 205 mM KCl, 5 mM NaCl, and 50 mM sucrose.

*Nfat5*^−/−^ IMCD3 cells stably expressing FLAG-mVenus-NFAT5 or *Wnk1*^−/−^ IMCD3 cells stably expressing GFP-WNK1 cells were plated on coverslips 1 day before the experiment. Four total treatments (four different wells of cells) per cell line were compared in this experiment: (1) isotonic, (2) hypertonic, (3) isotonic + nystatin, and (4) hypertonic+nystatin. The day after plating cells, the iso-NaCl buffer was added to all wells and after 10 min at 37°C. Next, samples (3) and (4) were treated with ice-cold iso-KCl buffer containing nystatin (50 μg/ml). Following a 10-min incubation on ice, the media in sample (4) was exchanged to ice-cold hyper-KCl buffer containing nystatin (50 μg/ml). All cells were left to incubate on ice for 20 min. After 20 min, samples (1) and (3) were washed with warm iso-NaCl, and samples (2) and (4) were washed three times with warm hyper-NaCl to remove the nystatin. After a 30-min incubation in hyper-NaCl (samples 2 and 4) or iso-NaCl (samples 1 and 3) at 37°C, coverslips were washed twice with PBS, and cells were fixed with 4% (w/v) PFA at room temperature. Instead of treatment with nystatin, the isotonic (sample 1) and hypertonic (sample 2) control cells were kept in either iso-NaCl or hyper-NaCl but were otherwise exposed to the same temperature changes and final buffers as the nystatin-treated cells.

### Measurement of cell height

The cell height was used as a rapid, convenient proxy for cell volume based on previous studies showing that cell height (or thickness) can be used to estimate relative changes in the volume of adherent cells grown in a monolayer (*86*–*88*). CellMask Deep Red Plasma membrane stain (Thermo Fisher Scientific) was used to visualize the plasma membrane to measure cell heights in the *xz* plane of confocal image stacks. The CellMask stain was added to cells 15 min before beginning all protocols (isotonic shrinkage, nystatin treatments, or salt treatments) and excess stain removed by three washes in isotonic media. Cell height was measured from the flat, basal edge of the cell touching the coverslip to the peak height of the cell along the *z* axis using ImageJ. Measurements were normalized to the median cell height of the control sample (untreated; samples in isotonic media).

### Measurement of cell volume

IMCD3 cells were plated at a low density in two-well Lab-Tek chambers (500 cells per well) to obtain single separated cells for volume analysis. Cells were labeled with fluorescein diacetate (50 μg/ml; FDA) in DMEM/F12 medium for 1 hour in a humidified atmosphere with 5% CO_2_ at 37°C. After washing away excess FDA, cells were switched to hypertonic medium (200 mOsm/liter NaCl, NH_4_OAc, sorbitol, or urea added to isotonic media) for imaging. All images were acquired on a Leica TCS SP8 confocal imaging system equipped with a 63× oil immersion objective. Fluorescein was imaged using an excitation wavelength of 488 nm and collecting light emitted between 500 and 600 nm. Images of *z* sections of the entire cell were acquired with a fixed step size of 0.5 μm. Individual cell volume was measured using the three-dimensional (3D) object counter plugin in Fiji (NIH, Bethesda) after fluorescence intensity thresholding (above background, determined empirically for each image) of *z*-sections.

### Genetically encoded sensor for intracellular ionic strength

HEK293T cells were plated in two-well Labtek glass chamber slides (Thermo Fisher Scientific) at 10^5^ cells per well. One day after plating, cells were transfected with pcDNA3.1_ionRD (Addgene plasmid #172931) mixed with PEI in Opti-MEM. The next day, the medium was replaced by FluoroBrite DMEM without phenol red, and cells were imaged directly in the Labtek glass chamber slides. The sensor was excited using a 405-nm laser, and the emission was split into a 450- to 505-nm channel (to image mCerulean3) and a 505- to 797-nm channel (to image mCitrine). The fluorescence intensity of the cells was determined in ImageJ for each channel. The backgrounds for each channel were subtracted, and the mCitrine intensity was divided by the mCerulean3 intensity for each cell.

### Endogenous tagging of NFAT5

To generate an IMCD3 clonal cell line with an endogenously tagged *Nfat5* alleles, *Sp*Cas9 protein was complexed with sgRNA (5′-GGG-TCGAGCTGCGATGCCCT-3′) and electroporated with the homology-directed repair template plasmid (pUC19-NFAT5/mNG with ~700-bp left and right homology arms flanking mNG for N-terminal tagging of NFAT5). Production of sgRNA and *Sp*Cas9 was done as described previously (*89*). For electroporation, Cas9–ribonucleoprotein (RNP) complex was prepared by mixing 100 pmol of *Sp*Cas9 protein with 120 pmol of sgRNA in Cas9 buffer [20 mM Hepes (pH 7.4), 150 mM KCl, 1 mM MgCl_2_, 10% glycerol, and 1 mM tris(2-carboxyethyl)phosphine (TCEP)] for 10 min. The RNP complex was added to 500,000 low-passage IMCD3 cells and electroporated using a NEPA21 Electro-Kinetic transfection system (Bulldog-Bio). Ninety-six hours postelectroporation, mNG-positive single cells (top 0.1% fluorescence intensity) were sorted into a 96-well plate using a FACS (SH800S, Sony). Positive clonal lines were identified by a dual genomic PCR strategy. “In-In” PCR [forward primer complementary within the NFAT5 left homology arm (LHA) (5′- GGAGTCAGTTCTCCACTCCG-3′) and reverse primer complementary within the NFAT5 right homology arm (5′-GCGATTCCAGGTCTAGGTCC-3′)] was used to confirm genomic integration of the homology repair template. “In-Out” PCR [forward primer complementary to the genomic region outside the LHA (5′-GGTGTGGTGGGTAACGTGG-3′) and reverse primer complementary within mNG (5′-GGCCGACCATATCGAAGTCT-3′)] was used to confirm the correct site of integration in the *Nfat5* gene (fig. S6C). Expression of the mNG-NFAT5 fusion protein was confirmed by immunoblotting using an antibody against NFAT5 (Bethyl Laboratories) (fig. S6D).

### Gal4 DBD luciferase reporter assays

HEK293T cells grown in each well of a 96-well plate were transfected with 40 ng of GAL4 upstream activation sequence–driven firefly luciferase expression construct (Addgene plasmid #64125), 40 ng of pGAL4-DBD-MCS-long plasmids (Addgene plasmid #145246) directing expression of NFAT5 fragments fused to the GAL4 DNA binding domain driven by the cytomegalovirus promoter, and 5 ng of herpes simplex virus thymidine kinase (HSV TK) promoter::renilla luciferase plasmids directing constitutive renilla luciferase expression. Cells were cultured for 24 hours, exposed to isotonic or hypertonic media [NaCl (200 mOsm/liter) or NH_4_OAc (100 mOsm/liter) was added to isotonic media] and then lysed in passive lysis buffer (25 μl per well, Promega Dual-Luciferase Reporter Assay System) according to the manufacturer’s instructions. Luciferase activities were measured using the Dual-Luciferase Assay System (Promega) in a Synergy H1 microplate reader (BioTek) equipped with an automatic injector. For each sample, NFAT5-GAL4_DBD_–driven firefly luciferase activity was normalized to HSV TK::renilla luciferase activity (to correct for differences in transfection), and the resulting ratio was reported in normalized luciferase intensity units.

### Protein expression and purification

DNA fragments encoding NFAT5 CTD and PLD (and their variants) were cloned into pET-28a plasmid with C-terminal 6xHis-tag either with or without an N-terminal fusion to superfolder GFP (sfGFP) for fluorescence-based phase separation assays. These bacterial expression plasmids were transformed into *E. coli*–competent cells (BL21 [DE3] pLysS, MilliporeSigma) and plated on LB agar plates containing kanamycin (50 μg/ml) and chloramphenicol (34 μg/ml). A single colony was picked and grown overnight in 10 ml Terrific broth (TB) containing kanamycin (50 μg/ml) and chloramphenicol (34 μg/ml) and used to inoculate a 1-liter culture of TB. When the culture reached an OD_600_ of 0.4 to 0.6, protein expression was induced with 0.25 mM isopropyl-β-d-thiogalactopyranoside at 37°C for 4 hours. After the addition of 1 mM phenylmethylsulfonyl fluoride (PMSF) and 1 mM EDTA to the cultures, cells were harvested at 4000*g* for 15 min at 4°C and flash-frozen in liquid nitrogen.

For protein purification, bacterial pellets were lysed in B-PER Complete Bacterial Protein Extraction Reagent (Thermo Fisher Scientific) (5 ml reagent per gram of cell pellet) supplemented with 1 mM PMSF, 1× protease inhibitor (SigmaFast Protease inhibitor cocktail, EDTA-free; Sigma-Aldrich), and 1 mM EDTA. To facilitate lysis, cells were incubated at room temperature for 15 min with gentle rocking. The CTD and PLD proteins were in insoluble inclusion bodies, which were collected by centrifugation at 20,000*g* for 30 min at 4°C. The pellets containing inclusion bodies were washed by resuspension using a Dounce homogenizer in 10 pellet volumes in wash buffer [50 mM tris (pH 7.5), 1 mM EDTA, 5 mM DTT, 1 M urea, 1% Triton X-100, 1 mM PMSF, and SigmaFast protease inhibitor cocktail] until a clear supernatant was left after pelleting the inclusion bodies. The washed inclusion body pellet was lastly suspended in the wash buffer minus the Triton X-100 and urea and collected by centrifugation at 20,000*g* for 30 min at 4°C. The washed inclusion body pellet was solubilized using a Dounce homogenizer with 1 ml of extraction buffer [50 mM tris (pH 7.5), 8 M urea, 1 mM PMSF, and 1 mM DTT] per gram wet weight of original cells and incubated at room temperature for 1 hour with gentle rocking. The suspension was centrifuged for 1 hour at 100,000*g* at 4°C, and the supernatant was filtered through a 0.22-μm syringe filter attached to a disposable syringe and loaded on a Superdex 200 16/60 column (GE Healthcare) equilibrated with a low ionic strength gel filtration buffer [20 mM Hepes (pH 7.4) and 1 mM TCEP]. Fractions containing NFAT5 CTD and PLD proteins were identified by SDS-PAGE followed by Coomassie Blue staining, pooled, and concentrated using Amicon Ultracel 30K filters (MilliporeSigma). Proteins were stored at concentrations ≥20 mg/ml on ice and diluted immediately before use.

### In vitro phase separation assays

Recombinant NFAT5 CTD and PLD fused to sfGFP fusion proteins at varying concentrations were mixed with 5% dextran (a molecular crowding agent) in droplet buffer [20 mM Na-Hepes (pH 7.4) and 1 mM TCEP], and NaCl (or other salts) were added from a concentrated stock to achieve the indicated osmolarity. The protein solution was incubated for 10 min at room temperature and loaded onto a glass slide with a coverslip. Slides were then imaged with a 10× dry objective (NA 0.3) on an Olympus IX83 epifluorescence microscope equipped with an Orca Fusion scMOS camera. Droplets containing sfGFP were imaged using the fluorescein isothiocyanate (FITC) channel. Droplets without GFP were visualized using the brightfield channel. Unless indicated, images presented are of droplets settled on the glass coverslip. All assays were performed at room temperature.

To pellet condensates formed by NFAT5 GFP-CTD (Fig. 5G), droplet reactions [containing 5% dextran, 70 μM GFP-CTD, and NaCl (1000 mOsm/liter) in droplet buffet] were centrifuged at 20,000*g* for 10 min. The supernatant was removed, and condensate pellets were resuspended in droplet buffer [20 mM Na-Hepes (pH 7.4) and 1 mM TCEP] with 5% dextran (but no added NaCl) to determine whether the condensates were reversible.

### Transcriptional coactivator recruitment assay in U2OS cells

U2OS cells containing a stably integrated array of ∼20,000 TetR binding sites (*73*) into a single genomic locus were grown in coverslips and transfected using Lipofectamine 3000 with 500 ng of pNLS-TetR-GFP (Addgene plasmid #103838) directing expression of NFAT5 CTD or PLD fused to EGFP-tagged TetR that is constitutively recruited tetO arrays. As a control, the constitutive transcriptional activator VP16 was transfected using pEGFP-TetR-NLS-VP16 (Addgene plasmid #103834). Cells were cultured for 48 hours, exposed to isotonic or hypertonic media [NaCl (200 mOsm/liter) was added to isotonic media] for 1 hour, were fixed for immunofluorescence with a MED1 or BRD4 antibody (table S1), and imaged by confocal microscopy. To calculate enrichment (Fig. 8B and fig. S12, C and E, and S13, C and E) of MED1 or BRD4 in the single dot of the single genomic locus, dots were identified as a region of interest in FIJI by the EGFP-TetR image (green channel), and the mean fluorescence signal of MED1 or BRD4 (red channel) within that dot was determined. The enrichment was calculated by dividing the MED1 or BRD4 fluorescence intensity by the background MED1 or BRD4 signal in the nucleus and was normalized to the EGFP-TetR intensity.

### Fluorescence recovery after photobleaching

FRAP studies were performed by live confocal fluorescence microscopy using a Leica TCS SP8 confocal imaging system. Images were acquired with a 63× oil immersion objective every 600 ms for 1 min. FRAP was performed using the FRAP module of the Leica Application Suite X software with the 488-nm laser at 100% power, 200-μs dwell time. Fluorescence recovery was subsequently recorded every 600 ms for a total of 1 min. Image series were imported into FIJI ImageJ for analysis. To calculate the recovery after photobleaching, additional bleaching during image acquisition was corrected using a control nonbleached region over time. To compare multiple experiments, the normalized fluorescence intensity was integrated over all experiments and plotted against time. The prebleach signal intensity was set to 100% and the postbleach intensity to 0% for normalization. Data were plotted as percent recovery versus time in using Prism 10 (GraphPad).

### Super-resolution imaging

Super-resolution 3D structured illumination microscopy images (Fig. 4H) were collected as Z-stacks (0.125 μm) using a 100× NA 1.40 objective on a DeltaVison OMX Blaze microscopy system, deconvolved, and corrected for registration using SoftWoRx. Final assembly of 2D MIPs was performed using Fiji.

### Proteomics of proximal proteins to mini-NFAT5

#### Proximity labeling of mini-NFAT5 interacting proteins using TurboID

IMCD3 *Nfat5*^−/−^ or NIH/3 T3 *Nfat5*^−/−^ cells stably expressing IBB-TurboID-1D4 or IBB-TurboID-mini-NFAT5-1D4 were generated via integration to the Flp-In locus. To prepare samples for mass TMT labeling and mass spectrometry analysis of enriched peptides, the following procedure was performed. Thirty-two 15-cm plates were each seeded with 2.25 × 10^6^ IMCD3 or NIH/3 T3 *Nfat5*^−/−^ cells expressing IBB-TurboID-mini-NFAT5-1D4 (experimental sample). Twenty-four 15-cm plates were each seeded with 2.25 × 10^6^ IMCD3 or NIH/3 T3 *Nfat5*^−/−^ cells expressing IBB-TurboID-1D4 (spatial control). Last, eight 15-cm plates were each seeded with 2 × 10^6^ IMCD3 or NIH/3 T3 *Nfat5*^−/−^ cells (no ligase control). Three days later, all cells were treated with 100 μM biotin for 2 hours. Half of each set of plates per cell line expressing variants of TurboID were treated with 100 μM biotin only (isotonic samples). All eight plates per cell line expressing no TurboID ligase variant were treated with 100 μM biotin with the addition of NaCl (200 mOsm/liter; 100 mM). After 2 hours, each plate was washed with 1× ice-cold Dulbecco’s phosphate buffered saline (DPBS) for a total of five times. Cells were collected, pooled, and pelleted in 1× DPBS. After removing the DPBS supernatant, the pellets were resuspended in 3.5 ml (5 pellet volumes) of 1× RIPA lysis buffer [50 mM tris-HCl (pH 7.4), 150 mM NaCl, 0.1% SDS, 0.5% sodium deoxycholate, 1% Triton X-100, 1 mM DTT, and 1× SigmaFast protease inhibitor cocktail]. Tubes were rotated at 4°C for 30 min. Next, samples were cleared following a 20-min spin at 100,000*g* at 4°C. Two hundred eighty microliters of Pierce streptavidin–coated magnetic beads (catalog #88816) were mixed with 7 mg of protein for each IP. The following summarizes the number of replicates that were set up: four replicate IPs for each cell line expressing IBB-TurboID-mini-NFAT5 treated with 100 mM NaCl (hypertonic media), three replicate IPs for each cell line expressing IBB-TurboID-mini-NFAT5 in isotonic media, three replicates for each cell line expressing IBB-TurboID-1D4 in isotonic or hypertonic media, and two replicates for each cell line expressing no ligase in hypertonic media. Samples were left to incubate overnight on a rotator at 4°C. The next day, beads were washed twice with 1 ml of RIPA buffer at 2 min per wash, once with 1 ml of 1 M KCl for 2 min, once with 1 ml of 0.1 M Na_2_CO_3_ for 10 s, once with 1 ml of 2 M urea in 10 mM tris-HCl (pH 8.0) for 10 s, and then twice with 1 ml of RIPA buffer at 2 min per wash. After transferring samples to new tubes, the supernatant was removed, and beads were pelleted and snap-frozen.

#### Digestion

Proteins were eluted from the beads and digested using S-Trap (Protifi). Beads were resuspended in 100 μl of lysis buffer [50 mM triethylammonium bicarbonate (TEAB) buffer, 5% SDS, in liquid chromatography–mass spectrometry (LC-MS)–grade water, un-pHed] supplemented with 10 mM TCEP and 20 mM chloroacetamide and incubated for 30 min at 37°C. Samples were then acidified using 10 μl of 12% phosphoric acid and placed on a magnetic rack. Eluates were transferred to clean tubes, and beads were washed with 700 μl of S-Trap binding buffer (50 mM TEAB in 90% LC-MS–grade methanol, un-pHed). Eluates and binding buffer were then combined, loaded onto S-Trap mini cartridges, and washed four times with 400 μl of binding buffer. S-traps were then supplemented with 125 μl of 50 mM TEAB containing 10 μg of trypsin (Sigma-Aldrich, #T1426) and incubated for 2 hours at 47°C. Peptides were then eluted according to manufacturer’s recommendations and dried down by SpeedVac (Thermo Fisher Scientific).

#### TMT labeling

Dried peptides were reconstituted in 70 μl of 50 mM TEAB and labeled by adding 10 μl of TMT reagent at LC-MS–grade acetonitrile (20 μg/μl and 20 μl). After 2 hours under agitation at room temperature, the reaction was quenched by adding 5 μl of 5% hydroxylamine for 15 min. Samples were then combined in a 1:1 ratio, and labeled peptides were dried down by SpeedVac.

#### Offline fractionation

Peptide were resuspended in 100 μl of 50 mM ammonium acetate at pH 10 and injected on a XBridge Peptide BEH C18 column (1 mm by 100 mm, 3.5-μm particle size, 130-Å pores; Waters #186003561). Peptides were eluted from the column using basic reverse-phase fractionation (using 10 mM ammonium acetate in water as buffer A and 10 mM ammonium acetate in 80:20 acetonitrile water as Buffer B) on a 55-min multistep gradient at 100 μl/min (from 3 to 10, 40, 60, and 100% buffer after at 20, 25, 65, 70, and 75 min, respectively). Eluted peptides were collected from 26 to 82 min into 96 fractions and pulled into 24 fractions using nonconsecutive concatenation (fraction 1 was pulled with 25, 49, and 73). The 24 fractions were then dried down by SpeedVac and stored at −20°C until liquid chromatography–tandem mass spectrometry (LC-MS/MS) analysis.

#### LC-MS/MS analysis

Fractionated peptides were resuspended in 20 μl of 5% formic acid in water and injected on a UltiMate 3000 RSLCnano System coupled to a Orbitrap Fusion Lumos Tribrid Mass Spectrometer (Thermo Fisher Scientific). Peptides were loaded on an Acclaim Pepmap 100 trap column (100 μm by 2 cm, 5-μm particle size, 100-Å pores; Thermo Fisher Scientific #164564-CMD) for 5 min at 10 μl/min before analysis on a PepMap RSLC C18 analytical column (75 μm by 50 cm, 2-μm particle size, 100-Å pores; Thermo Fisher Scientific, #ES903) and eluted on a 120-min linear gradient from 3 to 35% buffer B [buffer A: 0.1% formic acid in water, buffer B: 0.08% formic acid in 80:20 acetonitrile:water (v:v)]. Eluted peptides were then analyzed by the mass spectrometer operating in synchronous precursor selection (SPS) mode on a TOP 3 s method. MS1 were recorded at a resolution of 120,000 at mass/charge ratio (*m/z*) 200 using an automatic gain control (AGC) target of 100% and a maximum injection time (IT) of 50 ms. Precursors were selected in a data-dependent manner using an AGC target of 100% and a maximum IT of 50 ms for MS2 fragmentation using HCD at a normalized collision energy (NCE) of 32% and analyzed in the ion trap operating in rapid mode. For SPS, up to 10 fragment ions were selected for MS3 fragmentation using an AGC target of 200%, a maximum IT of 120 ms, and a NCE of 55%. MS3 fragments were then analyzed in the Orbitrap using a resolution of 50,000 at *m/z* 200.

#### Data analysis

Peptide were searched against the UniProt Swiss-Prot Mouse database (released on 06 January 2021) using MaxQuant (v1.6.17.0) (*90*). Statistical analysis was carried out using Python (v3.9.0) and packages pandas (v1.3.1), numpy (v1.21.1), and scipy (v1.7.1). Protein groups identified as reverse, only identified by site, potential contaminants, with a single razor + unique peptide or quantified in less than three replicates in at least one condition were removed. Protein intensities were median-normalized, and missing values were then imputed using a Gaussian distribution centered on the median with a downshift of 1.8 and a width of 0.3 (relative to the SD). Protein regulation was assessed using a two-sample Welch test, and *P* values were adjusted using Benjamini Hochberg multiple hypothesis correction. The mass spectrometry proteomic data have been deposited to the ProteomeXchange Consortium via the PRIDE (*91*) partner repository with the dataset identifier PXD052591. Data can also be found at the following links: curtain link 3T3: https://curtain.proteo.info/#/abf332b3-ec45-46df-8dfc-beda951b9697; curtain link IMCD3: https://curtain.proteo.info/#/77441e7e-57e8-451f-8a3b-8fbf035b38aa. See data S3 for raw peptide counts and processed results.

To identify proteins up-regulated in the experimental (cells expressing IBB-TurboID-mini-NFAT5) and spatial control (IBB-TurboID) cell lines, various filtering steps were implemented as outlined in (*71*). First, peptides in all samples were filtered if the number of unique peptides was greater than or equal to 2. Next, proteins were compared between the experimental and spatial controls for each treatment. All proteins with an average log_2_ fold change of greater than 0 after comparing set A (IBB-TurboID-miniNFAT5; isotonic) with set C (IBB-TurboID; isotonic) and set B (IBB-TurboID-miniNFAT5; hypertonic) with set D (IBB-TurboID; hypertonic) were filtered. The filtered list in set A was then compared with the filtered list in set B. This list was used as input for GSEA analysis in fig. S11D and to produce the volcano plots in fig. S11C (*92*). The plot depicted in fig. S11E was produced using Rtoolbox developed by PeeperLab (https://github.com/PeeperLab/Rtoolbox).

### Statistical analysis

Data analysis and visualization were performed in GraphPad Prism 10. Model figures (Figs. 1, A, D, and G; 2, D and J; 3, A and E; 5E; and 8E and figs. S2A, S10B, S12A, and S13A) were made in Adobe Illustrator 2023 with icons taken from Biorender.com. The predicted human NFAT5 structure was generated using AlphaFold.

The one-way analysis of variance (ANOVA) test with Sidak’s multiple comparisons or the Kruskal-Wallis test with Dunn’s multiple comparisons was used to compare three or more groups with one independent variable. A two-way ANOVA test with Sidak’s multiple comparisons was used to compare three or more groups with two independent variables. All comparisons shown were prespecified. All experiments were performed at least three different times with similar results. We note that a small sample size (*n* = 3) makes it difficult to assess whether the variance between different samples is comparable. Throughout the paper, the *P* values for the comparisons from GraphPad Prism 10 are denoted on the graphs according to the following key: ****P value < 0.0001, ***P value < 0.001, **P value < 0.01, *P value < 0.05, and nonsignificant. Replicates: In Figs. 1 (B to D and F), 2 (B and C), 6 (A and B), 7E, and 8D and figs. S1A, S2E, S3 (F, I, and K), S8 (A, G, and J), S10D, S11 (F and G), and S13H, bars and solid horizontal lines denote the mean value from three independent experiments unless otherwise indicated. In Figs. 3 (C, D, H, and K), 4I, 5 (D and E), and 8 (B and C) and figs. S4 (B, E, G, K, and M), S6E, S7A, and S8 (F and I), solid horizontal lines denote the median value from >3 independent measurements shown as points.

Genome-wide loss-of-function CRISPR screens were performed twice under independent conditions, and the duplicates from each screen were analyzed together using the MAGeCK tool (fig. S2B). The haploid retroviral mutagenesis screen was performed once. For CRISPR screen validation (fig. S2D), two cell lines expressing different sgRNAS against each candidate gene were generated. Cell lines expressing Cas9 and the sgRNA were analyzed two independent times.

## Supplementary Materials

### The PDF file includes:

Figs. S1 to S13 Tables S1 to S5 Legends for movies S1 to S6 Legends for data S1 to S3 References
