# Supplementary material for: Direct ionic stress sensing and mitigation by the transcription factor NFAT5
Source: Sci Adv. 2025 Feb 19;11(8):eadu3194. doi: 10.1126/sciadv.adu3194 (PMC11838016; doi:10.1126/sciadv.adu3194)
Supplement: Supplementary file 1 — Figs. S1 to S13 Tables S1 to S5 Legends for movies S1 to S6 Legends for data S1 to S3 References [file sciadv.adu3194_sm.pdf]

Science Advances
AAAS

Supplementary Materials for

Direct ionic stress sensing and mitigation by the transcription factor NFAT5

Chandni B. Khandwala *et al.*

Corresponding author: Rajat Rohatgi, rrohatgi@stanford.edu

Sci. Adv. **11**, eadu3194 (2025)
DOI: 10.1126/sciadv.adu3194

The PDF file includes:

Figs. S1 to S13
Tables S1 to S5
Legends for data S1 to S3
Legends for movies S1 to S6
References

Other Supplementary Material for this manuscript includes the following:

Data S1 to S3
Movies S1 to S6

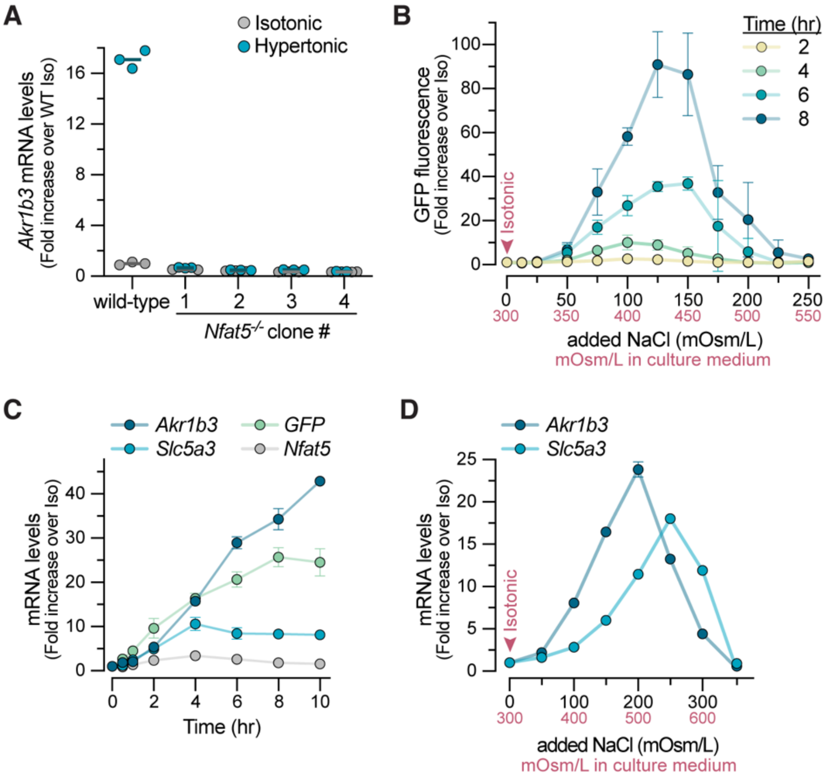

fig. S1. Characterization of NFAT5 reporter cell lines, Related to Fig. 1.

(A) NFAT5 target gene expression measured by RT-qPCR in wild-type (WT) IMCD3 cells and four independent *Nfat5*$^{-/-}$ clonal cell lines after 8 hrs in isotonic media (300 mOsm/L) or in hypertonic media (+200 mOsm/L NaCl, raising total media osmolarity to 500 mOsm/L). Black horizontal lines denote mean values calculated from 3 independent measurements shown as points.

(B) 8xTonE-GFP reporter activity in human haploid reporter cells (HAP1-G, **Fig. 1D**) at the indicated time points in response to increasing amounts of NaCl added to isotonic media. Each point shows the mean ± SD of three independent median measurements, each from a population of >2000 cells. Total media osmolarity at each concentration of NaCl is shown in red on the secondary x-axis.

(C) Abundance of *Akr1b3*, *Slc5a3*, *Nfat5,* and the *GFP* reporter mRNA was measured using RT-qPCR in IMCD3-G cells after exposure to hypertonic stress (+200 mOsm/L NaCl) for the indicated time periods. Each point shows the mean ± Standard Deviation (SD) of three independent measurements.

(D) Expression of *Akr1b3* and *Slc5a3* in WT IMCD3 cells treated with increasing amounts of NaCl added to isotonic media for 8 hrs. Each point shows the mean ± SD of three independent measurements.

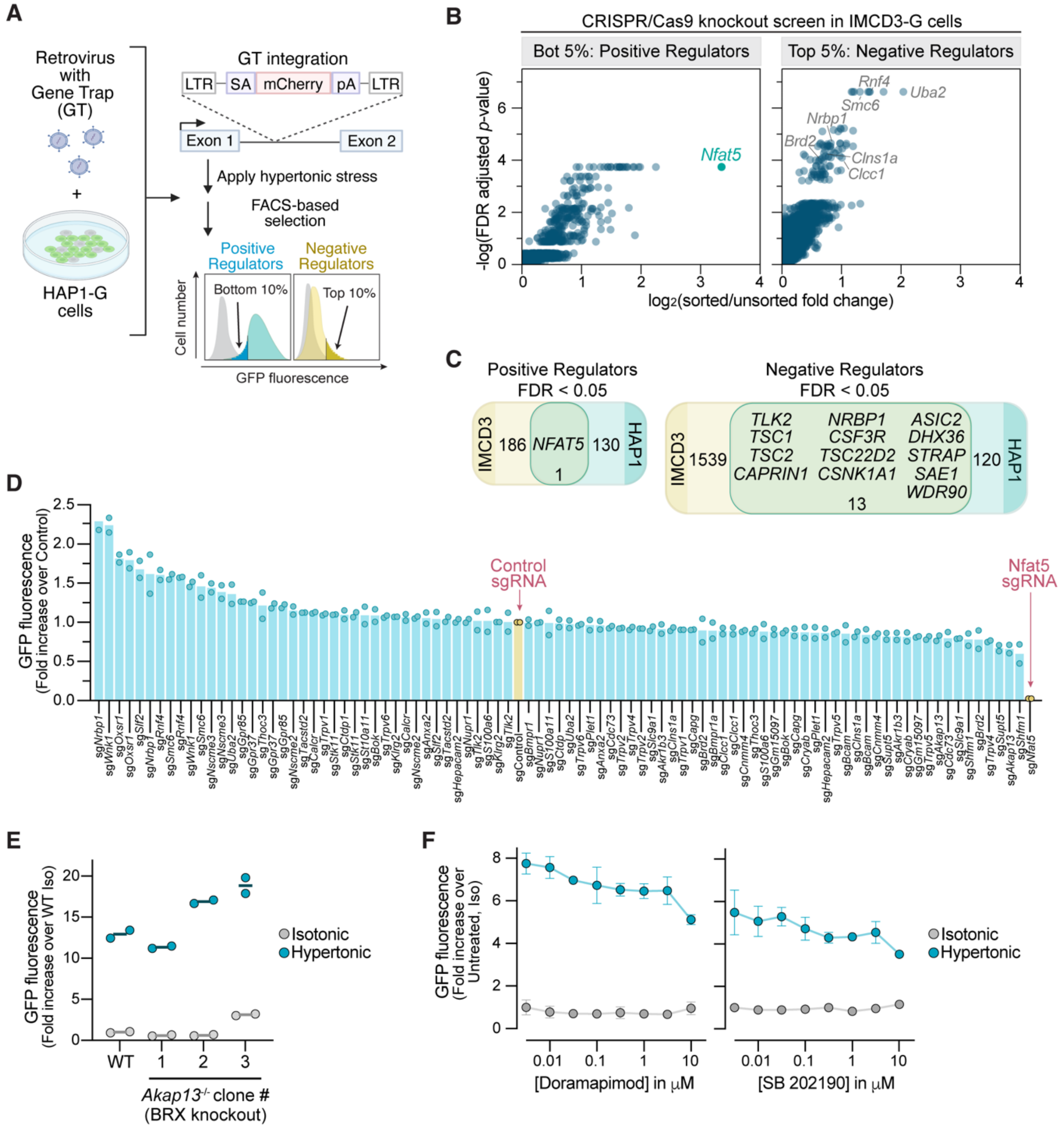

fig. S2. Genome-wide screens for NFAT5 regulators, Related to Fig. 1.

(A) Strategy used for the retroviral insertional mutagenesis screen in human HAP1-G cells using the stably integrated 8xTonE-GFP reporter. Insertion of the gene-trap (GT) cassette, positioned between the retroviral long terminal repeats (LTRs), in the sense orientation into an intron will trigger transcriptional termination: the splice acceptor (SA) will cause this cassette to be spliced to the preceding exon, leading to transcriptional termination due to a strong polyadenylation (pA) signal. Fluorescence activated cell sorting (FACS) was used to collect cells carrying mutations in

genes encoding positive or negative regulators of the NFAT5 transcriptional response following exposure to hypertonic stress (see **Fig. 1G**).
(B) Volcano plots showing results from the CRISPR genome-wide knockout genetic screen in IMCD3-G cells outlined in **Fig. 1G**. The x-axis shows the enrichment of each gene (calculated as the mean of all four sgRNAs targeting the gene) in the sorted population relative to the unsorted population and the y-axis shows statistical significance as measured by the FDR-adjusted *p*-value.
(C) Venn diagrams representing overlapping positive and negative regulators with FDR-corrected *p*-values < 0.05 from the independent screens conducted in IMCD3-G and HAP1-G cells.
(D) 8xTonE-GFP reporter activity after exposure to hypertonic media (+200 mOsm/L NaCl, 8 hrs) in IMCD3-G cells expressing Cas9 and a non-targeting control sgRNA or sgRNAs targeting the indicated gene hits from the CRISPR screen (**B**) on the x-axis. Each point shows an independent median measurement from a population of >2000 cells.
(E) 8xTonE-GFP reporter activity measured by flow cytometry in WT IMCD3-G cells or three independent $Akap13^{-/-}$ IMCD3-G clonal cell lines measured after 8 hrs in isotonic or hypertonic (+200 mOsm/L NaCl) media. AKAP13 is a putative NFAT5 regulator described in the literature (*95*). Each point shows a median measurement from a population of >2000 cells; black horizontal lines show the mean of these independent median values.
(F) 8xTonE-GFP reporter activity in IMCD3-G cells measured by flow cytometry after 8 hrs in isotonic or hypertonic (+200 mOsm/L NaCl) media in the presence of increasing concentrations of the p38 inhibitors doramapimod or SB 202190. Each point shows the mean ± SD of three independent median measurements, each from a population of >2000 cells.

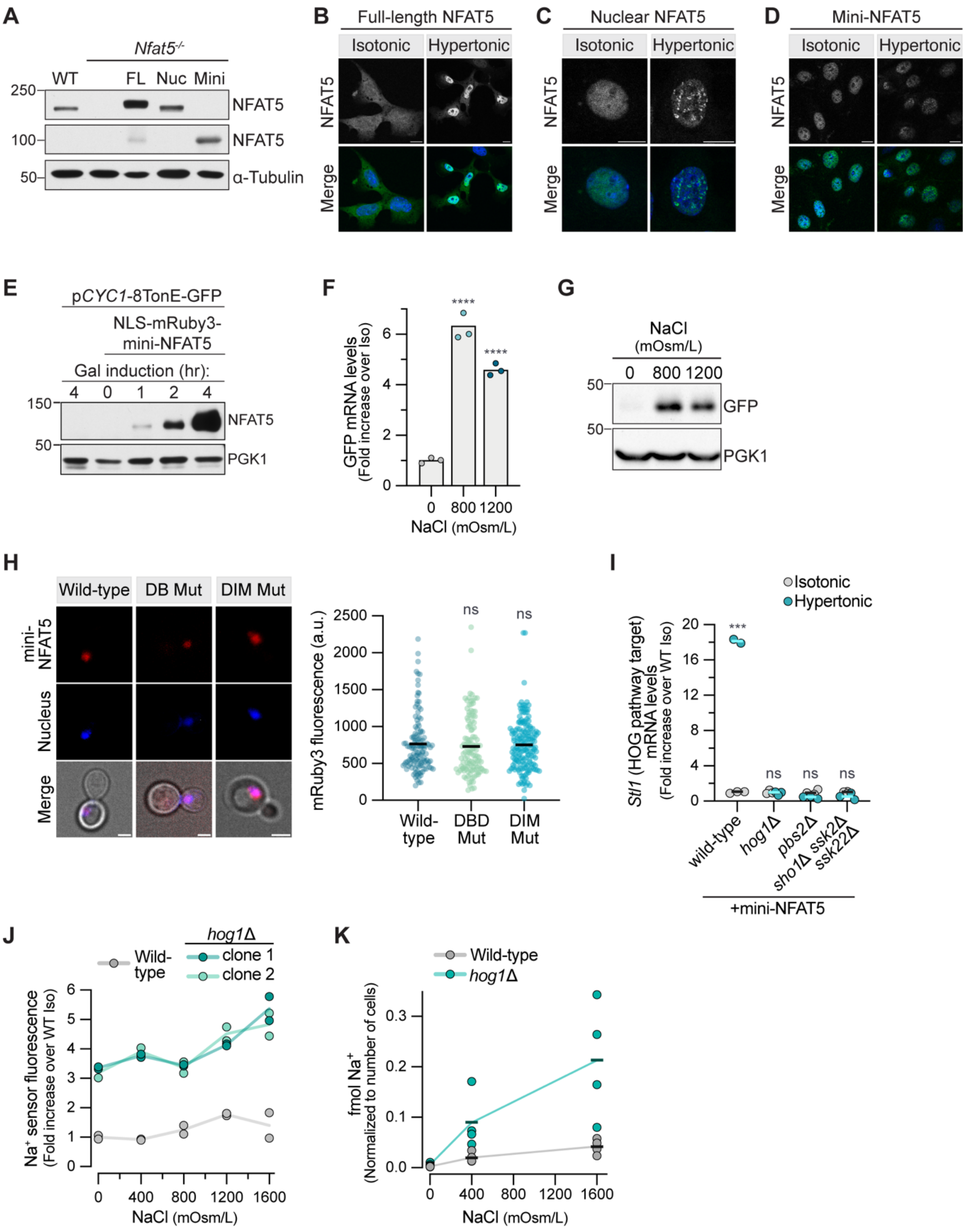

fig. S3. Characterization of mini-NFAT5 in IMCD3 and yeast cells, Related to Fig. 2.
(A-D) Abundances (**A**) and subcellular distributions (**B-D**) of mVenus-tagged full-length (FL), Nuclear (Nuc), or mini-NFAT5 stably expressed from a single defined genomic locus in *Nfat5*-/-

IMCD3 cells. **Fig. 2A** shows domain structures of these variants. (**B-D**) Cells were exposed to isotonic or hypertonic (+200 mOsm/L NaCl) media for 30 minutes (min) prior to analysis. Fluorescence signal from the mVenus tag fused to NFAT5 is shown alone (top) or merged with a DNA stain for nuclei (DAPI, bottom).
(E) Abundance of NLS-mRuby3-mini-NFAT5 at various times after galactose addition in a yeast strain used to test activation of NFAT5 by hypertonic stress. This strain contains two stably integrated transgenes, one encoding NLS-mRuby3-mini-NFAT5 driven by a galactose-inducible promoter and a second encoding the 8xTonE-*pCYC1*-GFP NFAT5 reporter (**Fig. 2D**). The leftmost lane shows a control strain lacking the mini-NFAT5 transgene but containing the reporter.
(F,G) Abundance of GFP mRNA (**F,** RT-qPCR) and protein (**G**, immunoblotting) in yeast cells expressing the 8xTonE-*pCYC1*-GFP reporter and NLS-mRuby3-mini-NFAT5 after 2 hrs (**F**) or 4 hours (**G**) in CSM containing the indicated concentrations of NaCl.
(H) Representative images (left) showing subcellular localization of wild-type NLS-mRuby3-mini-NFAT5 and variants carrying mutations that abrogate DNA binding (DB Mut) or dimerization (DIM Mut) (see **Fig. 2G**) in yeast cells used in the experiment depicted in **Fig. 2H**. Graph on right shows quantitation of nuclear mRuby3 fluorescence in single yeast cells (measured from images of the type shown on the left) expressing the indicated NFAT5 variant (n>118 cells per strain).
(I) Expression of the HOG pathway target gene *Stl1* in wild-type, *hog1*Δ, *pbs2*Δ, or *ssk*2Δ *ssk22*Δ *sho1*Δ cells after 2 hrs in CSM supplemented with 1200 mOsm/L NaCl.
(J) Fluorescence of a sodium sensor (see methods) in wild-type and *hog1*Δ cells (2 independent clones) in response to increasing amounts of NaCl added to CSM. Two technical replicates were measured for each clone and are shown separately.
(K) Abundance of sodium ions was measured by inductively coupled plasma optical emission spectroscopy (ICP-OES) in wild-type and *hog1*Δ cells.
Scale bars for panels (**B-D**): 10 μm; panel (**H**): 2 μm.
Statistics: Bars (**F**) or horizontal lines (**H**,**I**,**K**) denote mean values calculated from independent measurements shown as points. Statistical significance was determined by a one-way (**F**) or two-way (**I**) ANOVA test with Sidak's multiple comparison post-test (n>3 independent experiments) or by the Kruskal Wallis test (**H**). p-values: **** <0.0001 and *** <0.001.

Supplementary Figure S4

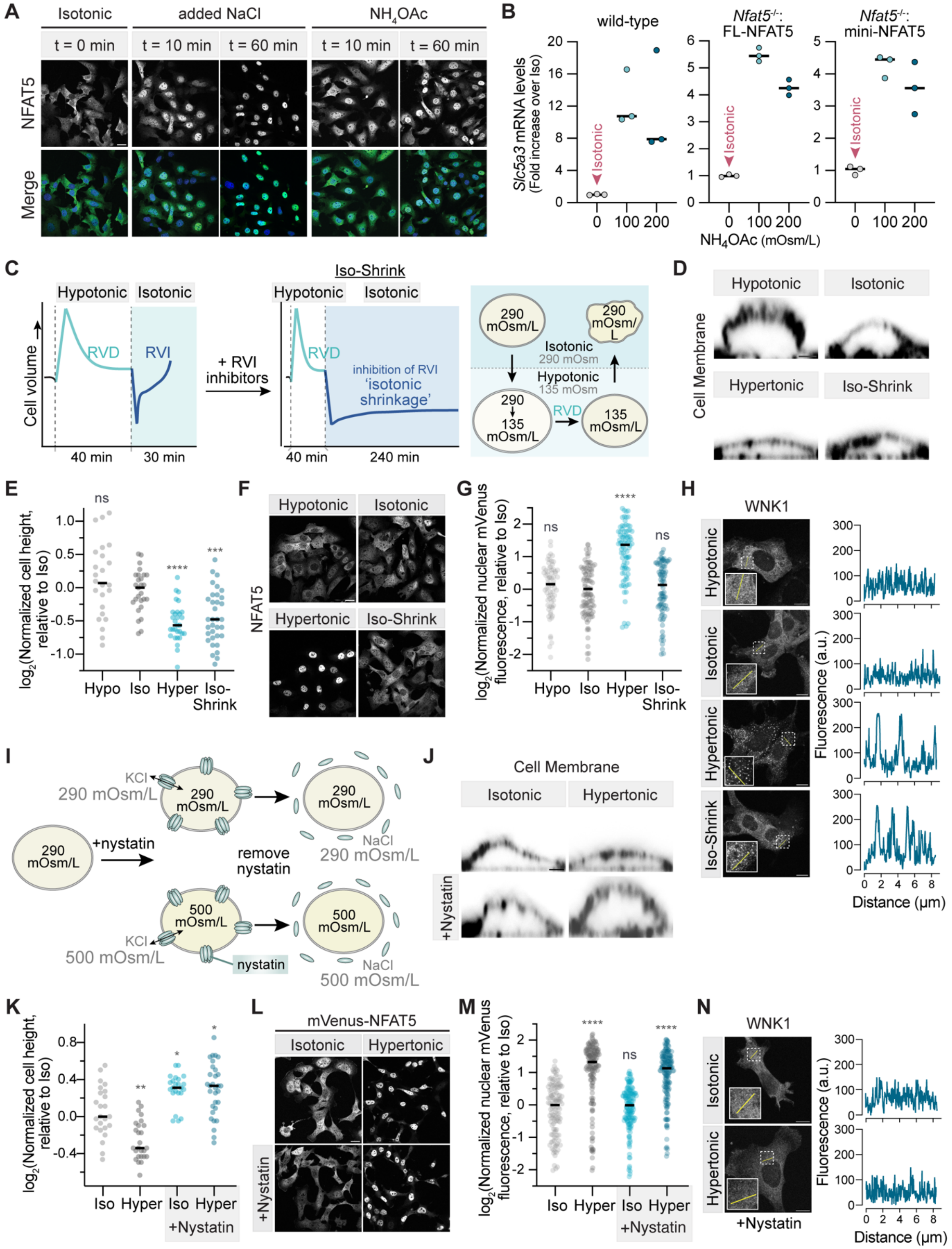

fig. S4. The effects of macromolecular crowding and ionic stress on NFAT5 and WNK1, Related to Fig. 3.
(A) The subcellular localization of stably expressed mVenus-NFAT5 (top) after 10 or 60 min in hypertonic media (+200 mOsm/L NaCl or NH_4OAc). Fluorescence signal from the mVenus tag fused to NFAT5 is shown alone (top) or merged with a nuclear stain (DAPI, bottom). Images like these were used for the quantitative analysis of NFAT5 nuclear accumulation shown in **Fig. 3H**.
(B) Expression of an NFAT5 target gene in WT IMCD3 cells or *Nfat5*$^{-/-}$ IMCD3 cells stably expressing mVenus-tagged full-length or mini-NFAT5 6 hrs after the addition of NH_4OAc to isotonic media (see **Fig. 3I** for responses measured using the 8xTonE-GFP NFAT5 reporter). Black horizontal lines mark the median of three independent measurements, each shown as a point.
(C) Diagram depicting the principle behind the isotonic shrinkage (Iso-Shrink) protocol, which allows cell shrinkage under isotonic conditions. Cells subjected to a hypotonic shock initially swell, but then restore their volume through regulatory volume decrease (RVD), which is caused by loss of osmotically active cytoplasmic ions and small molecules. The subsequent transfer of cells to isotonic media causes cell shrinkage since the cytoplasmic osmolarity is lower than media osmolarity. The addition of regulatory volume increase (RVI) inhibitors prevents cell volume recovery following isotonic shrinkage. Schematic taken from reference (*34*).
(D) Confocal images of IMCD3 cells in the xz plane stained with CellMask to highlight the plasma membrane. Cells were exposed to hypotonic (~200 mOsm/L), isotonic (~300 mOsm/L), and hypertonic (~500 mOsm/L with the addition of 200 mOsm/L NaCl) conditions, or subjected to the isotonic shrinkage protocol shown in **fig. S4C** and described in the methods.
(E) The height of IMCD3 cells (n>24 cells per condition, measured from images of the type shown in **fig. S3D**) after the isotonic shrinkage protocol (**fig. S3C)** compared to cells in hypotonic, isotonic, or hypertonic media.
(F, G) Subcellular localization of mVenus-NFAT5 stably expressed in IMCD3 cells following isotonic shrinkage, compared to cells in hypotonic, isotonic, or hypertonic media. Nuclear mVenus fluorescence measured from single cells (n>69 cells per condition) is plotted in (**G**).
(H) Distribution of GFP fluorescence in *Wnk1*$^{-/-}$ IMCD3 cells stably expressing GFP-WNK1 after isotonic shrinkage, compared to its distribution in cells exposed to hypotonic, isotonic, or hypertonic media. Line scans show fluorescence intensity traces along the trajectories of the lines shown in the insets.
(I) The reversible ionophore nystatin forms ion conducting pores in the plasma membrane that allow equilibration of intracellular and extracellular osmolarity. When nystatin-treated cells are exposed to hypertonic media, intracellular ion concentrations rise without collateral cell shrinkage and macromolecular crowding.
(J) Confocal images of IMCD3 cells in the xz plane stained with CellMask to highlight the plasma membrane. Cells were exposed to isotonic or hypertonic media in the presence or absence of nystatin.
(K) The height of IMCD3 cells (n>23 cells per condition, measured from images of the type shown in **fig. S3J**) after exposure to isotonic or hypertonic media in the presence or absence of nystatin.
(L, M) Subcellular localization of mVenus-NFAT5 in IMCD3 cells after exposure to isotonic or hypertonic media in the presence or absence of nystatin. Nuclear mVenus fluorescence measured from single cells (n>69 cells per condition) is plotted in (**M**).
(N) Subcellular distribution of GFP-WNK1 following nystatin treatment in isotonic or hypertonic media. Line scans correspond to fluorescence intensity traces along the trajectories of the lines in the inset.

Scale bars for panels (**A,F,L**): 20 μm; panels (**D,J**): 2 μm; panels (**H,N**): 10 μm.
Statistics: Points in (**E**,**G**,**K**,**M**) denote measurements from single cells and the black horizontal lines mark the median of the populations. Statistical significance (**E**,**G**,**K**,**M**) of differences in comparison to the isotonic condition was determined by a Kruskal-Wallis test with Dunn's multiple comparison test ($n>3$ independent experiments). p-values: **** <0.0001, *** <0.001, ** <0.01, and * <0.05.

Supplementary Figure S5

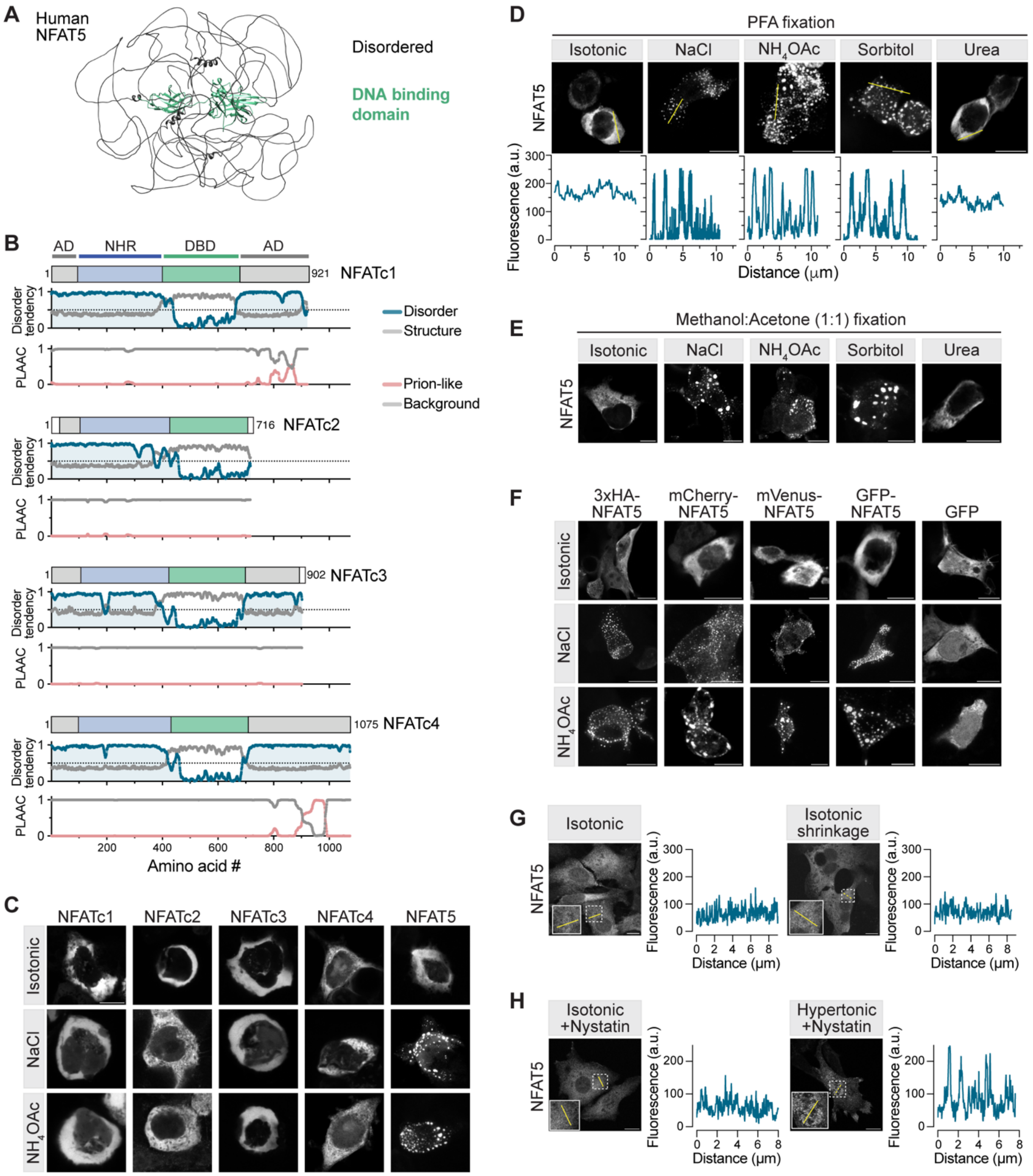

fig. S5. Formation of NFAT5 droplets in response to stress, Related to Fig. 4.
(**A**) AlphaFold prediction of the mostly disordered human NFAT5 (Uniprot ID: O94916) structure (*96*).
(**B**) Domain structures, disorder tendency and predicted prion-like regions in human NFATc1-4 (compared to NFAT5 shown in **Fig. 4A**). NFATc1-4 share a conserved NFAT-homology region

(NHR) and DNA binding domain (DBD, also conserved with NFAT5 and the Rel family of TFs) flanked by N- and C-terminal activation domains (AD).
(**C**) Distribution of GFP-tagged NFAT proteins transiently transfected into HEK293T cells 30 min after the addition of NaCl or NH_4OAc (+100 mOsm/L each) to isotonic media.
(**D,E**) Distribution of GFP-NFAT5 in transiently-transfected HEK293T cells 30 min after the addition of NaCl, NH_4OAc, sorbitol or urea (+100 mOsm/L each). Cells were fixed with paraformaldehyde (PFA) in (**D**) or Methanol/Acetone in (**E**). Line scans in (**D**) correspond to fluorescence intensity traces along the trajectories of the yellow lines on the images.
(**F**) Distribution of NFAT5 fused to different epitope tags in HEK293T cells after the addition of NaCl or NH_4OAc (+100 mOsm/L each, 30 min). Localization of GFP alone (without fusion to NFAT5) is shown in the rightmost column.
(**G,H**) Distribution of GFP-NFAT5 stably expressed in *Nfat5*$^{-/-}$ IMCD3 after isotonic shrinkage (**G**) or nystatin treatment (**H**) in the presence of an isotonic or hypertonic solution (see **figs. S4C** and **S4I** and Methods for details). Line scans correspond to fluorescence intensity traces along the trajectories of the lines shown in the insets.
Scale bars for panels (**C-H**): 10 µm.

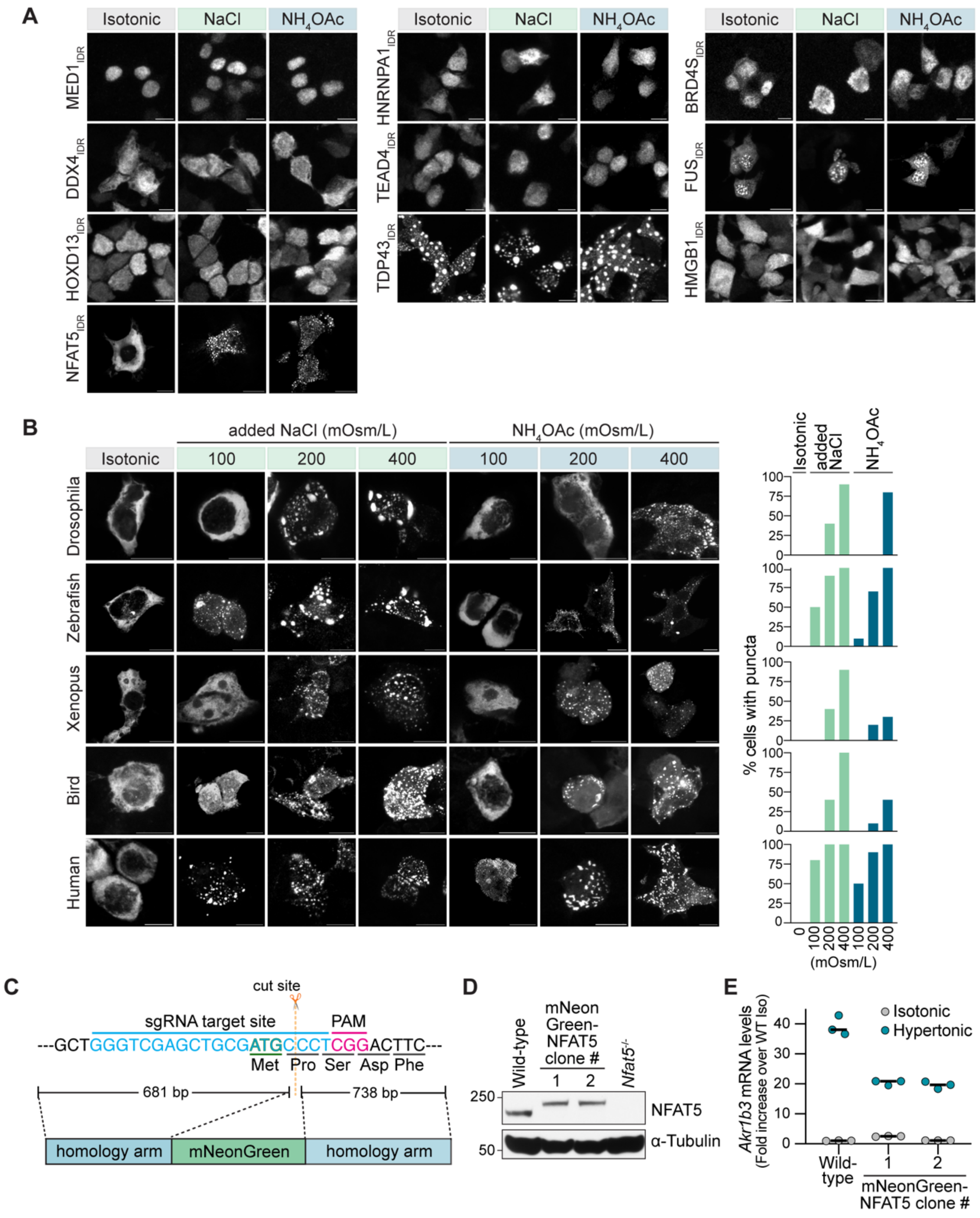

fig. S6. Evolutionarily conserved ionic stress sensing by NFAT5, Related to Fig. 4.
(**A**) Distribution of the GFP-tagged NFAT5 CTD (**Fig. 2A**) compared to Fluorescent Protein-tagged IDRs from nine other proteins after transient transfection into HEK293T cells. Cells were fixed and imaged 30 min after the addition of NaCl or NH_4OAc (+100 mOsm/L each) to isotonic media.

(**B**) Distribution of chimeric NFAT5 proteins transiently transfected into HEK293T cells. Chimeras were generated by replacing the human NFAT5 CTD (a.a. 544-1531) with corresponding CTDs from insect (*Drosophila melanogaster*, a.a. 542-1210), fish (*Danio rerio*, a.a. 534-1257), amphibian (*Xenopus laevis*, a.a. 489-1408) and bird (*Columba livia*, a.a. 496-1455) NFAT5 homologs. Cells were imaged 30 min after addition of the indicated concentrations of NaCl or NH_4OAc to isotonic media. Bar graphs on the right show the percentage of transfected cells with puncta at each salt concentration (*n*>210 cells evaluated per condition).
(**C**) Exon 1 sequence of the mouse *Nfat5* gene targeted by the sgRNA used to insert the mNeonGreen coding sequence in IMCD3 cells using homology-directed repair. The Protospacer Adjacent Motif (PAM) and site of predicted cleavage by Cas9 are shown above the sequence; the donor template for homologous recombination is shown below.
(**D**) Insertion of the mNG tag at the N-terminus of both *Nfat5* alleles in IMCD3 cells was confirmed by immunoblotting.
(**E**) Expression of an NFAT5 target gene in wild-type IMCD3 cells or two clonal *mNG-Nfat5* knock-in cell lines after 8 hrs in isotonic or hypertonic media (+200 mOsm/L NaCl). Black horizontal lines denote the median from three independent measurements shown as points.
Scale bars for panels (**A**,**B**): 10 μm.

Supplementary Figure S7

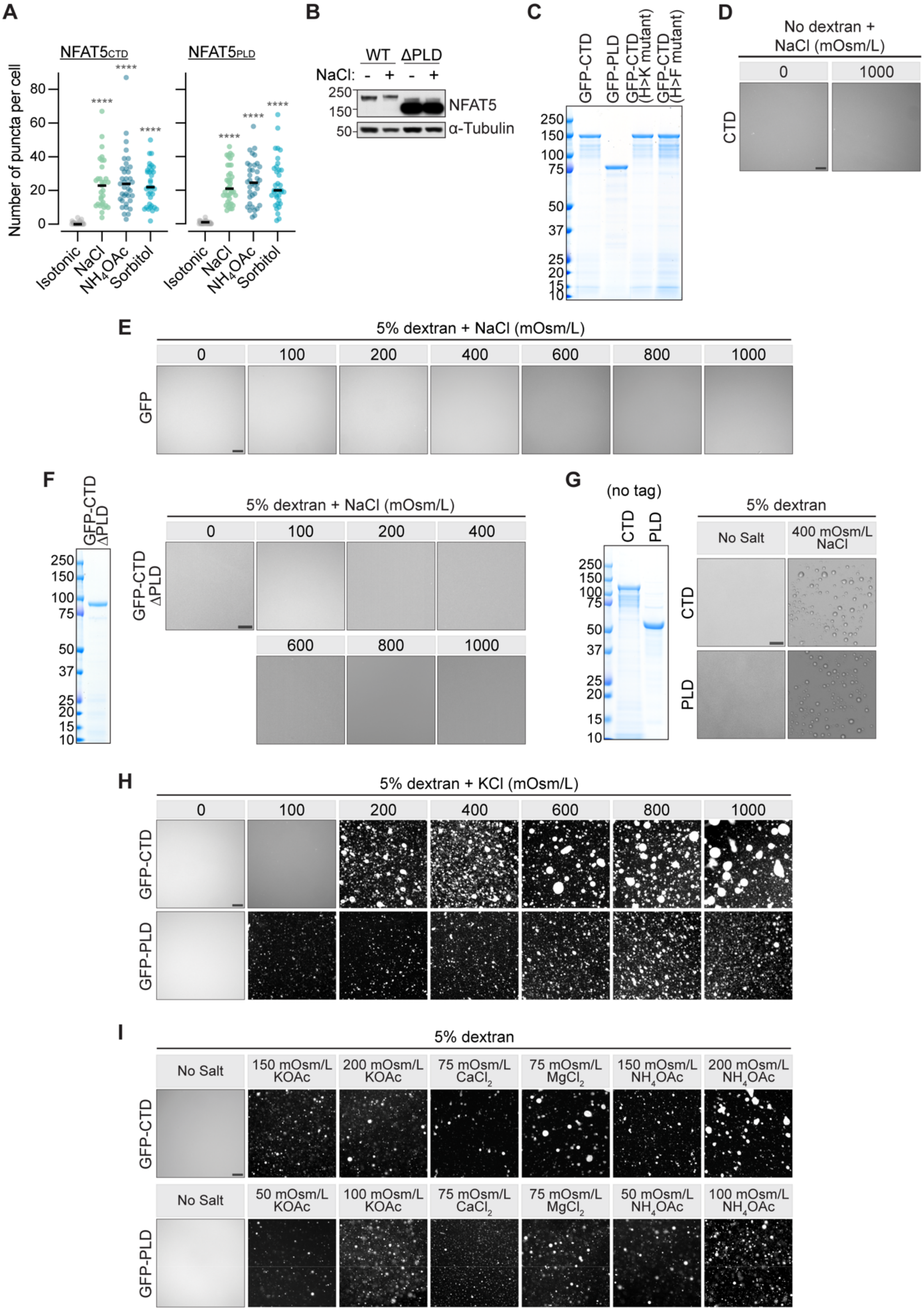

fig. S7. ***In vitro*** **and** ***in vivo*** **condensate formation by the NFAT5 CTD and PLD, Related to Fig. 5.**

(**A**) Number of puncta per cell in HEK293T cells transiently expressing GFP-CTD or GFP-PLD (see **Fig. 5A** for representative images). Black horizontal line showing the median of n>25 cells. Statistical significance of the differences in comparison to the isotonic condition was determined with a Kruskal-Wallis test and Dunn's multiple comparison test. p-value: **** <0.0001

(**B**) Abundances of GFP-NFAT5 or GFP-NFAT5 ΔPLD stably expressed in *Nfat5*$^{-/-}$ cells measured by immunoblotting. See **Fig. 5B** for domain structures.

(C,F,G) Coomassie-stained polyacrylamide gels showing the purity of proteins used for *in vitro* droplet formation assays in **Fig. 5**, **fig. S7 and fig. S9**. Molecular weight standards in kilodaltons (kDa) are indicated to the left.

(**D**) Fluorescence microscopy was used to assess droplet formation *in vitro* by purified GFP-CTD (70 µM) in buffered solution <u>without</u> Dextran.

(**E,F**) Fluorescence microscopy was used to assess droplet formation *in vitro* by 100 µM purified GFP or GFP-CTD-ΔPLD (CTD lacking the PLD, see **Fig. 5B**) at the indicated concentrations of NaCl.

(**G**) Brightfield microscopy was used to assess droplet formation by untagged NFAT5 CTD (70 µM) and PLD (90 µM).

(**H,I**) *In vitro* droplet formation by GFP-NFAT5 CTD (70 µM, top row) or GFP-NFAT5 PLD (90 µM, bottom row) at increasing concentrations of KCl (**H**) or in the presence of a variety of different salts (**I**). All solutions contained 5% dextran.

Scale bars for panels (**D-I**): 5 µm.

Supplementary Figure S8

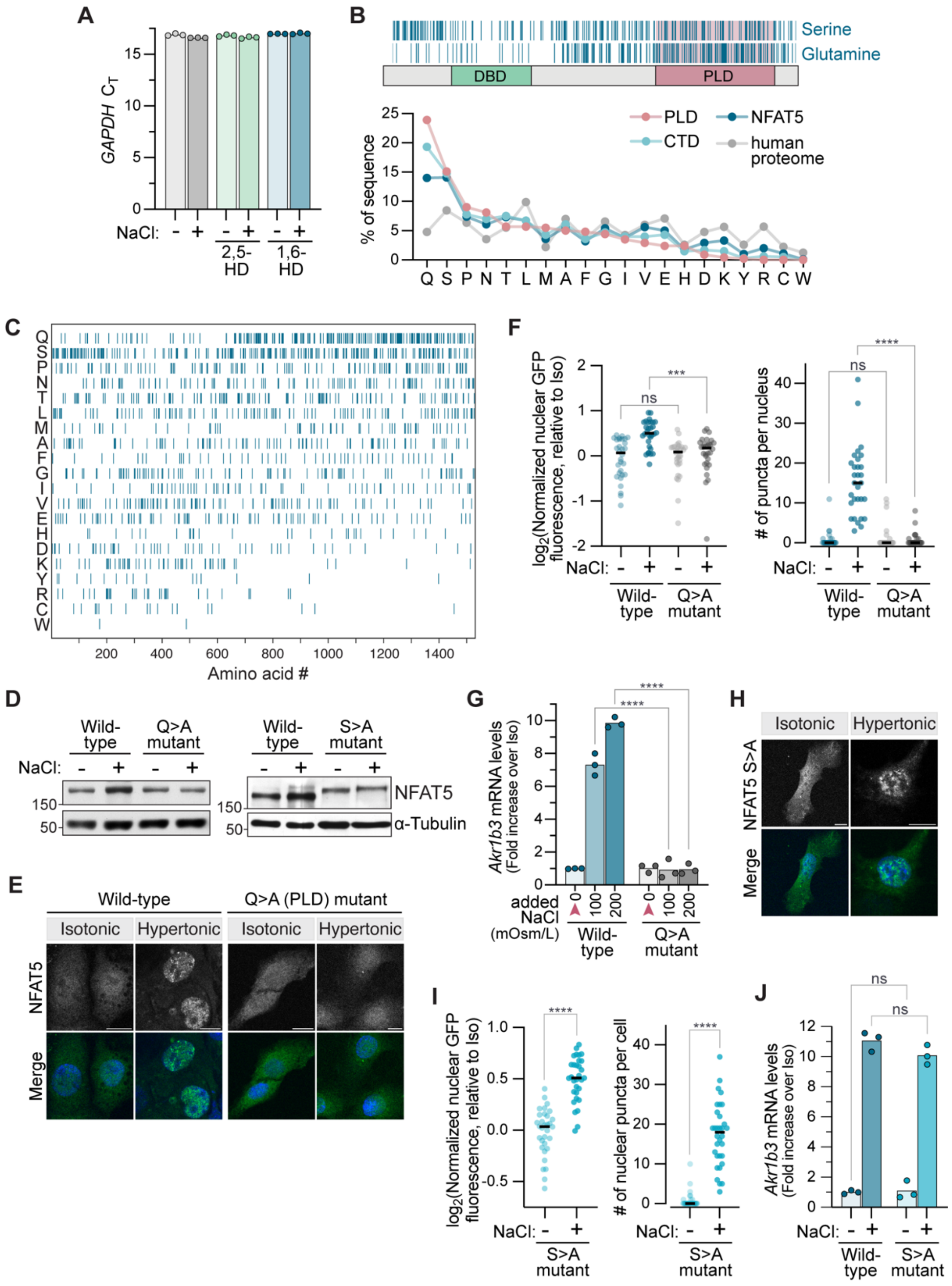

fig. S8. Compositional mutagenesis of NFAT5, related to Fig. 6.

(A) Abundance of *Gapdh* mRNA (measured by its C_T value in a RT-qPCR assays) after 10 hrs of exposure to 1% 1,6-HD or 2,5-HD in isotonic or hypertonic (+200 mOsm/L NaCl) media.

Compare to the effect of 1,6-HD and 2,5-HD on *Akr1b3* mRNA abundance in **Fig. 6B**. Bars show the mean of 3 measurements.

(B) Domain structure of NFAT5 with the positions of serine and glutamine residues marked with vertical blue lines (top). All 69 serine or 109 glutamine residues in the PLD (highlighted in red) were mutated to alanine in the GFP-NFAT5_SA or GFP-NFAT5_QA variants, respectively. Amino acid composition in NFAT5 or its isolated CTD and PLD fragments compared to the global composition in the human proteome (bottom).

(C) Position of each of the 20 amino acids in the NFAT5 linear sequence (oriented along the x-axis). In each row, every occurrence of a single amino acid is marked by a vertical blue line.

(D) Immunoblot comparing the abundances of WT GFP-NFAT5 to the GFP-NFAT5_QA and GFP-NFAT5_SA mutants.

(**E,F**) Subcellular distribution (**E**) of full-length GFP-NFAT5 or the GFP-NFAT5_QA mutant (**B**) stably expressed in *Nfat5*$^{-/-}$ IMCD3 cells after the addition of NaCl (+200 mOsm/L, 30 min) to isotonic media. Fluorescence signal from NFAT5 is shown alone (top) and merged with DAPI signal to show nuclei (bottom). Images of the type shown in (**E**) were used in (**F**) to measure nuclear NFAT5 fluorescence (left) and number of NFAT5 nuclear puncta (right) per cell (n>30 cells, with black horizontal line showing the median).

(**G**) Expression of an NFAT5 target gene in cells expressing the indicated NFAT5 variants following the addition of NaCl to isotonic media (red arrowhead) for 8 hrs.

(**H,I**) Subcellular distribution of GFP-NFAT5_SA stably expressed in *Nfat5*$^{-/-}$ IMCD3 cells after 30 min in hypertonic media (+200 mOsm/L NaCl). Fluorescence signal from NFAT5 is shown alone (top) and merged with DAPI to mark nuclei (bottom). Images of the type shown in (**H**) were used in (**I**) to measure nuclear NFAT5 fluorescence (left) and number of NFAT5 nuclear puncta (right) per cell (n>30 cells, with black horizontal line showing the median).

(J) Expression of an NFAT5 target gene in cells expressing the indicated NFAT5 variants after 8 hrs in isotonic or hypertonic (+200 mOsm/L NaCl) media. Bars denote the mean of 3 measurements.

Scale bars for panels (**E,H**): 10 µm

Statistics: For (**A,G,J**), statistical significance was determined by a two-way ANOVA test, Sidak's multiple comparison. For (**F,I**) statistical significance was determined by a Kruskal-Wallis test, Dunn's multiple comparison. p-values: **** <0.0001 and *** <0.001.

Supplementary Figure S9

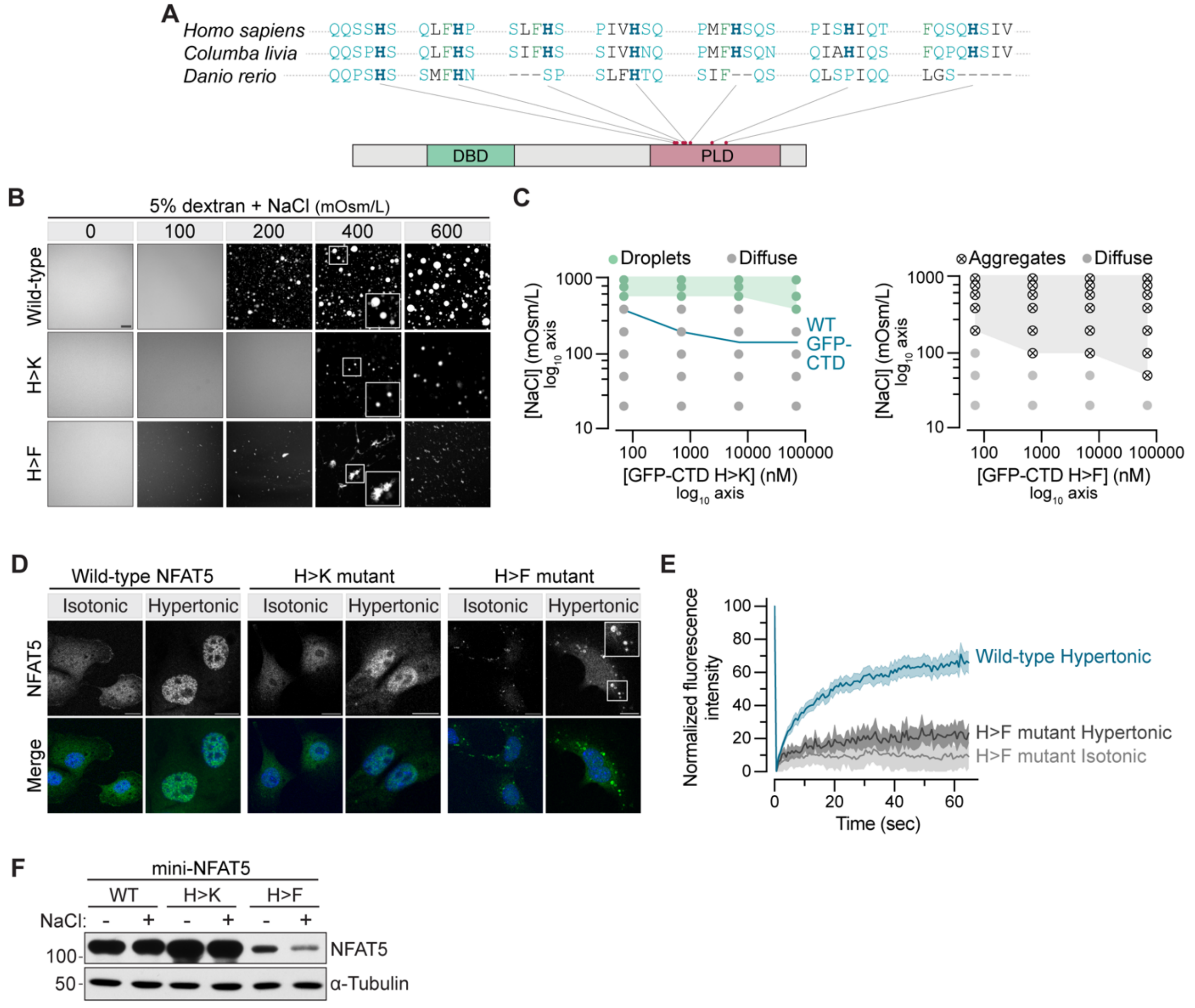

fig. S9. Point mutations in NFAT5 alter condensation propensity, related to Fig. 6.

(**A**) Domain structure of NFAT5 with the positions of the seven histidines within the PLD targeted for mutagenesis marked with red dots. Cross-species sequence conservation of these histidines is shown (top).

(**B**) *In vitro* droplet formation propensity of purified (**fig. S7C**) GFP-CTD (70 µM, top row), GFP-CTD_HK (70 µM) and GFP-CTD_HF (70 µM) at the indicated NaCl concentrations. The seven histidines in the NFAT5 PLD highlighted in (**A**) were mutated to lysines or phenylalanines in GFP-CTD_HK and GFP-CTD_HF, respectively. Magnified insets show condensate shape (uniformly spherical for GFP-CTD and GFP-CTD_HK and both irregular and polymorphic for GFP-CTD_HF).

(**C**) Phase diagrams for purified GFP-CTD_HK (left) and GFP-CTD_HF (right) (see **Fig. 5H** for description). Shaded areas mark conditions where GFP-CTD_HK (green shading, left) or GFP-CTD_HF (gray shading, right) form droplets or aggregates, respectively. The blue trace indicates the position of the boundary between diffuse and droplet phases of WT GFP-CTD (from **Fig. 5H**). Images were obtained across 3 biological replicates per condition.

(**D**) Subcellular distribution of full-length GFP-NFAT5 or the corresponding HK and HF mutants stably expressed from a single locus in *Nfat5*$^{-/-}$ IMCD3 cells after the addition of NaCl (+200 mOsm/L, 30 min) to isotonic media. Fluorescence signal from NFAT5 is shown alone (top) and merged with DAPI to mark nuclei (bottom). Magnified insets show the irregular morphology of condensates formed by NFAT5_HF.
(**E**) FRAP curves (*n*=14; mean ± SEM) of full-length GFP-NFAT5 or GFP-NFAT5_HF condensates in IMCD3 cells subjected to hypertonic stress (+200 mOsm/L NaCl, 30 min).
(**F**) Immunoblot comparing the abundances of WT mini-NFAT5, mini-NFAT5_HK and mini-NFAT5_HF analyzed in **Fig. 6**.
Scale bars for panel (**B**): 5 µm; (**D**): 10 µm.

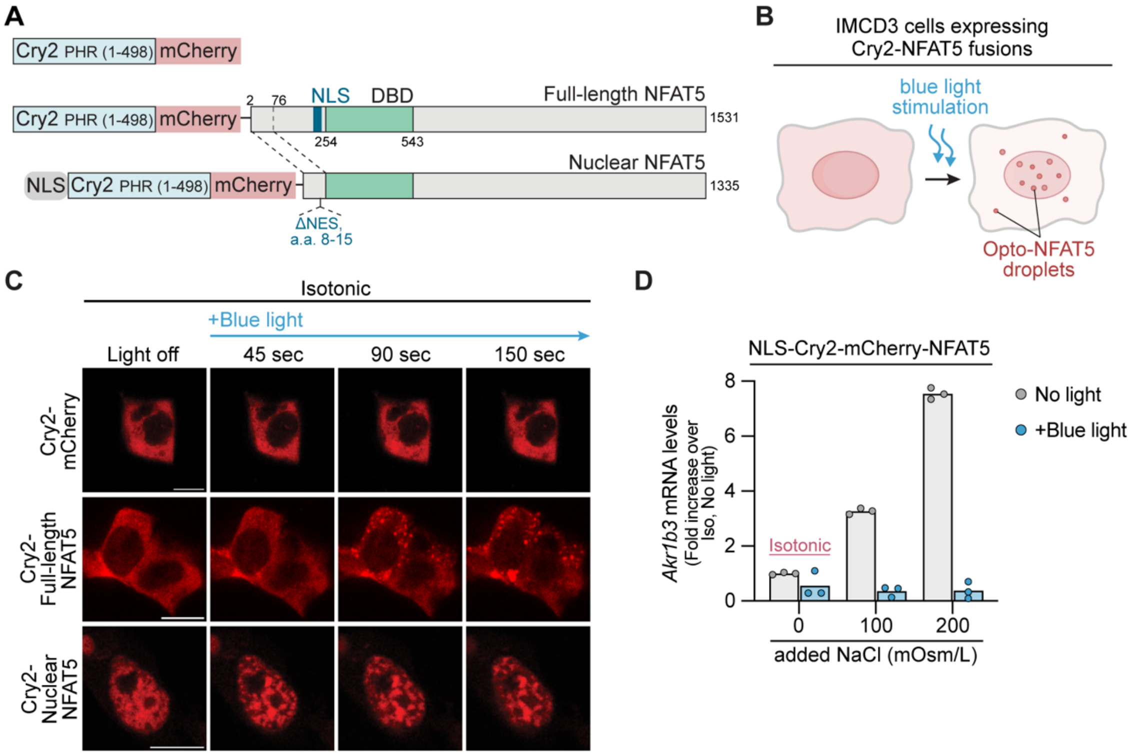

fig. S10. Light-induced NFAT5 condensation is insufficient to activate transcription, related to Fig.7.

(**A**) Domain structures of human full-length and Nuclear NFAT5 (**Fig. 2A**) fused to the Cry2 photolyase homology region (PHR) domain and mCherry. Nuclear NFAT5 is constitutively targeted to the nucleus.

(**B**) Blue light illumination promotes Cry2 self-association, increasing the local concentration of NFAT5 and thereby triggering the condensation of Cry2-NFAT5 into optodroplets.

(**C**) Snapshots from a live-cell time course following blue light (488 nm) illumination of cells expressing Cry2, Cry2-NFAT5 and Cry2-Nuclear NFAT5. Unlike Cry2 alone, Cry2-NFAT5 and Cry2-Nuclear NFAT5 form condensates in the cytoplasm and nucleus, respectively. Scale bars: 10 µm.

(D) Expression of an NFAT5 target gene in *Nfat5*$^{-/-}$ IMCD3 cells stably expressing Cry2-Nuclear NFAT5 after exposure to hypertonic stress (+100 or 200 mOsm/L NaCl) in the absence or presence of blue light illumination. Bars denote the mean of 3 measurements, each shown as points.

Supplementary Figure S11

A

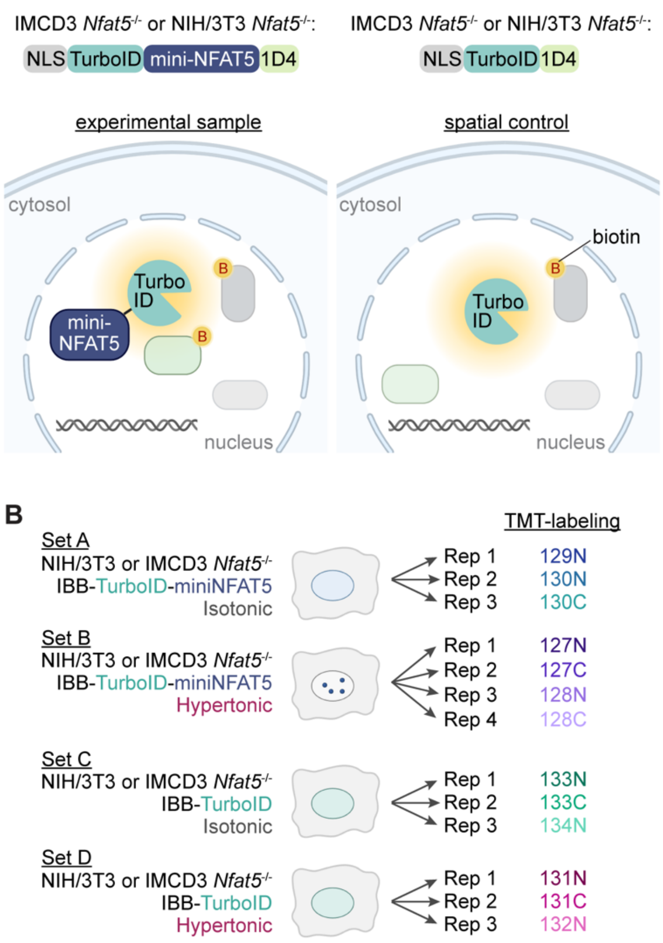

C

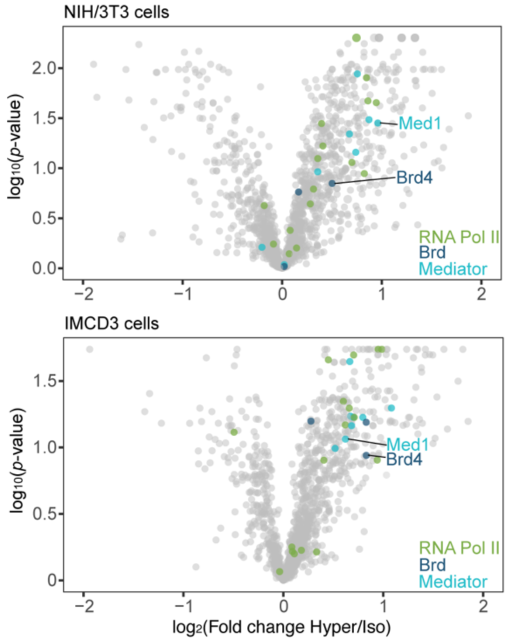

D

Enriched Gene Sets	NES	FDR q-val
GO MF Transcription coactivator activity	1.98	0.000
GO CC Spliceosomal complex	1.94	0.000
GO BP mRNA processing	1.94	0.000
GO BP RNA splicing	1.94	0.000
GO BP mRNA metabolic process	1.91	0.000
GO BP Positive regulation of DNA templated transcription	1.87	0.000
GO BP RNA 3 end processing	1.86	0.001
GO CC RNA U2 type spliceosomal complex	1.85	0.001
GO BP Regulation of DNA templated transcription initiation	1.83	0.002
GO CC Nuclear protein containing complex	1.82	0.002
GO CC SAGA type complex	1.79	0.004
GO CC Histone acetyltransferase complex	1.78	0.005
GO CC RNA polymerase II holoenzyme	1.77	0.005
GO MF Transcription coregulator activity	1.77	0.005
GO BP Cellular response to external stimulus	1.75	0.007
GO BP Regulation of stem cell population maintenance	1.75	0.007

E

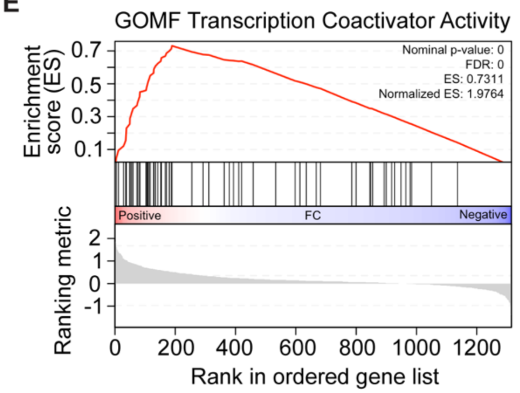

F

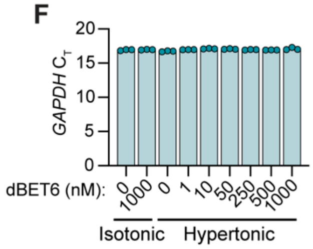

G

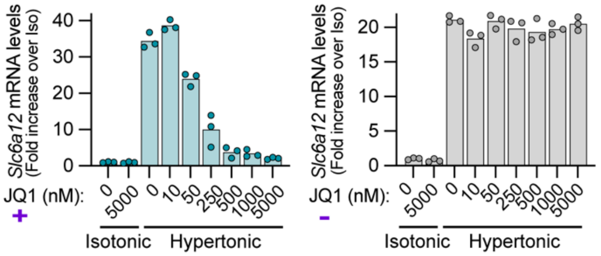

fig. S11. Proximity biotinylation with TurboID to map the mini-NFAT5 interaction landscape, related to Fig.7.

(**A)** Proximity biotinylation strategy to identify specific mini-NFAT5 interaction partners in IMCD3 and mouse NIH/3T3 cells (*71*). Mini-NFAT5 fused to TurboID (left) or TurboID alone (right), both targeted to the nucleus with a foreign Nuclear Localization Signal (NLS), were stably expressed in *Nfat5*$^{-/-}$ cells. NLS-TurboID served as a spatial control and was used to identify and exclude nuclear proteins biotinylated by TurboID irrespective of fusion to mini-NFAT5.

(B) Tandem mass tags (TMT) added to peptides from each of the samples in a 13-plex mass spectrometry experiment (performed separately for NIH/3T3 and IMCD3 cells). At least 3 biological replicates (Rep) were used for each condition. Cells were exposed to 100 µM biotin in isotonic or hypertonic (+200 mOsm/L NaCl) media for 2 hours prior to processing for streptavidin affinity chromatography and mass spectrometry.

(C) Volcano plots showing mini-NFAT5 proximal proteins enriched or depleted under hypertonic conditions in NIH/3T3 (top) or IMCD3 (bottom) cells. The x-axis shows the enrichment of each protein in the hypertonic sample relative to the isotonic sample and the y-axis shows statistical significance as measured by the $\log_{10} p$ value.

(**D**) Gene set enrichment analysis (GSEA) against all available Gene Ontology (GO) lists (*92*). The input for GSEA was a gene list ranked based on fold-enrichment in the hypertonic sample over the isotonic sample (see **C**). The table lists the top gene sets enriched in our ranked gene list, along with a Normalized Enrichment Score (NES) and False Discovery Rate (FDR) corrected *p*-value for each set.

(**E**). GSEA enrichment plot for the top most enriched gene set: GO Molecular Function (MF) transcription coactivator activity gene (see **D**). Genes in this set enriched in the hypertonic sample are positively correlated and those enriched in the isotonic sample negatively correlated. Vertical black lines denote the position of individual genes in the transcription_coactivator_activity set in our ranked list.

(**F**) Expression of a constitutively transcribed gene (*Gapdh*) is unaffected by treatment of cells by dBET6.

(**G)** Expression of an NFAT5 target gene in WT IMCD3 cells after 8 hrs in isotonic or hypertonic media (+200 mOsm/L NaCl) in the presence of a BRD4 inhibitor (+)-JQ1 or its inactive enantiomer (-)-JQ1. Bars denote the mean of 3 measurements and the experiment was repeated 3 times.

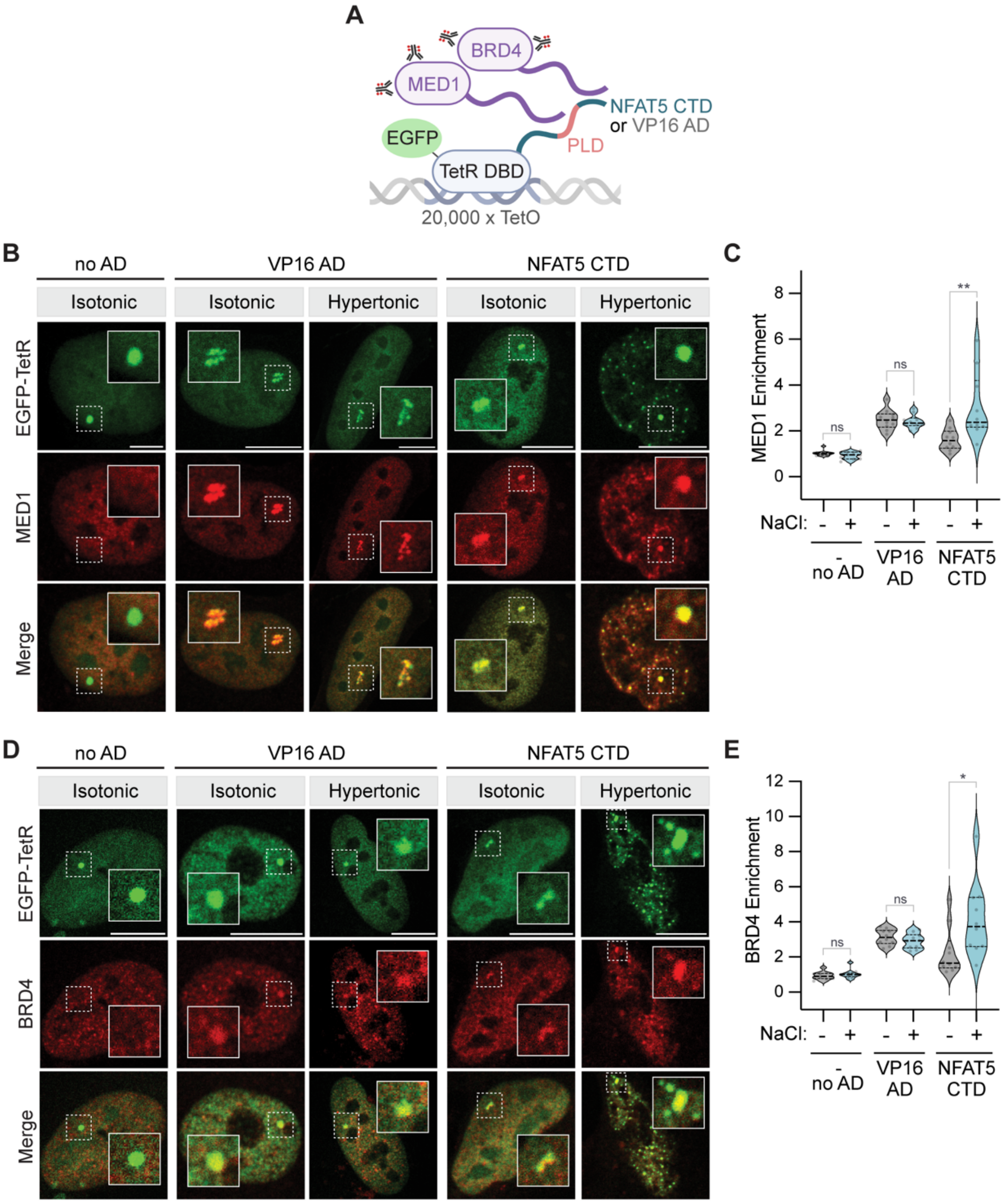

fig. S12. Co-activator recruitment by the NFAT5 CTD, related to Fig. 8.
(**A**) Schematic of the Tet Operator (TetO) array assay (73). To test its ability to recruit endogenous MED1 and BRD4 by immunofluorescence in cells, the NFAT5 CTD was tethered to a synthetic array of ~20,000 Tet repressor (TetR) binding sites integrated into a single genomic locus in U2OS cells using EGFP-TetR DBD.
(**B**,**D**) Recruitment of endogenous MED1 (**B**) or endogenous BRD4 (**D**) by the NFAT5 CTD to the TetO array after 1 hr in isotonic or hypertonic (+200 mOsm/L NaCl) media. The AD of VP16, a constitutive transcriptional activator, was used as a control. Insets show a magnified view of the TetO array, visualized as a single dot of EGFP fluorescence (see **A**). Scale bars: 10 µm.

(**C**,**E**) Images of the type shown in **B** and **D** were used to calculate the enrichment of MED1 (**C**) or BRD4 (**E**) at the EGFP-marked TetO array. Each point represents a single cell and horizontal lines indicating the mean. Statistical significance was determined by a Kruskal-Wallis test, Dunn's multiple comparison. *p*-values: ** <0.01 and * <0.05.

Supplementary Figure S13

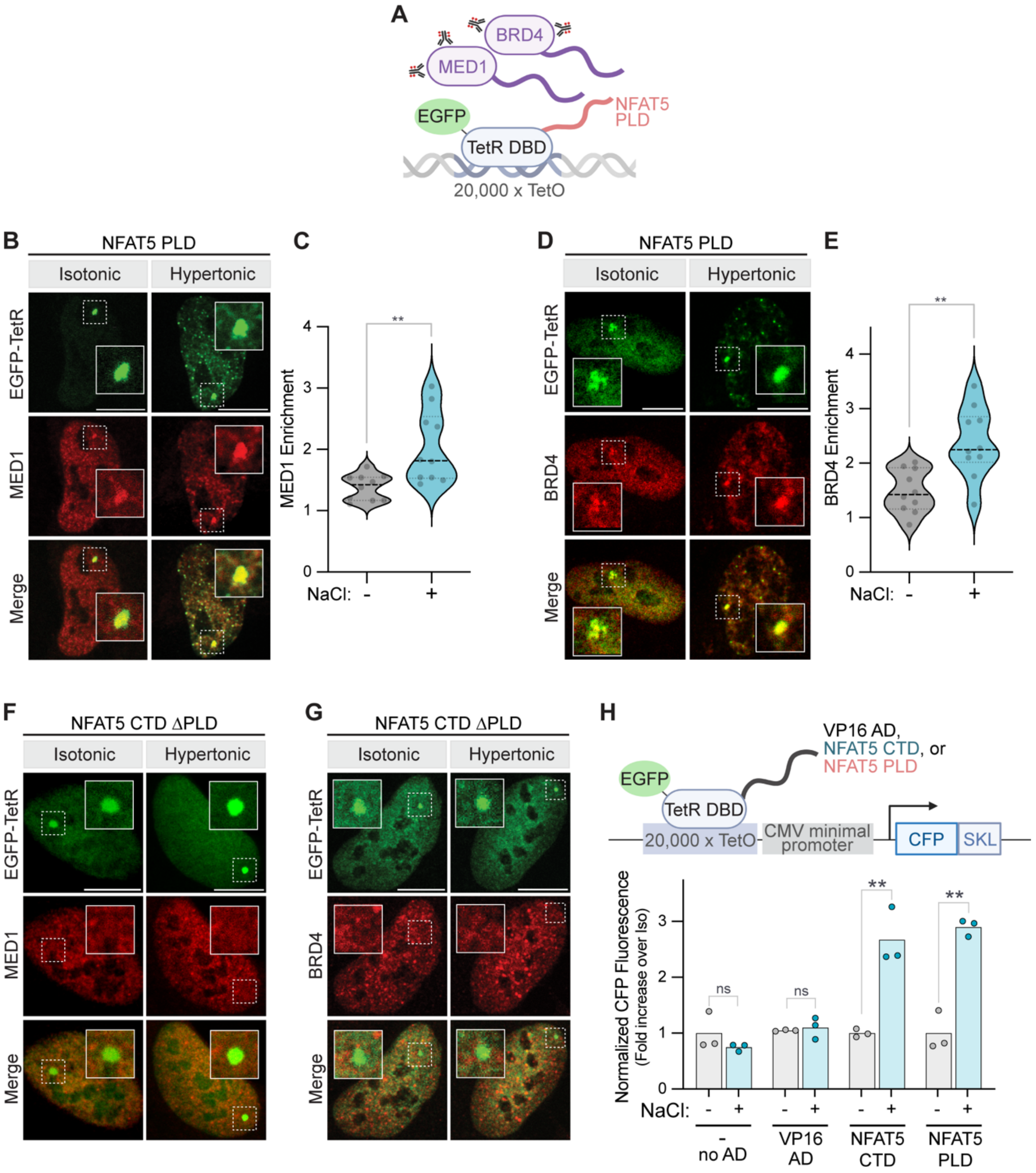

fig. S13. Co-activator recruitment by the NFAT5 PLD, related to Fig. 8.

(**A**) Schematic of the Tet Operator (TetO) array assay. To test its ability to recruit endogenous MED1 and BRD4 by immunofluorescence in cells, the NFAT5 PLD was tethered to a synthetic array of ~20,000 Tet repressor (TetR) binding sites integrated into a single genomic locus in U2OS cells using EGFP-TetR DBD.

(**B**,**D**) Recruitment of endogenous MED1 (**B**) or endogenous BRD4 (**D**) by the NFAT5 PLD to the TetO array after 1 hr in isotonic or hypertonic (+200 mOsm/L NaCl) media. Insets show a magnified view of the TetO array, visualized as a single dot of EGFP fluorescence (see **A**).

(**C**,**E**) Images of the type shown in **B** and **D** were used to calculate the enrichment of MED1 (**C**) or BRD4 (**E**) at the EGFP-marked TetO array. Each point represents a single cell and horizontal lines indicating the mean.
(**F**,**G**) Recruitment of endogenous MED1 (**F**) or endogenous BRD4 (**G**) to a TetO array in U2OS cells by EGFP-TetR DBD (green) fused to the NFAT5 CTD lacking the PLD (NFAT5 CTD ΔPLD) after 1 hr in isotonic or hypertonic (+200 mOsm/L NaCl) media. Insets show a magnified view of the TetO array, visualized as a single dot of EGFP fluorescence.
(**H**) The TetO array in U2OS cells is integrated upstream of a Cyan Fluorescent Protein (CFP) reporter driven by a minimal promoter (top). VP16 AD, NFAT5 CTD or NFAT5 PLD were tested for their abilities to activate this CFP reporter in response to hypertonic stress (+200 mOsm/L) by tethering them to the TetO array as fusions to EGFP-TetR DBD. Bars denote mean values calculated from >3 independent measurements.
Scale bars for panels (**B,D,F,G**): 10 µm.
Statistics: For (**C,E,H**), statistical significance was determined by a Mann-Whitney test. *p*-values: ** <0.01.

Table S1: Reagents and resources used in this study.

Reagent or resource	Source	Identifier
**Antibodies**		
Rabbit polyclonal anti-NFAT5	Bethyl Laboratories	Cat# A305174AT; RRID: AB_2631567
Mouse monoclonal anti-Alpha Tubulin (clone DM1A)	Sigma-Aldrich	Cat# T6199; RRID: AB_477583
Mouse monoclonal anti-1D4	The University of British Columbia	RRID: AB_325050
Rabbit polyclonal anti-GFP	Novus Biologicals	Cat# NB600-308; RRID: AB_10003058
Mouse monoclonal anti-HA (clone 2-2.2.14)	Thermo Fisher Scientific (Invitrogen)	Cat# 26183; RRID: AB_10978021
Rabbit polyclonal anti-p38	Abcam	Cat# ab7952; RRID: AB_306166
Mouse monoclonal anti-PGK1 (clone 22C5D8)	Thermo Fisher Scientific (Invitrogen)	Cat# 459250; RRID: AB_2532235
Sheep polyclonal anti-Phosphorylated SPAK	Gift from Dario Alessi (University of Dundee)	N/A
Sheep polyclonal anti-SPAK	Gift from Dario Alessi (University of Dundee)	N/A
Peroxidase AffiniPure Donkey Anti-Mouse IgG (H+L)	Jackson ImmunoResearch Laboratories	Cat# 715-035-150; RRID: AB_2340770
Peroxidase AffiniPure Donkey Anti-Rabbit IgG (H+L)	Jackson ImmunoResearch Laboratories	Cat# 111-035-144; RRID: AB_2307391
Peroxidase AffiniPure Donkey Anti-Sheep IgG (H+L)	Jackson ImmunoResearch Laboratories	Cat# 713-035-003; RRID: AB_2340709
Donkey anti-Rabbit IgG (H+L) Highly Cross-Adsorbed Secondary Antibody, Alexa Flour 488	Thermo Fisher Scientific	Cat# A-21206; RRID: AB_2535792
MED1 Recombinant Rabbit Monoclonal Antibody (BLR037F)	Bethyl Laboratories	Cat # A700-037; RRID: AB_2891837
BRD4 Recombinant Rabbit Monoclonal Antibody (BL-149-2H5)	Thermo Fisher Scientific (Invitrogen)	Cat # MA5-44203; RRID: AB_2926333
Anti-RNA polymerase II Antibody (clone CTD4H8)	Sigma-Aldrich	Cat# 05-623, RRID: AB_309852
Streptavidin-Horseradish Peroxidase (HRP) Conjugate	Invitrogen	Cat# SA10001
**Bacterial and virus strains**		
Rosetta2(DE3)pLysS competent cells	MilliporeSigma (Novagen)	Cat# 71403
**Chemicals, peptides, and recombinant proteins**		
Puromycin dihydrochloride	MilliporeSigma	Cat# P8833
Polyethylenimine (PEI), Linear, MW 25000, Transfection Grade	Polysciences, Inc	Cat# 23966-1
Polybrene (Hexadimethrine bromide)	MilliporeSigma	Cat# 107689
Hygromycin B (50 mg/ml in solution, Ultra Pure Grade)	VWR Life Science	Cat# 97064-454
Blasticidin S HCl, powder	Thermo Fisher Scientific (Gibco)	Cat# R21001

Gateway LR Clonase II enzyme	Thermo Fisher Scientific (Invitrogen)	Cat# 11791020
Gibson Assembly Master Mix	New England Biolabs	Cat# E2611
GoTaq Green Master Mix	Promega Corporation	Cat# M712
Penicillin:Streptomycin solution	Gemini Bioproducts	Cat# 400-109
SIGMAFAST protease inhibitor tablets	MilliporeSigma	Cat# S8820
PhosSTOP	MilliporeSigma	Cat# PHOSS-RO
X-tremeGENE 9 DNA transfection reagent	Roche	Cat# XTG9-RO
X-tremeGENE 9 HP DNA transfection reagent	Roche	Cat# XTGHP-RO
Bovine serum albumin	MilliporeSigma	Cat# A3059
Dynabeads Protein A for immunoprecipitation	Thermo Fisher Scientific (Invitrogen)	Cat# 10002D
NuPAGE-LDS sample buffer (4X)	Thermo Fisher Scientific (Invitrogen)	Cat# NP0007
TRIzol reagent	Thermo Fisher Scientific (Invitrogen)	Cat# 15596026
Doramapimod, Free Base, >99%	LC Labs	Cat# D-2744
SB 202190, Free Base, >99%	LC Labs	Cat# S-1700
CellMask^{TM} Plasma Membrane Stains	Thermo Fisher Scientific (Invitrogen)	Cat# C10046
Nystatin	Sigma-Aldrich	Cat# N4014
Gadolinium(III) chloride	Sigma-Aldrich	Cat# 439770
Bumetanide	Sigma-Aldrich	Cat# B3023
Benzamil hydrochloride hydrate	Sigma-Aldrich	Cat# B2417
Gibco Trypsin-EDTA (0.05%), phenol red	Thermo Fisher Scientific (Gibco)	Cat# 25-300-120
Opti-MEM reduced serum medium	Thermo Fisher Scientific (Gibco)	Cat #3985062
DMEM/F12 1:1 medium with L-glutamine, HEPES (Hyclone)	Cytiva	Cat# SH30023FS
Dulbecco's Modified Eagles Medium (DMEM) high glucose without L-glutamine and sodium pyruvate (Hyclone)	Cytiva	Cat# SH30081FS
IMDM modified medium with L-glutamine, HEPES (Hyclone)	Cytiva	Cat# SH30228FS
Fetal bovine serum	Sigma-Aldrich	Cat# S11150
L-glutamine solution	Gemini Bioproducts	Cat# 400-106
Sodium pyruvate	Thermo Fisher Scientific (Gibco)	Cat# 11360070
MEM Non-essential amino acids solution	Thermo Fisher Scientific (Gibco)	Cat# 11140050
MEM Non-Essential Amino Acids Solution (100X)	Thermo Fisher Scientific (Gibco)	Cat# 11140050
MEM Vitamin Solution (100X)	Thermo Fisher Scientific (Gibco)	Cat# 11120052
HT Supplement (100x)	Thermo Fisher Scientific (Gibco)	Cat# 11067030
Glucose Solution	Thermo Fisher Scientific (Gibco)	Cat# A2494001

HEPES (1 M)	Thermo Fisher Scientific (Gibco)	Cat# 15630080
Y-PER Yeast Protein Extraction Reagent	Thermo Fisher Scientific	Cat# 78991
B-PER Complete Bacterial Protein Extraction Reagent	Thermo Fisher Scientific	Cat# 89821
CSM Powder	Sunrise Science Products	Cat# 1001-010
CSM -Trp Powder	Sunrise Science Products	Cat# 1007-010
CSM -Ura Powder	Sunrise Science Products	Cat# 1004-010
CSM -Leu -Ura Powder	Sunrise Science Products	Cat# 1038-010
G-418 Solution	Roche	Cat# G418-RO
ING-1 AM Sodium Indicator	ION Biosciences	Cat# 2015G
1,6-hexanediol	BroadPharm	Cat# BP-21412
2,5-hexanediol	Sigma-Aldrich	Cat# H11904
(+)-JQ1	Cayman Chemical	Cat# 11187
(-)-JQ1	Cayman Chemical	Cat# 11232
Dextran	Sigma-Aldrich	Cat# 31392
Fluorescein Diacetate	Invitrogen	Cat# F1303
Amicon Ultra-15 Centrifugal Filters	MilliporeSigma	Cat# UFC903024
Pierce™ Streptavidin Magnetic Beads	Thermo Scientific	Cat #88816
**Critical commercial assays**		
iScript reverse transcription supermix for RT-qPCR	Bio-rad Laboratories	Cat# 1708840
Pierce BCA Protein Assay Kit	Thermo Fisher Scientific	Cat# 23227
Dual-Luciferase Reporter Assay System	Promega	Cat# E1960

Table S2: Experimental models used in this study.

Cell Line	Source
IMCD3-Flp In	Gift from Maxence Nachury (UCSF)
293FT	Thermo Fisher Scientific (Cat# R7000)
293T	ATCC (Cat# CRL-3216)
U2OS 2-6-3	Gift from David L. Spector (Cold Spring Harbor Laboratory)
IMCD3 Flp-In *Nfat5*$^{-/-}$	This paper
IMCD3 8TonE-GFP	This paper
IMCD3 8TonE-GFP + iCas9	This paper
HAP1	Gift from Thijn Brummelkamp (Netherlands Cancer Institute)
HAP1 8TonE-GFP	This paper
IMCD3 *Nfat5*$^{-/-}$ + FLAG-mVenus-NFAT5	This paper
IMCD3 *Nfat5*$^{-/-}$ + GFP-NFAT5	This paper
IMCD3 *Nfat5*$^{-/-}$ + IBB-mVenus-NFAT5	This paper
IMCD3 *Nfat5*$^{-/-}$ + IBB-mVenus-RHD-MD1-AD2 ("mini-NFAT5")	This paper
IMCD3 Flp-In *Wnk1*$^{-/-}$	This paper
IMCD3 *Wnk1*$^{-/-}$ + GFP-WNK1	This paper
IMCD3 *Nfat5*$^{-/-}$ + GFP-NFAT5 ΔPLD	This paper
IMCD3 Flp-In mNeonGreen-Nfat5	This paper
IMCD3 *Nfat5*$^{-/-}$ + GFP-NFAT5 (Q>A Mutant)	This paper
IMCD3 *Nfat5*$^{-/-}$ + GFP-NFAT5 (S>A Mutant)	This paper
IMCD3 *Nfat5*$^{-/-}$ + GFP-NFAT5 (H>K Mutant)	This paper
IMCD3 *Nfat5*$^{-/-}$ + GFP-NFAT5 (H>F Mutant)	This paper
IMCD3 *Nfat5*$^{-/-}$ + IBB-mVenus-mini-NFAT5 (H>K Mutant)	This paper
IMCD3 *Nfat5*$^{-/-}$ + IBB-mVenus-mini-NFAT5 (H>F Mutant)	This paper
IMCD3 *Nfat5*$^{-/-}$ + IBB-TurboID-1D4	This paper
IMCD3 *Nfat5*$^{-/-}$ + IBB-TurboID-mini-NFAT5-1D4	This paper
IMCD3 *Nfat5*$^{-/-}$ + IBB_CRY2PHR(1-498)-mCherry-ΔNLS/ΔNES/ΔAED-NFAT5	This paper
NIH/3T3- Flp In	Thermo Fisher Scientific (Cat# R76107)
NIH/3T3-Flp In *Nfat5*$^{-/-}$	This paper
NIH/3T3 *Nfat5*$^{-/-}$ + IBB-TurboID-1D4	This paper
NIH/3T3 *Nfat5*$^{-/-}$ + IBB-TurboID-mini-NFAT5-1D4	This paper
S. cerevisiae: Strain background: W303a (*MATa leu2-3,112 trp1-1 can1-100, ura3-1, ade2-1,* and *his3-11,15*)	Gift from Chris Burd (Yale University)
W303a *ura3*::8TonE-pCYC1-GFP-PEST	This paper
W303a *hog1*Δ::KanMX	This paper
W303a *ura3*::8TonE- pCYC1-GFP-PEST *leu2*::GAL-NLS-mRuby3-1D4	This paper

W303a *ura3*::8TonE- pCYC1-GFP-PEST *leu2*::GAL-NLS-mRuby3-RHD(DBD)-1D4	This paper
W303a *ura3*::-8TonE- pCYC1-GFP-PEST *leu2*::GAL-NLS-mRuby3-miniNFAT5-1D4	This paper
W303a *ura3*::8TonE- pCYC1-GFP-PEST *leu2*::GAL-NLS-mRuby3-miniNFAT5-1D4 (DBD mutant)	This paper
W303a *ura3*::8TonE- pCYC1-GFP-PEST *leu2*::GAL-NLS-mRuby3-miniNFAT5-1D4 (DIM mutant)	This paper
W303a *ura3*::8TonE(Mut)- pCYC1-GFP-PEST	This paper
W303a *ura3*::8TonE(Mut)- pCYC1-GFP-PEST *leu2*::GAL-NLS-mRuby3-1D4	This paper
W303a *ura3*::8TonE(Mut)- pCYC1-GFP-PEST *leu2*::GAL-NLS-mRuby3-RHD(DBD)-1D4	This paper
W303a *ura3*::-8TonE(Mut)- pCYC1-GFP-PEST *leu2*::GAL-NLS-mRuby3-miniNFAT5-1D4	This paper
W303a *ura3*::8TonE- pCYC1-GFP-PEST *leu2*::GAL-NLS-mRuby3-miniNFAT5-1D4 *hog1*Δ::KanMX	This paper
W303a *ura3*::8TonE- pCYC1-GFP-PEST *leu2*::GAL-NLS-mRuby3-miniNFAT5-1D4 *pbs2*Δ::HphMX	This paper
W303a *ura3*::8TonE- pCYC1-GFP-PEST *leu2*::GAL-NLS-mRuby3-miniNFAT5-1D4 *sho1*Δ::Hph *ssk2*Δ::Trp *ssk22*Δ::KanMX	This paper

Table S3: Oligonucleotides used in this study.

Oligonucleotides	Source
sgRNA sequences for CRISPR screen validation, see Data S2	This paper
sgRNA used to endogenously tag mouse Nfat5: GGGTCGAGCTGCGATGCCCT	This paper
qPCR Forward primer for mouse *Akr1b3*: CCTCAGGGAACGTGATACCT	This paper
qPCR Reverse primer for mouse *Akr1b3*: CAATCAGCTTCTCCTGAGTT	This paper
qPCR Forward primer for mouse *Slc5a3*: GGCAGCAGACATTGCCGTA	This paper
qPCR Reverse primer for mouse *Slc5a3*: AATCGCCACCCAGGTCATAGA	This paper
qPCR Forward primer for mouse *Slc6a12*: TCTTGGGCTTCATGTCTCAG	This paper
qPCR Reverse primer for mouse *Slc6a12*: GACCTGACTCAGCCACTTCA	This paper
qPCR Forward primer for mouse *Gapdh*: AGTGGCAAAGTGGAGATT	This paper
qPCR Reverse primer for mouse *Gapdh*: GTGGAGTCATACTGGAACA	This paper
qPCR Forward primer for reporter *GFP*: GTGGAGTCATACTGGAACA	This paper
qPCR Reverse primer for reporter *GFP*: GAACTTCAGGGTCAGCTTGC	This paper
qPCR Forward primer for yeast *STL1*: AGTGGCAAAGTGGAGATT	(*97*)
qPCR Reverse primer for yeast *STL1*: CATAGTTGAACTGTTTACC	(*97*)
qPCR Forward primer for yeast *ACT1*: GTGTGGGGAAGCGGGTAAGC	(*97*)
qPCR Reverse primer for yeast *ACT1*: GTGGCGGGTAAAGAAGAAAATGGA	(*97*)

Table S4: Recombinant DNA used in this study.

Recombinant DNA	Source	Identifier
Brie mouse CRISPR knockout pooled library	(*80*)	Addgene #73633
pMD2.G	Didier Trono Lab, EPFL, Switzerland	Addgene #12259
psPAX2	Didier Trono Lab, EPFL, Switzerland	Addgene #12260
pSpCas9(BB)-2A-Puro (PX459)	(*98*)	Addgene #48139
pSpCas9(BB)-2A-mCherry	(*27*)	N/A
pEF5/FRT/V5-DEST gateway vector	Thermo Fisher Scientific	Cat# V602020
pOG44 Flp-Recombinase expression vector	Thermo Fisher Scientific (Invitrogen)	Cat# V600520
pEF5/FRT/V5-DEST-8xTonE-GFP	This paper	N/A
pEF5/FRT/V5-DEST-FLAG-mVenus-*NFAT5*	This paper	N/A
pEF5/FRT/V5-DEST-GFP-*NFAT5*	This paper	N/A
pEF5/FRT/V5-DEST-3xHA-*NFAT5*	This paper	N/A
pEF5/FRT/V5-DEST-mCherry-*NFAT5*	This paper	N/A
pEF5/FRT/V5-DEST-GFP-*NFAT5*_CTD	This paper	N/A
pEF5/FRT/V5-DEST-GFP-*NFAT5*_PLD	This paper	N/A
pEF5/FRT/V5-DEST-IBB-mVenus-ΔNLS/ΔNES/ΔAED-*NFAT5*	This paper	N/A
pEF5/FRT/V5-DEST-IBB-mVenus-mini-NFAT5(DBD-MD1-AD2)	This paper	N/A
pAG306GAL-ccdB	Gift from Dan Jarosz (Susan Lindquist) (Stanford University)	Addgene #14139
pAG306-p*CYC1*-8xTonE-GFP	This paper	
pAG305GAL-ccdB	Gift from Dan Jarosz (Susan Lindquist) (Stanford University)	Addgene #14137
pAG305-pGAL-NLS-mRuby3	This paper	N/A
pAG305-pGAL-NLS-mRuby3-DBD	This paper	N/A
pAG305-pGAL-NLS-mRuby3-mini-NFAT5	This paper	N/A
pFA6a-kanMX6	Gift from Richard Chi (UNC Charlotte)	Addgene #39296
pFA6a-hphMX6	Gift from Richard Chi (UNC Charlotte)	N/A
pFA6a-TRP1	Gift from Richard Chi (UNC Charlotte)	Addgene #41595
pcDNA3.1_ionRD	(*41*)	Addgene #172931
pEF5/FRT/V5-DEST-GFP-*WNK1*	This paper	N/A
pEF5/FRT/V5-DEST-GFP-*NFAT5*(*H.sapiens*)_NTD-*NFAT5*(*D.melanogaster)*_CTD	This paper	N/A
pEF5/FRT/V5-DEST-GFP-*NFAT5*(*H.sapiens*)_NTD-*NFAT5*(*D.rerio)*_CTD	This paper	N/A

pEF5/FRT/V5-DEST-GFP-*NFAT5*(*H.sapiens*)_NTD-*NFAT5*(*X.laevis)*_CTD	This paper	N/A
pEF5/FRT/V5-DEST-GFP-*NFAT5*(*H.sapiens*)_NTD-*NFAT5*(*C.livia)*_CTD	This paper	N/A
pcDNA5-FRT/TO-FLAG-WNK1	Gift from Dario Alessi (University of Dundee)	N/A
pET-28a-GFP-*NFAT5*_CTD	This paper	N/A
pET-28a-GFP-*NFAT5*_PLD	This paper	N/A
pET-28a-GFP-*NFAT5*_CTD ΔPLD	This paper	N/A
pET-28a-GFP-*NFAT5*_CTD_H>F(AD2)	This paper	N/A
pET-28a-GFP-*NFAT5*_CTD_H>K(AD2)	This paper	N/A
pET-28a-*NFAT5*_CTD	This paper	N/A
pET-28a-*NFAT5*_PLD	This paper	N/A
pEF5/FRT/V5-DEST-GFP-*NFAT5*_Q>A(PLD)	This paper	N/A
pEF5/FRT/V5-DEST-GFP-*NFAT5*_S>A(PLD)	This paper	N/A
pEF5/FRT/V5-DEST-GFP-*NFAT5*_H>K(AD2)	This paper	N/A
pEF5/FRT/V5-DEST-GFP-*NFAT5*_H>F(AD2)	This paper	N/A
pEF5/FRT/V5-DEST-IBB-mVenus-mini-NFAT5(DBD-MD1-AD2)_H>K(AD2)	This paper	N/A
pEF5/FRT/V5-DEST-IBB-mVenus-mini-NFAT5(DBD-MD1-AD2)_H>F(AD2)	This paper	N/A
pUC19-NFAT5/mNeonGreen	This paper	N/A
pMJ915	(*99*)	Addgene #69090
pEF5/FRT/V5-DEST-sfGFP-*NFAT1*(*H.sapiens*)	This paper	N/A
pEF5/FRT/V5-DEST-sfGFP-*NFAT2*(*H.sapiens*)	This paper	N/A
pEF5/FRT/V5-DEST-sfGFP-*NFAT3*(*H.sapiens*)	This paper	N/A
pEF5/FRT/V5-DEST-sfGFP-*NFAT4*(*H.sapiens*)	This paper	N/A
pMIG-hNFATc2	(*100*)	Addgene #74050
pREP-NFAT2	Anjana Rao Lab, La Jolla Institute for Immunology	Addgene #11788
pEGFP-C1 NFAT3	(*101*)	Addgene #10961
MAC_N_NFAC3	(*102*)	Addgene #167781
pHR-MCS-MED1long-IDR-YFP-NLS	(*49*)	Addgene #145276
pRK5-mEGFP-HMGB1-WT-IDR	(*56*)	Addgene #194549
pHR-HOXD13-IDR-YFP-NLS	(*49*)	Addgene #145277
pmCherry-TEAD4-IDR	(*103*)	Addgene #166451
pcDNA5-Flag-BRD4-WT	(*104*)	Addgene #90331
pHR-HNRNPA1C-mCh-Cry2WT	(*70*)	Addgene #101226
pHR-DDX4N-mCh-Cry2WT	(*70*)	Addgene #101225

p5068 pGEX-6P-1 Brd4 full-length	(*105*)	Addgene #14447
GAL4UAS-Luciferase reporter	(*106*)	Addgene #64125
pRL-TK-Renilla luciferase	Promega	Cat #E2241
pGAL4-DBD-MCS-long_	(*49*)	Addgene #145246
pGAL4-DBD-NFAT5-CTD	This paper	N/A
pGAL4-DBD-NFAT5-PLD	This paper	N/A
pGAL4-DBD-NFAT5-NTD	This paper	N/A
pGAL4-DBD-*NFAT5*-PLD_Fragment1	This paper	N/A
pGAL4-DBD-*NFAT5*-PLD_Fragment2	This paper	N/A
pGAL4-DBD-*NFAT5*-PLD_Fragment3	This paper	N/A
pGAL4-DBD-VP16 AD	This paper	N/A
pGAL4-DBD-*NFAT5*-N-termFragment	This paper	N/A
pEF5/FRT/V5-DEST-sfGFP-BRD4-IDR	This paper	N/A
pEF5/FRT/V5-DEST-sfGFP-HNRNPA1-IDR	This paper	N/A
pEF5/FRT/V5-DEST-sfGFP-DDX4-IDR	This paper	N/A
pEF5/FRT/V5-DEST-sfGFP-Brd4-IDR	This paper	N/A
pEF5/FRT/V5-DEST-sfGFP-Fus-IDR	This paper	N/A
pEF5/FRT/V5-DEST-sfGFP-TDP43-IDR	This paper	N/A
pNLS-TetR-GFP	(*107*)	Addgene #103838
pEGFP-TetR-NLS-VP16	(*107*)	Addgene #103834
pEGFP-TetR-NLS-*NFAT5*_CTD	This paper	N/A
pEGFP-TetR-NLS-*NFAT5*_PLD	This paper	N/A
pEGFP-TetR-NLS-*NFAT5*_CTDΔPLD	This paper	N/A
pEGFP-TetR-NLS-*NFAT5*_PLD_Fragment1	This paper	N/A
pEGFP-TetR-NLS-*NFAT5*_PLD_Fragment2	This paper	N/A
pEGFP-TetR-NLS-*NFAT5*_PLD_Fragment3	This paper	N/A
pEGFP-TetR-NLS-*NFAT5*_PLD_N-termFragment	This paper	N/A
CRY2PHR(1-498)_mcherry	Gift from Bianxiao Cui Lab (Stanford University)	N/A
CRY2PHR(1-498)_mcherry_*NFAT5*	This paper	N/A
IBB_CRY2PHR(1-498)_mcherry_ΔNLS/ΔNES/ΔAED-*NFAT5*	This paper	N/A
pcDNA3.1-1xHA_*NFAT5*_PLD_Fragment1	This paper	N/A
pcDNA3.1-1xHA_*NFAT5*_PLD_Fragment2	This paper	N/A
pcDNA3.1-1xHA_*NFAT5*_PLD_Fragment3	This paper	N/A
pcDNA3.1-1xHA_*NFAT5*_PLD_N-termFragment	This paper	N/A
pEF5/FRT/V5-DEST-IBB-TurboID-1D4	This paper	N/A
pEF5/FRT/V5-DEST-IBB-TurboID-mini-NFAT5-1D4	This paper	N/A

Table S5: Software and algorithms used in this study.

Software and algorithms	Source	Identifier
CRISPR Guide RNA Design Tool	Benchling	https://www.benchling.com/crispr
Synthego ICE analysis	Synthego Corporation	https://www.synthego.com/products/bioinformatics/crispr-analysis
SnapGene software	GSL Biotech	http://snapgene.com
Leica Application Suite X	Leica Microsystems	https://www.leica-microsystems.com/products/microscope-software/p/leica-las-x-ls/
Metapredict	(*93*)	https://metapredict.net/
PLAAC: Prion-Like Amino Acid Composition	(*94*)	http://plaac.wi.mit.edu/
AlphaFold	*(96)*	https://alphafold.ebi.ac.uk/
Adobe Illustrator CS6	Adobe Systems	https://www.adobe.com/products/illustrator.html
Biorender	Biorender	https://www.biorender.com/
GraphPad Prism	GraphPad Software	https://www.graphpad.com/
ImageJ	(*108*)	https://imagej.nih.gov/ij/
CellProfiler	(*109*)	https://cellprofiler.org/
MaxQuant v1.6.17.0	*(90)*	https://www.maxquant.org/maxquant/
GSEA	*(92)*	https://www.gsea-msigdb.org/gsea/index.jsp

Data S1. Results from CRISPR/Cas9-based and insertional mutagenesis-based loss of function screens.

Data S2. Single guide RNA sequences for CRISPR screen validation and clonal knockout cell line generation

Data S3. Results from proximity labeling of TurboID-mini-NFAT5 in IMCD3 or NIH/3T3 cells.

Movie 1. NFAT5 condensate formation in HEK293T cells, related to Figure 4. Live cell imaging of transiently expressed full-length GFP-NFAT5.

Movie 2. NFAT5 nuclear condensate formation in IMCD3 cells, related to Figure 4. Live cell imaging of stably expressed full-length GFP-NFAT5 at low levels from a single genomic locus in *Nfat5*$^{-/-}$ IMCD3 cells.

Movie 3. NFAT5 nuclear condensate formation in IMCD3 cells, related to Figure 4. Live cell imaging of endogenously mNeonGreen-tagged NFAT5.

Movie 4. Self-association of Cry2 alone upon exposure to blue light, related to Figure S10. Live cell imaging of transiently expressed Cry2WT–mCherry in HEK293T cells**.**

Movie 5. Self-association of Cry2-NFAT5 upon exposure to blue light, related to Figure S10. Live cell imaging of transiently expressed Cry2WT–mCherry–Full-length NFAT5 in HEK293T cells**.**

Movie 6. Self-association of Cry2-nuclear NFAT5 upon exposure to blue light, related to Figure S10. Live cell imaging of transiently expressed IBB–Cry2WT–mCherry–ΔNLS/ΔNES/ΔAED NFAT5 in HEK293T cells**.**

REFERENCES AND NOTES

1. P. H. Yancey, M. E. Clark, S. C. Hand, R. D. Bowlus, G. N. Somero, Living with water stress: Evolution of osmolyte systems. *Science* **217**, 1214–1222 (1982).

2. M. B. Burg, J. D. Ferraris, N. I. Dmitrieva, Cellular response to hyperosmotic stresses. *Physiol. Rev.* **87**, 1441–1474 (2007).

3. R. J. Johnson, L. G. Sánchez-Lozada, L. S. Newman, M. A. Lanaspa, H. F. Diaz, J. Lemery, B. Rodriguez-Iturbe, D. R. Tolan, J. Butler-Dawson, Y. Sato, G. Garcia, A. A. Hernando, C. A. Roncal-Jimenez, Climate change and the kidney. *Ann. Nutr. Metab.* **74** (Suppl. 3), 38–44 (2019).

4. A. Garcia-Perez, M. B. Burg, Renal medullary organic osmolytes. *Physiol. Rev.* **71**, 1081–1115 (1991).

5. Time to sound the alarm about the hidden epidemic of kidney disease, *Nature Publishing Group UK* (2024). https://doi.org/10.1038/d41586-024-00961-5.

6. T. J. Jentsch, VRACs and other ion channels and transporters in the regulation of cell volume and beyond. *Nat. Rev. Mol. Cell Biol.* **17**, 293–307 (2016).

7. E. Delpire, K. B. Gagnon, "Chapter one - Water homeostasis and cell volume maintenance and regulation" in *Current Topics in Membranes*, I. Levitane, E. Delpire, H. Rasgado-Flores, Eds. (Academic Press, 2018), pp. 3–52, vol. 81.

8. E. K. Hoffmann, I. H. Lambert, S. F. Pedersen, Physiology of cell volume regulation in vertebrates. *Physiol. Rev.* **89**, 193–277 (2009).

9. C. R. Boyd-Shiwarski, D. J. Shiwarski, S. E. Griffiths, R. T. Beacham, L. Norrell, D. E. Morrison, J. Wang, J. Mann, W. Tennant, E. N. Anderson, J. Franks, M. Calderon, K. A. Connolly, M. U. Cheema, C. J. Weaver, L. J. Nkashama, C. C. Weckerly, K. E. Querry, U. B. Pandey, C. J. Donnelly, D. Sun, A. R. Rodan, A. R. Subramanya, WNK kinases sense molecular crowding and rescue cell volume via phase separation. *Cell* **185**, 4488–4506.e20 (2022).

10. D. Kültz, D. Chakravarty, Hyperosmolality in the form of elevated NaCl but not urea causes DNA damage in murine kidney cells. *Proc. Natl. Acad. Sci. U.S.A.* **98**, 1999–2004 (2001).

11. P. Nunes, I. Roth, P. Meda, E. Féraille, D. Brown, U. Hasler, Ionic imbalance, in addition to molecular crowding, abates cytoskeletal dynamics and vesicle motility during hypertonic stress. *Proc. Natl. Acad. Sci. U.S.A.* **112**, E3104–E3113 (2015).

12. G. N. Somero, Protons, osmolytes, and fitness of internal milieu for protein function. *Am. J. Physiol.* **251**, R197–R213 (1986).

13. M. B. Burg, J. D. Ferraris, Intracellular organic osmolytes: Function and regulation. *J. Biol. Chem.* **283**, 7309–7313 (2008).

14. J. H. Cha, S. K. Woo, K. H. Han, Y. H. Kim, J. S. Handler, J. Kim, H. M. Kwon, Hydration status affects nuclear distribution of transcription factor tonicity responsive enhancer binding protein in rat kidney. *J. Am. Soc. Nephrol.* **12**, 2221–2230 (2001).

15. C. López-Rodríguez, C. L. Antos, J. M. Shelton, J. A. Richardson, F. Lin, T. I. Novobrantseva, R. T. Bronson, P. Igarashi, A. Rao, E. N. Olson, Loss of NFAT5 results in renal atrophy and lack of tonicity-responsive gene expression. *Proc. Natl. Acad. Sci. U.S.A.* **101**, 2392–2397 (2004).

16. J. L. Brewster, M. C. Gustin, Hog1: 20 years of discovery and impact. *Sci. Signal.* **7**, re7 (2014).

17. J. L. Brewster, T. de Valoir, N. D. Dwyer, E. Winter, M. C. Gustin, An osmosensing signal transduction pathway in yeast. *Science* **259**, 1760–1763 (1993).

18. H. Miyakawa, S. K. Woo, S. C. Dahl, J. S. Handler, H. M. Kwon, Tonicity-responsive enhancer binding protein, a rel-like protein that stimulates transcription in response to hypertonicity. *Proc. Natl. Acad. Sci. U.S.A.* **96**, 2538–2542 (1999).

19. C. Lopez-Rodríguez, J. Aramburu, A. S. Rakeman, A. Rao, NFAT5, a constitutively nuclear NFAT protein that does not cooperate with Fos and Jun. *Proc. Natl. Acad. Sci. U.S.A.* **96**, 7214–7219 (1999).

20. M. Gamboa, N. Kitamura, K. Miura, S. Noda, O. Kaminuma, Evolutionary mechanisms underlying the diversification of nuclear factor of activated T cells across vertebrates. *Sci. Rep.* **13**, 6468 (2023).

21. M. B. Burg, E. D. Kwon, D. Kültz, Regulation of gene expression by hypertonicity. *Annu. Rev. Physiol.* **59**, 437–455 (1997).

22. Y. Izumi, W. Yang, J. Zhu, M. B. Burg, J. D. Ferraris, RNA-seq analysis of high NaCl-induced gene expression. *Physiol. Genomics* **47**, 500–513 (2015).

23. S. D. Lee, S. Y. Choi, S. W. Lim, S. T. Lamitina, S. N. Ho, W. Y. Go, H. M. Kwon, TonEBP stimulates multiple cellular pathways for adaptation to hypertonic stress: Organic osmolyte-dependent and -independent pathways. *Am. J. Physiol. Renal Physiol.* **300**, F707–F715 (2011).

24. M. I. Rauchman, S. K. Nigam, E. Delpire, S. R. Gullans, An osmotically tolerant inner medullary collecting duct cell line from an SV40 transgenic mouse. *Am. J. Physiol.* **265**, F416–24 (1993).

25. J. D. Ferraris, C. K. Williams, K. Y. Jung, J. J. Bedford, M. B. Burg, A. García-Pérez, ORE, a eukaryotic minimal essential osmotic response element. The aldose reductase gene in hyperosmotic stress. *J. Biol. Chem.* **271**, 18318–18321 (1996).

26. K. T. Farabaugh, D. Krokowski, B.-J. Guan, Z. Gao, X.-H. Gao, J. Wu, R. Jobava, G. Ray, T. J. de Jesus, M. G. Bianchi, E. Chukwurah, O. Bussolati, M. Kilberg, D. A. Buchner, G. C. Sen, C. Cotton, C. McDonald, M. Longworth, P. Ramakrishnan, M. Hatzoglou, PACT-mediated PKR activation acts as a hyperosmotic stress intensity sensor weakening osmoadaptation and enhancing inflammation. *eLife* **9**, e52241 (2020).

27. G. V. Pusapati, J. H. Kong, B. B. Patel, A. Krishnan, A. Sagner, M. Kinnebrew, J. Briscoe, L. Aravind, R. Rohatgi, CRISPR Screens Uncover Genes that Regulate Target Cell Sensitivity to the Morphogen Sonic Hedgehog. *Dev. Cell* **44**, 271 (2018).

28. A. M. Lebensohn, R. Dubey, L. R. Neitzel, O. Tacchelly-Benites, E. Yang, C. D. Marceau, E. M. Davis, B. B. Patel, Z. Bahrami-Nejad, K. J. Travaglini, Y. Ahmed, E. Lee, J. E. Carette, R.

Rohatgi, Comparative genetic screens in human cells reveal new regulatory mechanisms in WNT signaling. *eLife* **5**, e21459 (2016).

29. E. H. Y. Tong, J.-J. Guo, A.-L. Huang, H. Liu, C.-D. Hu, S. S. M. Chung, B. C. B. Ko, Regulation of nucleocytoplasmic trafficking of transcription factor OREBP/TonEBP/NFAT5. *J. Biol. Chem.* **281**, 23870–23879 (2006).

30. S. Do Lee, E. Colla, M. R. Sheen, K. Y. Na, H. M. Kwon, Multiple domains of TonEBP cooperate to stimulate transcription in response to hypertonicity. *J. Biol. Chem.* **278**, 47571–47577 (2003).

31. C. López-Rodríguez, J. Aramburu, L. Jin, A. S. Rakeman, M. Michino, A. Rao, Bridging the NFAT and NF-κB families: NFAT5 dimerization regulates cytokine gene transcription in response to osmotic stress. *Immunity* **15**, 47–58 (2001).

32. S. K. Woo, S. D. Lee, K. Y. Na, W. K. Park, H. M. Kwon, TonEBP/NFAT5 stimulates transcription of HSP70 in response to hypertonicity. *Mol. Cell. Biol.* **22**, 5753–5760 (2002).

33. T. Maeda, M. Takekawa, H. Saito, Activation of yeast PBS2 MAPKK by MAPKKKs or by binding of an SH3-containing osmosensor. *Science* **269**, 554–558 (1995).

34. T. Rødgaard, K. Schou, M. B. Friis, E. K. Hoffmann, Does the intracellular ionic concentration or the cell water content (cell volume) determine the activity of TonEBP in NIH3T3 cells? *Am. J. Physiol. Cell Physiol.* **295**, C1528–C1534 (2008).

35. W. Neuhofer, S. K. Woo, K. Y. Na, R. Grunbein, W. K. Park, O. Nahm, F.-X. Beck, H. M. Kwon, Regulation of TonEBP transcriptional activator in MDCK cells following changes in ambient tonicity. *Am. J. Physiol. Cell Physiol.* **283**, C1604–11 (2002).

36. M. B. Burg, Macromolecular crowding as a cell volume sensor. *Cell. Physiol. Biochem.* **10**, 251–256 (2000).

37. W. Neuhofer, H. Bartels, M.-L. Fraek, F.-X. Beck, Relationship between intracellular ionic strength and expression of tonicity-responsive genes in rat papillary collecting duct cells. *J. Physiol.* **543**, 147–153 (2002).

38. S. N. Ho, The role of NFAT5/TonEBP in establishing an optimal intracellular environment. *Arch. Biochem. Biophys.* **413**, 151–157 (2003).

39. M. H. Jacobs, D. R. Stewart, The distribution of penetrating ammonium salts between cells and their surroundings. *J. Cell. Comp. Physiol.* **7**, 351–365 (1936).

40. M. S. Hamaguchi, K. Watanabe, Y. Hamaguchi, Regulation of intracellular pH in sea urchin eggs by medium containing both weak acid and base. *Cell Struct. Funct.* **22**, 387–398 (1997).

41. B. Liu, B. Poolman, A. J. Boersma, Ionic Strength Sensing in Living Cells. *ACS Chem. Biol.* **12**, 2510–2514 (2017).

42. E. Krump, K. Nikitas, S. Grinstein, Induction of tyrosine phosphorylation and Na^+/H^+ exchanger activation during shrinkage of human neutrophils. *J. Biol. Chem.* **272**, 17303–17311 (1997).

43. R. G. Roeder, 50+ years of eukaryotic transcription: An expanding universe of factors and mechanisms. *Nat. Struct. Mol. Biol.* **26**, 783–791 (2019).

44. I. Kwon, M. Kato, S. Xiang, L. Wu, P. Theodoropoulos, H. Mirzaei, T. Han, S. Xie, J. L. Corden, S. L. McKnight, Phosphorylation-regulated binding of RNA polymerase II to fibrous polymers of low-complexity domains. *Cell* **155**, 1049–1060 (2013).

45. S. Chong, C. Dugast-Darzacq, Z. Liu, P. Dong, G. M. Dailey, C. Cattoglio, A. Heckert, S. Banala, L. Lavis, X. Darzacq, R. Tjian, Imaging dynamic and selective low-complexity domain interactions that control gene transcription. *Science* **361**, eaar2555 (2018).

46. A. Boija, I. A. Klein, B. R. Sabari, A. Dall'Agnese, E. L. Coffey, A. V. Zamudio, C. H. Li, K. Shrinivas, J. C. Manteiga, N. M. Hannett, B. J. Abraham, L. K. Afeyan, Y. E. Guo, J. K. Rimel, C. B. Fant, J. Schuijers, T. I. Lee, D. J. Taatjes, R. A. Young, Transcription factors activate

genes through the phase-separation capacity of their activation domains. *Cell* **175**, 1842–1855.e16 (2018).

47. B. R. Sabari, Alessandra Dall'Agnese, A. Boija, I. A. Klein, E. L. Coffey, K. Shrinivas, B. J. Abraham, N. M. Hannett, A. V. Zamudio, J. C. Manteiga, C. H. Li, Y. E. Guo, D. S. Day, J. Schuijers, E. Vasile, S. Malik, D. Hnisz, T. I. Lee, I. I. Cisse, R. G. Roeder, P. A. Sharp, A. K. Chakraborty, R. A. Young, Coactivator condensation at super-enhancers links phase separation and gene control. *Science* **361**, eaar3958 (2018).

48. W.-K. Cho, J.-H. Spille, M. Hecht, C. Lee, C. Li, V. Grube, I. I. Cisse, Mediator and RNA polymerase II clusters associate in transcription-dependent condensates. *Science* **361**, 412–415 (2018).

49. S. Basu, S. D. Mackowiak, H. Niskanen, D. Knezevic, V. Asimi, S. Grosswendt, H. Geertsema, S. Ali, I. Jerković, H. Ewers, S. Mundlos, A. Meissner, D. M. Ibrahim, D. Hnisz, Unblending of transcriptional condensates in human repeat expansion disease. *Cell* **181**, 1062–1079.e30 (2020).

50. M.-T. Wei, Y.-C. Chang, S. F. Shimobayashi, Y. Shin, A. R. Strom, C. P. Brangwynne, Nucleated transcriptional condensates amplify gene expression. *Nat. Cell Biol.* **22**, 1187–1196 (2020).

51. M. Kato, S. L. McKnight, A solid-state conceptualization of information transfer from gene to message to protein. *Annu. Rev. Biochem.* **87**, 351–390 (2018).

52. J. J. Ferrie, J. P. Karr, R. Tjian, X. Darzacq, "Structure"-function relationships in eukaryotic transcription factors: The role of intrinsically disordered regions in gene regulation. *Mol. Cell* **82**, 3970–3984 (2022).

53. J. Trojanowski, L. Frank, A. Rademacher, N. Mücke, P. Grigaitis, K. Rippe, Transcription activation is enhanced by multivalent interactions independent of phase separation. *Mol. Cell* **82**, 1878–1893.e10 (2022).

54. Y. Shin, C. P. Brangwynne, Liquid phase condensation in cell physiology and disease. *Science* **357**, eaaf4382 (2017).

55. S. F. Banani, H. O. Lee, A. A. Hyman, M. K. Rosen, Biomolecular condensates: Organizers of cellular biochemistry. *Nat. Rev. Mol. Cell Biol.* **18**, 285–298 (2017).

56. M. A. Mensah, H. Niskanen, A. P. Magalhaes, S. Basu, M. Kircher, H. L. Sczakiel, A. M. V. Reiter, J. Elsner, P. Meinecke, S. Biskup, B. H. Y. Chung, G. Dombrowsky, C. Eckmann-Scholz, M. P. Hitz, A. Hoischen, P.-M. Holterhus, W. Hülsemann, K. Kahrizi, V. M. Kalscheuer, A. Kan, M. Krumbiegel, I. Kurth, J. Leubner, A. C. Longardt, J. D. Moritz, H. Najmabadi, K. Skipalova, L. Snijders Blok, A. Tzschach, E. Wiedersberg, M. Zenker, C. Garcia-Cabau, R. Buschow, X. Salvatella, M. L. Kraushar, S. Mundlos, A. Caliebe, M. Spielmann, D. Horn, D. Hnisz, Aberrant phase separation and nucleolar dysfunction in rare genetic diseases. *Nature* **614**, 564–571 (2023).

57. Z. M. March, O. D. King, J. Shorter, Prion-like domains as epigenetic regulators, scaffolds for subcellular organization, and drivers of neurodegenerative disease. *Brain Res.* **1647**, 9–18 (2016).

58. J.-H. Jung, A. D. Barbosa, S. Hutin, J. R. Kumita, M. Gao, D. Derwort, C. S. Silva, X. Lai, E. Pierre, F. Geng, S.-B. Kim, S. Baek, C. Zubieta, K. E. Jaeger, P. A. Wigge, A prion-like domain in ELF3 functions as a thermosensor in Arabidopsis. *Nature* **585**, 256–260 (2020).

59. M. Zhang, C. Zhu, Y. Duan, T. Liu, H. Liu, C. Su, Y. Lu, The intrinsically disordered region from PP2C phosphatases functions as a conserved CO_2 sensor. *Nat. Cell Biol.* **24**, 1029–1037 (2022).

60. T. M. Franzmann, M. Jahnel, A. Pozniakovsky, J. Mahamid, A. S. Holehouse, E. Nüske, D. Richter, W. Baumeister, S. W. Grill, R. V. Pappu, A. A. Hyman, S. Alberti, Phase separation of a yeast prion protein promotes cellular fitness. *Science* **359**, eaao5654 (2018).

61. D. Moses, G. M. Ginell, A. S. Holehouse, S. Sukenik, Intrinsically disordered regions are poised to act as sensors of cellular chemistry. *Trends Biochem. Sci.* **48**, 1019–1034 (2023).

62. J. D. Ferraris, C. K. Williams, P. Persaud, Z. Zhang, Y. Chen, M. B. Burg, Activity of the TonEBP/OREBP transactivation domain varies directly with extracellular NaCl concentration. *Proc. Natl. Acad. Sci. U.S.A.* **99**, 739–744 (2002).

63. S. L. McKnight, Protein domains of low sequence complexity-dark matter of the proteome. *Genes Dev.* **38**, 205–212 (2024).

64. T. Mittag, R. V. Pappu, A conceptual framework for understanding phase separation and addressing open questions and challenges. *Mol. Cell* **82**, 2201–2214 (2022).

65. K. Ribbeck, D. Görlich, The permeability barrier of nuclear pore complexes appears to operate via hydrophobic exclusion. *EMBO J.* **21**, 2664–2671 (2002).

66. J. Gu, X. Zhou, L. Sutherland, M. Kato, K. Jaczynska, J. Rizo, S. L. McKnight, Oxidative regulation of TDP-43 self-association by a β-to-α conformational switch. *Proc. Natl. Acad. Sci. U.S.A.* **120**, e2311416120 (2023).

67. T. M. Perdikari, A. C. Murthy, N. L. Fawzi, Molecular insights into the effect of alkanediols on FUS liquid-liquid phase separation. bioRxiv 490812 [Preprint] (2022). https://doi.org/10.1101/2022.05.05.490812.

68. M. F. Perutz, T. Johnson, M. Suzuki, J. T. Finch, Glutamine repeats as polar zippers: Their possible role in inherited neurodegenerative diseases. *Proc. Natl. Acad. Sci. U.S.A.* **91**, 5355–5358 (1994).

69. J. B. Matthew, G. I. Hanania, F. R. Gurd, Electrostatic effects in hemoglobin: Bohr effect and ionic strength dependence of individual groups. *Biochemistry* **18**, 1928–1936 (1979).

70. Y. Shin, J. Berry, N. Pannucci, M. P. Haataja, J. E. Toettcher, C. P. Brangwynne, Spatiotemporal control of intracellular phase transitions using light-activated optoDroplets, *Cell* **168**, 159–171.e14 (2017).

71. K. F. Cho, T. C. Branon, N. D. Udeshi, S. A. Myers, S. A. Carr, A. Y. Ting, Proximity labeling in mammalian cells with TurboID and split-TurboID. *Nat. Protoc.* **15**, 3971–3999 (2020).

72. P. Filippakopoulos, J. Qi, S. Picaud, Y. Shen, W. B. Smith, O. Fedorov, E. M. Morse, T. Keates, T. T. Hickman, I. Felletar, M. Philpott, S. Munro, M. R. McKeown, Y. Wang, A. L. Christie, N. West, M. J. Cameron, B. Schwartz, T. D. Heightman, N. La Thangue, C. A. French, O. Wiest, A. L. Kung, S. Knapp, J. E. Bradner, Selective inhibition of BET bromodomains. *Nature* **468**, 1067–1073 (2010).

73. S. M. Janicki, T. Tsukamoto, S. E. Salghetti, W. P. Tansey, R. Sachidanandam, K. V. Prasanth, T. Ried, Y. Shav-Tal, E. Bertrand, R. H. Singer, D. L. Spector, From silencing to gene expression: Real-time analysis in single cells. *Cell* **116**, 683–698 (2004).

74. A. V. Zamudio, A. Dall'Agnese, J. E. Henninger, J. C. Manteiga, L. K. Afeyan, N. M. Hannett, E. L. Coffey, C. H. Li, O. Oksuz, B. R. Sabari, A. Boija, I. A. Klein, S. W. Hawken, J.-H. Spille, T.-M. Decker, I. I. Cisse, B. J. Abraham, T. I. Lee, D. J. Taatjes, J. Schuijers, R. A. Young, Mediator condensates localize signaling factors to key cell identity genes. *Mol. Cell* **76**, 753–766.e6 (2019).

75. S. Fukuchi, K. Yoshimune, M. Wakayama, M. Moriguchi, K. Nishikawa, Unique amino acid composition of proteins in halophilic bacteria. *J. Mol. Biol.* **327**, 347–357 (2003).

76. C. Y. Cheung, T.-T. Huang, N. Chow, S. Zhang, Y. Zhao, M. P. Chau, W. C. Chan, C. C. L. Wong, D. Boassa, S. Phan, M. H. Ellisman, J. R. Yates, S. Xu, Z. Yu, Y. Zhang, R. Zhang, L. L. Ng, B. C. B. Ko, Unconventional tonicity-regulated nuclear trafficking of NFAT5 mediated by KPNB1, XPOT and RUVBL2. *J. Cell Sci.* **135**, jcs259280 (2022).

77. T. Olbrich, M. Vega-Sendino, M. Murga, G. de Carcer, M. Malumbres, S. Ortega, S. Ruiz, O. Fernandez-Capetillo, A chemical screen identifies compounds capable of selecting for haploidy in mammalian cells. *Cell Rep.* **28**, 597–604.e4 (2019).

78. S. Mukhopadhyay, X. Wen, B. Chih, C. D. Nelson, W. S. Lane, S. J. Scales, P. K. Jackson, TULP3 bridges the IFT-A complex and membrane phosphoinositides to promote trafficking of G protein-coupled receptors into primary cilia. *Genes Dev.* **24**, 2180–2193 (2010).

79. J. Joung, S. Konermann, J. S. Gootenberg, O. O. Abudayyeh, R. J. Platt, M. D. Brigham, N. E. Sanjana, F. Zhang, Author correction: Genome-scale CRISPR-Cas9 knockout and transcriptional activation screening. *Nat. Protoc.* **14**, 2259 (2019).

80. J. G. Doench, N. Fusi, M. Sullender, M. Hegde, E. W. Vaimberg, K. F. Donovan, I. Smith, Z. Tothova, C. Wilen, R. Orchard, H. W. Virgin, J. Listgarten, D. E. Root, Optimized sgRNA design to maximize activity and minimize off-target effects of CRISPR-Cas9. *Nat. Biotechnol.* **34**, 184–191 (2016).

81. W. Li, H. Xu, T. Xiao, L. Cong, M. I. Love, F. Zhang, R. A. Irizarry, J. S. Liu, M. Brown, X. S. Liu, MAGeCK enables robust identification of essential genes from genome-scale CRISPR/Cas9 knockout screens. *Genome Biol.* **15**, 554 (2014).

82. J. Diessl, A. Nandy, C. Schug, L. Habernig, S. Büttner, Stable and destabilized GFP reporters to monitor calcineurin activity in *Saccharomyces cerevisiae*. *Microb. Cell Fact.* **7**, 106–114 (2020).

83. D. Görlich, P. Henklein, R. A. Laskey, E. Hartmann, A 41 amino acid motif in importin-alpha confers binding to importin-beta and hence transit into the nucleus. *EMBO J.* **15**, 1810–1817 (1996).

84. M. S. Longtine, A. McKenzie 3rd, D. J. Demarini, N. G. Shah, A. Wach, A. Brachat, P. Philippsen, J. R. Pringle, Additional modules for versatile and economical PCR-based gene deletion and modification in Saccharomyces cerevisiae. *Yeast* **14**, 953–961 (1998).

85. T. Nakamura, Y. Liu, D. Hirata, H. Namba, S. Harada, T. Hirokawa, T. Miyakawa, Protein phosphatase type 2B (calcineurin)-mediated, FK506-sensitive regulation of intracellular ions in yeast is an important determinant for adaptation to high salt stress conditions. *EMBO J.* **12**, 4063–4071 (1993).

86. N. J. Raat, P. De Smet, W. van Driessche, R. J. Bindels, C. H. Van Os, Measuring volume perturbation of proximal tubular cells in primary culture with three different techniques. *Am. J. Physiol.* **271**, C235–C241 (1996).

87. S. Sukenik, P. Ren, M. Gruebele, Weak protein-protein interactions in live cells are quantified by cell-volume modulation. *Proc. Natl. Acad. Sci. U.S.A.* **114**, 6776–6781 (2017).

88. S. Sukenik, M. Salam, Y. Wang, M. Gruebele, In-cell titration of small solutes controls protein stability and aggregation. *J. Am. Chem. Soc.* **140**, 10497–10503 (2018).

89. E. Lingeman, C. Jeans, J. E. Corn, Production of purified CasRNPs for efficacious genome editing. *Curr. Protoc. Mol. Biol.* **120**, 31.10.1–31.10.19 (2017).

90. J. Cox, M. Mann, MaxQuant enables high peptide identification rates, individualized p.p.b.-range mass accuracies and proteome-wide protein quantification. *Nat. Biotechnol.* **26**, 1367–1372 (2008).

91. Y. Perez-Riverol, J. Bai, C. Bandla, D. García-Seisdedos, S. Hewapathirana, S. Kamatchinathan, D. J. Kundu, A. Prakash, A. Frericks-Zipper, M. Eisenacher, M. Walzer, S. Wang, A. Brazma, J. A. Vizcaíno, The PRIDE database resources in 2022: A hub for mass spectrometry-based proteomics evidences. *Nucleic Acids Res.* **50**, D543–D552 (2022).

92. A. Subramanian, P. Tamayo, V. K. Mootha, S. Mukherjee, B. L. Ebert, M. A. Gillette, A. Paulovich, S. L. Pomeroy, T. R. Golub, E. S. Lander, J. P. Mesirov, Gene set enrichment analysis: A knowledge-based approach for interpreting genome-wide expression profiles. *Proc. Natl. Acad. Sci. U.S.A.* **102**, 15545–15550 (2005).

93. R. J. Emenecker, D. Griffith, A. S. Holehouse, Metapredict: A fast, accurate, and easy-to-use predictor of consensus disorder and structure. *Biophys. J.* **120**, 4312–4319 (2021).

94. A. K. Lancaster, A. Nutter-Upham, S. Lindquist, O. D. King, PLAAC: A web and command-line application to identify proteins with prion-like amino acid composition. *Bioinformatics* **30**, 2501–2502 (2014).

95. T. Kino, H. Takatori, I. Manoli, Y. Wang, A. Tiulpakov, M. R. Blackman, Y. A. Su, G. P. Chrousos, A. H. DeCherney, J. H. Segars, Brx mediates the response of lymphocytes to osmotic stress through the activation of NFAT5. *Sci. Signal.* **2**, ra5 (2009).

96. J. Jumper, R. Evans, A. Pritzel, T. Green, M. Figurnov, O. Ronneberger, K. Tunyasuvunakool, R. Bates, A. Žídek, A. Potapenko, A. Bridgland, C. Meyer, S. A. A. Kohl, A. J. Ballard, A. Cowie, B. Romera-Paredes, S. Nikolov, R. Jain, J. Adler, T. Back, S. Petersen, D. Reiman, E. Clancy, M. Zielinski, M. Steinegger, M. Pacholska, T. Berghammer, S. Bodenstein, D. Silver, O. Vinyals, A. W. Senior, K. Kavukcuoglu, P. Kohli, D. Hassabis, Highly accurate protein structure prediction with AlphaFold. *Nature* **596**, 583–589 (2021).

97. C. Bai, M. Tesker, D. Engelberg, The yeast Hot1 transcription factor is critical for activating a single target gene, STL1. *Mol. Biol. Cell* **26**, 2357–2374 (2015).

98. F. A. Ran, P. D. Hsu, J. Wright, V. Agarwala, D. A. Scott, F. Zhang, Genome engineering using the CRISPR-Cas9 system. *Nat. Protoc.* **8**, 2281–2308 (2013).

99. S. Lin, B. T. Staahl, R. K. Alla, J. A. Doudna, Enhanced homology-directed human genome engineering by controlled timing of CRISPR/Cas9 delivery. *eLife* **3**, e04766 (2014).

100. C. H. Gabriel, F. Gross, M. Karl, H. Stephanowitz, A. F. Hennig, M. Weber, S. Gryzik, I. Bachmann, K. Hecklau, J. Wienands, J. Schuchhardt, H. Herzel, A. Radbruch, E. Krause, R. Baumgrass, Identification of novel nuclear factor of activated T cell (NFAT)-associated proteins in T cells. *J. Biol. Chem.* **291**, 24172–24187 (2016).

101. M. Ichida, T. Finkel, Ras regulates NFAT3 activity in cardiac myocytes. *J. Biol. Chem.* **276**, 3524–3530 (2001).

102. H. Göös, M. Kinnunen, K. Salokas, Z. Tan, X. Liu, L. Yadav, Q. Zhang, G.-H. Wei, M. Varjosalo, Human transcription factor protein interaction networks. *Nat. Commun.* **13**, 766 (2022).

103. M. Yu, Z. Peng, M. Qin, Y. Liu, J. Wang, C. Zhang, J. Lin, T. Dong, L. Wang, S. Li, Y. Yang, S. Xu, W. Guo, X. Zhang, M. Shi, H. Peng, X. Luo, H. Zhang, L. Zhang, Y. Li, X.-P. Yang, S. Sun, Interferon-γ induces tumor resistance to anti-PD-1 immunotherapy by promoting YAP phase separation. *Mol. Cell* **81**, 1216–1230.e9 (2021).

104. S. Shu, C. Y. Lin, H. H. He, R. M. Witwicki, D. P. Tabassum, J. M. Roberts, M. Janiszewska, S. J. Huh, Y. Liang, J. Ryan, E. Doherty, H. Mohammed, H. Guo, D. G. Stover, M. B. Ekram, J. Brown, C. D'Santos, I. E. Krop, D. Dillon, M. McKeown, C. Ott, J. Qi, M. Ni, P. K. Rao, M. Duarte, S.-Y. Wu, C.-M. Chiang, L. Anders, R. A. Young, E. Winer, A. Letai, W. T. Barry, J. S. Carroll, H. Long, M. Brown, X. S. Liu, C. A. Meyer, J. E. Bradner, K. Polyak, Response and resistance to BET bromodomain inhibitors in triple-negative breast cancer. *Nature* **529**, 413–417 (2016).

105. J. You, J. L. Croyle, A. Nishimura, K. Ozato, P. M. Howley, Interaction of the bovine papillomavirus E2 protein with Brd4 tethers the viral DNA to host mitotic chromosomes. *Cell* **117**, 349–360 (2004).

106. Y. Nihongaki, S. Yamamoto, F. Kawano, H. Suzuki, M. Sato, CRISPR-Cas9-based photoactivatable transcription system. *Chem. Biol.* **22**, 169–174 (2015).

107. A. Rademacher, F. Erdel, J. Trojanowski, S. Schumacher, K. Rippe, Real-time observation of light-controlled transcription in living cells. *J. Cell Sci.* **130**, 4213–4224 (2017).

108. C. A. Schneider, W. S. Rasband, K. W. Eliceiri, NIH Image to ImageJ: 25 years of image analysis. *Nat. Methods* **9**, 671–675 (2012).

109. A. E. Carpenter, T. R. Jones, M. R. Lamprecht, C. Clarke, I. H. Kang, O. Friman, D. A. Guertin, J. H. Chang, R. A. Lindquist, J. Moffat, P. Golland, D. M. Sabatini, CellProfiler: Image analysis software for identifying and quantifying cell phenotypes. *Genome Biol.* **7**, R100 (2006).
